# Genetically Predicted C-Reactive Protein Associated With Postmenopausal Breast Cancer Risk: Interrelation With Estrogen and Cancer Molecular Subtypes Using Mendelian Randomization

**DOI:** 10.3389/fonc.2020.630994

**Published:** 2021-02-03

**Authors:** Su Yon Jung, Jeanette C. Papp, Eric M. Sobel, Matteo Pellegrini, Herbert Yu, Zuo-Feng Zhang

**Affiliations:** ^1^ Translational Sciences Section, Jonsson Comprehensive Cancer Center, School of Nursing, University of California, Los Angeles, Los Angeles, CA, United States; ^2^ Department of Human Genetics, David Geffen School of Medicine, University of California, Los Angeles, Los Angeles, CA, United States; ^3^ Department of Computational Medicine, David Geffen School of Medicine, University of California, Los Angeles, Los Angeles, CA, United States; ^4^ Department of Molecular, Cell and Developmental Biology, Life Sciences Division, University of California, Los Angeles, Los Angeles, CA, United States; ^5^ Cancer Epidemiology Program, University of Hawaii Cancer Center, Honolulu, HI, United States; ^6^ Department of Epidemiology, Fielding School of Public Health, University of California, Los Angeles, Los Angeles, CA, United States; ^7^ Center for Human Nutrition, David Geffen School of Medicine, University of California, Los Angeles, CA, United States

**Keywords:** genetically driven C-reactive protein, Mendelian randomization, obesity, exogenous estrogen, breast cancer subtypes by hormone receptor and HER2/neu

## Abstract

**Background:**

Immune-related etiologic pathways that influence breast cancer risk are incompletely understood and may be confounded by lifestyles or reverse causality. Using a Mendelian randomization (MR) approach, we investigated the potential causal relationship between genetically elevated C-reactive protein (CRP) concentrations and primary invasive breast cancer risk in postmenopausal women.

**Methods:**

We used individual-level data obtained from 10,179 women, including 537 who developed breast cancer, from the Women’s Health Initiative Database for Genotypes and Phenotypes Study, which consists of five genome-wide association (GWA) studies. We examined 61 GWA single-nucleotide polymorphisms (SNPs) previously associated with CRP. We employed weighted/penalized weighted–medians and MR gene–environment interactions that allow instruments’ invalidity to some extent and attenuate the heterogeneous estimates of outlying SNPs.

**Results:**

In lifestyle-stratification analyses, genetically elevated CRP decreased risk for breast cancer in exogenous estrogen-only, estrogen + progestin, and past oral contraceptive (OC) users, but only among relatively short-term users (<5 years). Estrogen-only users for ≥5 years had more profound CRP-decreased breast cancer risk in dose–response fashion, whereas past OC users for ≥5 years had CRP-increased cancer risk. Also, genetically predicted CRP was strongly associated with increased risk for hormone-receptor positive or human epidermal growth factor receptor-2 negative breast cancer.

**Conclusions:**

Our findings may provide novel evidence on the immune-related molecular pathways linking to breast cancer risk and suggest potential clinical use of CRP to predict the specific cancer subtypes. Our findings suggest potential interventions targeting CRP–inflammatory markers to reduce breast cancer risk.

## Introduction

Invasive breast carcinoma in postmenopausal women ages 50 years and older accounts for the majority (approximately 80%) of new breast cancer cases and related deaths (1). The probability of newly diagnosed patients at 50 years and older is twice as large as that of those younger than 50 (4.3 *vs*. 2.0%) (2), contributing to breast cancer ranking highest in cancer incidence in women of the United States and worldwide (2). Chronic inflammation may be a critical factor in the pathogenesis of obesity-associated cancers, such as postmenopausal breast cancer, from tumor initiation to progression (3). Adipose tissue increases macrophage infiltration that forms “crown-like structures” (CLSs) surrounding adipocytes, leading to a condition of chronic low-grade inflammation (4). The innate immunity response elevates the circulating levels of cancer-promoting inflammatory cytokines, creating a tissue microenvironment high in reactive oxygen and nitrogen species, leading to potential DNA damage and alterations in nearby cells (5). In particular, C-reactive protein (CRP) is a key pro-inflammatory biomarker, acting as a major acute-phase reactant and a biomarker of chronic low-grade inflammation, which has been implicated in carcinogenesis (3). For example, CRP, together with inflammatory cells and mediators, creates a pro-neoplastic environment for tumor growth by inducing DNA damage and promoting angiogenesis (5). Thus, circulating CRP levels reflect the magnitude of inflammation in the microenvironment that is favorable to tumor development and, particularly the levels measured at diagnosis, are associated with larger tumor size, lower tumor grade, and presence of metastasis (6).

For postmenopausal breast cancer, obesity is a well-established risk factor (7), and CLSs are frequently found in the breasts of obese women, the extent of which is correlated with adipocyte size, thus reflecting the severity of pro-tumorigenic inflammation in breasts (8). Enhanced secretion of CRP in these obese conditions could be involved in breast cancer development and progression, but the molecular mechanisms are only partially understood. In detail, cyclooxygenase-2 (COX-2) levels increase in inflamed breast tissue of obese women and directly contribute to increased aromatase activity which plays a critical role in breast carcinogenesis and, in turn, stimulates proliferation of the tumorous breast epithelium (8). CRP levels are reduced when COX-2 action is inhibited (9), suggesting that obesity-related aromatase activity in breast tumorigenesis correlates with inflammation. In addition to the local control of estrogen synthesis in breast tissue, the CRP-inflammatory marker can cause a systemic increase in circulating estrogen levels in obese women (10). Previous observational and genetic epidemiologic studies, however, yielded conflicting findings for an association between circulating CRP and breast cancer, with mostly null results (11–13) and a few positive associations with weak or modest strength (14, 15).

A Mendelian randomization (MR) approach may provide better insight into the immune-related etiologic pathways connected to invasive breast cancer risk. MR evaluates the exposure (*e.g.*, CRP) on an outcome (*e.g.*, breast cancer risk) using genetic variants as an instrumental variable (16). This approach may provide a relatively unbiased causal inference between CRP and breast cancer risk, because (i) MR can reduce potential confounding given random assignment of exposure owing to randomly assorted relevant genetic alleles at the time of gamete formation; the alleles are thus generally unrelated to environmental factors, and (ii) MR can address short-term exposures to inflammatory biomarkers by using the associated alleles as a proxy for lifelong exposure (16, 17). MR may also reduce the possibility of reverse causation in that alleles precede the phenotype and relevant clinical outcomes (17). Thus, in an MR framework, the elevated CRP concentrations are less likely to result from the immune response induced by premalignant or preclinical tumor growth. Finally, an MR study could be comparable to randomized clinical trials in providing a robust causal relationship if the genetic instruments are valid and not linked to cancer outcomes *via* alternative pathways other than those of the CRP phenotype (16).

In this study, we performed MR analysis by focusing on postmenopausal women who are vulnerable to a high incidence of inflammation (18), obesity, and breast cancer. In our earlier genome-wide association (GWA) meta-analysis of gene–environment (G×E) interaction study for CRP (19), we used data from the Database for Genotypes and Phenotypes (dbGaP) on a large-scale cohort of postmenopausal women in the Women’s Health Initiative (WHI). In addition to including the single-nucleotide polymorphisms (SNPs) identified in our previous GWA study (19), we have now extended the scope of modeled genetic instruments by adding GWA SNPs for CRP from previous GWA studies that targeted European ancestry with independent replications (20–22). We employed recently developed MR analyses, including weighted median (WM) and penalized weighted median (PWM) estimates (23, 24) and MR G×E interactions (25) to allow some relaxation on the strict rules for MR instrumental variables, to down weight the contribution of heterogeneity of genetic variants to analysis, and to incorporate interactions between genes and obesity-/sex hormone-related lifestyles in the MR analysis. We tested the hypothesis that genetically determined CRP that interacts with lifestyles and breast cancer molecular subtypes has a potentially causal association with invasive breast cancer risk.

## Materials and Methods

### Study Population

We used data from the WHI dbGaP Harmonized and Imputed GWA Studies (GWASs) which were coordinated to contribute to a joint imputation and harmonization effort for GWASs. Those studies, under dbGaP study accession number (phs000200.v12.p3), consist of five GWASs (AS264, GARNET, GECCO, HIPFX, and WHIMS) (**Table S1**) and encompass the two WHI representative study arms, Clinical Trials and Observational Studies, representing one of the largest studies on postmenopausal women in the U.S. to date. The detailed study designs and rationale are described elsewhere (26). Healthy women were enrolled in the WHI study between 1993 and 1998 at 40 clinical centers across the U.S if they were 50–79 years old, postmenopausal, expected to stay near the clinical centers for at least 3 years after enrollment, and able to provide written informed consent. Participants were further eligible for the WHI dbGaP study if they had met eligibility requirements for data submission to dbGaP and provided DNA samples. Of a total of 16,088 women who reported their race or ethnicity as non-Hispanic white, we applied exclusion criteria (history of diabetes, genomic data quality control, less than 1 year follow-up of cancer outcomes, and diagnosis of any cancer type at screening), resulting in a total of 10,179 women (**Table S1** includes the number of participants in each study). They had been followed up through August 29, 2014, a mean of 16 years’ follow-up and 537 (5% of the eligible 10,179 women) developed primary invasive breast cancer. The studies were approved by the institutional review boards of each WHI clinical center and the University of California, Los Angeles.

### Lifestyle Variables and Breast Cancer Outcome

Participants’ demographic and lifestyle factors were collected at screening by self-administered questionnaires, and the collection process was monitored periodically for data quality assurance by the coordinating clinical centers. With 48 initially selected variables derived from literature review for their association with inflammation and breast cancer (27–29), we performed preliminary analyses including univariate and stepwise multiple regression analyses and a multicollinearity test and finally selected the following 15 variables for analysis: demographic and socioeconomic factors (age, education, and family income); a family history of breast cancer and depressive symptoms; lifestyle factors (cigarette smoking and physical activity); dietary factors [daily alcohol and saturated fatty acids (SFA) intake]; and reproductive history [age at menopause, durations of oral contraceptive (OC) use and of exogenous estrogen (E)-only and E plus progestin (E + P) use]. Also, anthropometric variables (height, weight, and waist/hip circumferences) that had been measured by trained staff were included. For stratification analyses, we used the following variables as potential effect modifiers on the basis of previous studies (30–38) and relevant cutoff values: 30 kg/m^2^ body mass index (BMI), 0.85 waist-to-hip ratio (WHR), 10·hours/week metabolic equivalent (MET), and 9% calories from SFA on the basis of obesity and related lifestyle guidelines (39–41); 0.06 on the depressive scale of the Center for Epidemiological Studies (42); 15 cigarettes smoked daily; 14 g (one drink for women) of alcohol intake daily (43); a 5-year interval of E-only/E + P use ranging from non-use to 15 years or longer; and a 5-year median of OC use.

Primary invasive breast cancer diagnosis as our outcome of interest was determined *via* a centralized review of medical charts and pathology and cytology reports by a committee of physicians. Cancer cases were coded using the National Cancer Institute’s Surveillance, Epidemiology, and End-Results guidelines (44). The time from enrollment to breast cancer development, censoring, or study end point was calculated and presented in years.

### Genotyping and Instrumental Variables

Genotyped data was extracted for this study from the WHI dbGaP Harmonized and Imputed GWASs. Details of genotyping and imputation and the data-cleaning process have been reported (19, 26, 45). In brief, DNA was obtained from blood samples at baseline and genotyped using several GWAS platforms (26). The genotypes were normalized to Genome Reference Consortium Human Build 37, and genomic imputation was performed *via* the 1000 Genomes reference panels (26). SNPs were checked for harmonization with pairwise concordance among all samples across the GWASs. Through the initial and second data quality-control steps, SNPs were included by filtering on a missing-call rate of <2%, a Hardy–Weinberg equilibrium of p ≥1E–04, and $\hat{R}\geq 0.6$ imputation quality (46), but individuals with unexpected duplicates, first- and second-degree relatives, and outliers on the basis of genetic principal components (PCs) were excluded.

We used four GWAS resources to select CRP-SNPs: one from our earlier GWAS using the WHI Harmonized and Imputed GWASs that examined CRP as a binary outcome, reflecting chronic low-grade inflammation status with >3.0 mg/L of CRP (47, 48). Using the same population of genetic instruments and cancer outcomes may reduce bias from the MR analysis of different population structures between exposure and outcome. Of 82 SNPs in total, we selected five index/independent SNPs that were not in linkage disequilibrium (*i.e.*, LD <0.3) (**Table S1**). The other three GWASs recently reported CRP SNPs analyzing CRP as a continuous variable that was naturally log-transformed (mg/L). They used different genotype and analytic strategies, such as HapMap-based 1000 Genomes imputed data analysis (20), genome-wide analysis of discovery panel combined with replication panel (22), and exome-wide common and low/rare coding variants search (21). Of the total 89 SNPs from the three studies, 16 SNPs overlap; the analysis results from the more recent study were selected. With LD <0.3, 61 independent SNPs (the five from our study plus 56 from other studies) were finally included in our analysis (**Table S1**). The allele associated with higher CRP level was assigned to an alternative (risk) allele, whereas the other as a reference allele for all SNPs.

### Statistical Analysis

Three basic assumptions are necessary for a genetic variant to be valid in MR analysis: (i) the variant is robustly predictive of the exposure; (ii) the variant is independent of factors that confound the exposure–outcome association; and (iii) the variant is independent of the outcome, given the exposure and confounding factors of the exposure–outcome association (*i.e.*, the variant has no pleiotropic pathways other than the exposure) (49). We checked whether our data met the assumptions for a valid inference. The first assumption was addressed by selecting only SNPs that were associated with CRP at genome-wide significance. The inter-individual variability of CRP explained by all of the selected SNPs combined was about 6% (19, 21, 22) and, on the basis of sample size and number of instruments (50), the F-statistic was 108.15. Given the traditional threshold of 10 (51), we considered that our SNPs had sufficient strength. The second and third assumptions cannot be fully empirically tested because they depend on all confounders, both measured and unmeasured (24). For the horizontal pleiotropic effect, we excluded pleiotropic GWA SNPs (**Table S2**) whose relevant phenotype can be associated with CRP exposure and breast cancer outcome, including obesity (BMI and WHR), diabetic syndromes and diabetes (fasting glucose and insulin, post 2-h glucose, and type 2 diabetes [T2DM]), and dyslipidemia (low-/high-density lipoprotein, total cholesterol, and triglycerides) (16, 52). For our five GWA SNPs, there was no overlap with those pleiotropic SNPs, while four SNPs (in relation to BMI, post 2-h glucose, T2DM, and dyslipidemia) were excluded from the 56 outside GWA SNPs. In addition, we adjusted for potential confounding factors (listed in the Lifestyle variables subsection, above) in the analysis for the association between SNPs and breast cancer risk. We further conducted a MR-Egger regression analysis to test for vertical directional pleiotropy (the third assumption) and checked whether the pleiotropic SNPs were skewed in one direction rather than being balanced (53).

We conducted the MR analysis separately according to the CRP variable type analyzed in the GWASs: binary chronic inflammation status or continuous levels. In addition to a traditional inverse-variance weighed (IVW) method (54), we employed recently developed MR approaches such as WM/PWM estimates (23, 24) and MR G ×E interactions (25). The WM estimate allows up to 50% of genetic variants’ invalidity (*i.e.*, the assumptions violated) and provides a more consistent estimate of the causal effect if the precision of the individual estimates varies considerably, by assigning a weight to the ordered estimate and establishing linearity between neighboring estimates (24). When the estimates from invalid instruments are not balanced about the true effect, the WM, however, is inappropriate; the PWM estimate can minimize this issue by down-weighting outlying genetic variants with heterogeneous estimates (24). The PWM may also be a better parameter if there is directional pleiotropy. In each MR analysis, we performed hypothesis-driven, stratified analyses defined by potential effect modifiers, including lifestyle factors and breast cancer molecular subtypes. Further, we calculated a corrected MR estimate by taking into account the interaction of genes with selected obesity-related factors (BMI, WHR, MET, % calories from SFA, alcohol, and depressive symptoms) and sex hormone-lifestyles (E-only, E + P, and OC use) by applying the MR G ×E method (25). We created a weighted genetic score (GS) for that analysis using a polygenic additive model (55) with the 56 CRP-SNPs from previous GWASs that analyzed CRP as a continuous variable. We then rescaled the GS to the unit of CRP by performing a linear regression among women without breast cancer; by using β0 (slope) and *β*1 (intercept), we computed the scaled CRP-GS (= *β*0 + *β*1×GS), where two GSs were perfectly correlated (r = 1.0) (23, 55).

In the MR analysis for the five SNPs from our earlier GWAS, we adjusted a correlation between CRP phenotype and breast cancer in which exposure and outcome were evaluated within the same population. For parameters necessary for the MR analysis, the change in CRP (>3.0 *vs*. ≤3.0 mg/L) in log-odds and the mean change in log-transformed CRP per allele were obtained from our and the three other previous GWASs, respectively. The effect of genetic variants on breast cancer risk was calculated in our study population by using Cox regression with adjustment for (i) age and 10 PCs and (ii) lifestyle covariates in addition to age and 10 PCs. The assumption test was conducted *via* a Schoenfeld residual plot and ρ evaluation. The Cox results from each of our five GWASs were combined using fixed-effect meta-analysis. The final MR results were reported as risk ratios [particularly, hazard ratios (HRs)] and 95% confidence intervals (CIs) for the change in breast cancer risk per unit increase in log-odds or log-transformed CRP.

The heterogeneity of the MR estimate, reflecting additional evidence of pleiotropy, was estimated using Cochran’s Q test. A two-tailed p <0.05 was considered statistically significant. R3.6.3 (survival, metafor, forestplot, ggplot2, and ggthemes packages) was used.

## Results

**Figure 1:**
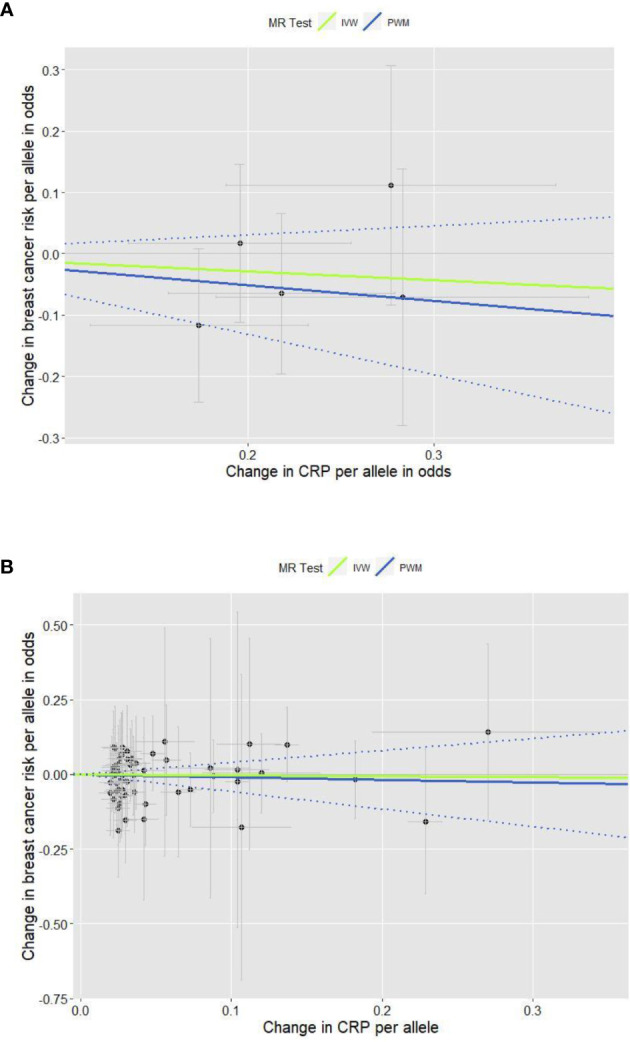
Scatter plots for the effects of individual CRP-genetic instrumental variables on breast cancer risk. Each black dot reflects a genome-wide CRP-raising genetic variant. The blue lines indicate penalized weighted median estimates and 95% CIs. (CI, confidence interval; CRP, C-reactive protein; HR, hazard ratio; IVW, inverse-variance weighted; MR, Mendelian randomization; PWM, penalized weighted median; SNP, single-nucleotide polymorphism.) **(A)** Five genome-wide SNPs associated with high immune response and chronic inflammation (CRP > 3.0 mg/L) (PWM HR_2nd-stage_ = 0.78, 95% CI: 0.52–1.16; MR-Egger intercept p value = 0.317). **(B)** Fifty-six genome-wide SNPs associated with CRP phenotype that was naturally log-transformed (mg/L) (PWM HR_2nd-stage_ = 0.91, 95% CI: 0.56–1.49; MR-Egger intercept p value = 0.391).

The total of 61 GWA SNPs identified for their association with CRP concentrations are presented in **Table S1**; five were from our GWAS analyzing binary CRP outcomes (>3.0 *vs*. ≤3.0 mg/L, reflecting low-grade chronic inflammation status), and the other 56 from three other previous GWASs examining continuous natural log-transformed CRP (mg/L). The associations between each of the 61 SNPs and breast cancer risk are shown in **Table S3**, including results from the first stage of adjustment for age and 10 PCs and from the second stage of adjustment for lifestyle covariates in addition to age and 10 PCs. The pooled analysis for the genetic instruments combining five SNPs (**Figure 1A**) and 56 SNPs (**Figure 1B**) each did not reveal statistically significant associations (the five SNPs’ PWM-HR_2nd-stage_ = 0.78, 95% CI: 0.52–1.16; the 56 SNPs’ PWM-HR_2nd-stage_ = 0.91, 95% CI: 0.56–1.49). Removing pleiotropic SNPs did not apparently change the pooled estimates.

**Figure 2:**
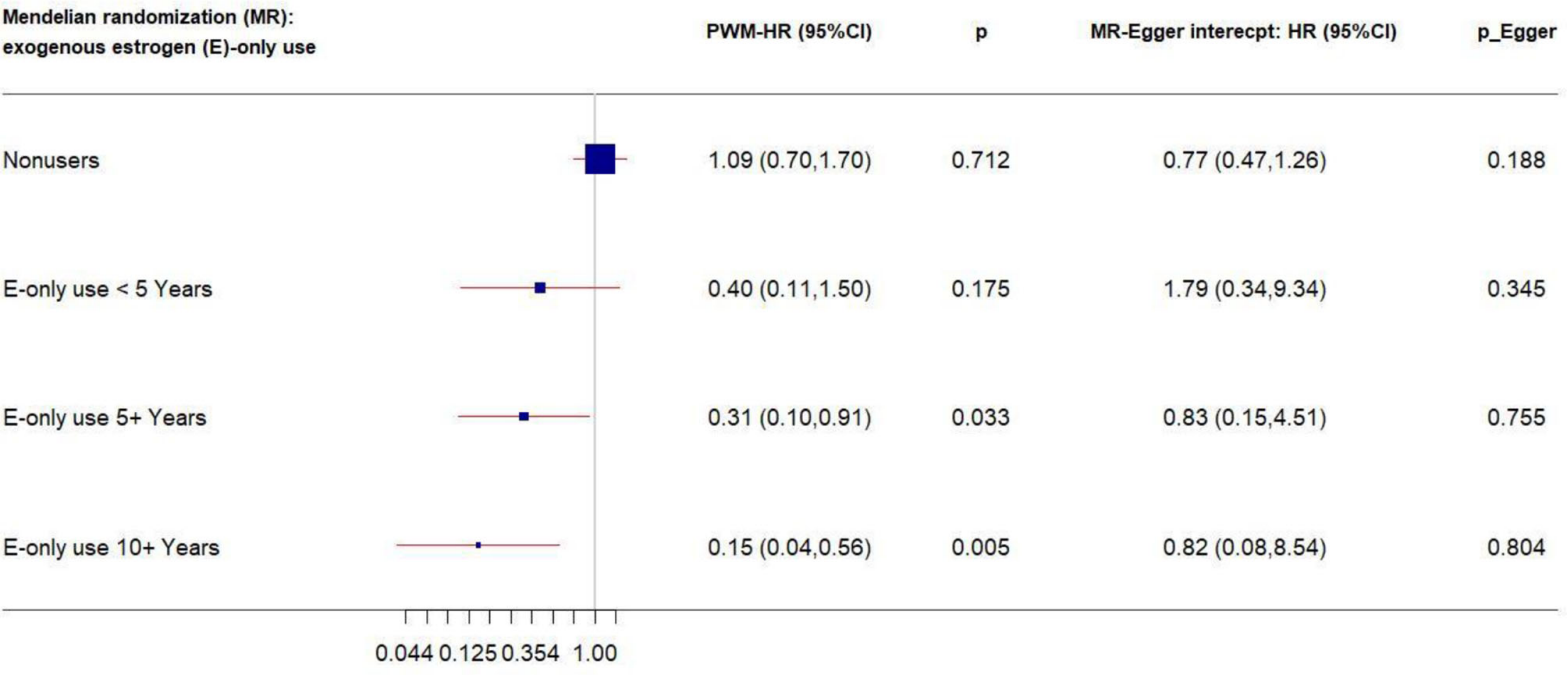
Forest plot of MR estimates by E-only use. The plot shows the effects of genetically predicted chronic inflammation status (CRP > 3.0 mg/L) on breast cancer risk in E-only user subgroups, presented as the 95% CIs (red lines) of the estimates and the penalized weighted medians (percentages proportional to the size of the blue squares). The MR estimates were based on the SNP–breast cancer association that was adjusted for age and 10 principal components only. *p* values were adjusted to correct for multiple testing *via* the Benjamini–Hochberg approach. (CI, confidence interval; E, exogenous estrogen; HR, hazard ratio; MR, Mendelian randomization; PWM, penalized weighted median.)

We performed stratification analysis defined by obesity-related factors, lifestyles, a family history of breast cancer, depressive symptoms, and exogenous estrogen use. In the MR analysis of our five CRP-SNPs, a 1-unit increase in the genetically predicted chronic inflammation (defined as >3.0 mg/L of CRP) was associated with approximately 80% decreased risk of breast cancer among E + P users, particularly in women with <5-years’ use of E + P (PWM-HR_1st-stage_ = 0.17, 95% CI: 0.05–0.63) (**Table 1**). The reduced effect on breast cancer risk was more profound in stage 2 of the MR analysis, which used the SNP–cancer association adjusted for lifestyles in addition to age and 10 PCs. Likewise, genetically determined chronic inflammation status was associated with reduced risk for breast cancer among E-only users. In particular, the cancer risk was reduced by 50% in E-only users for <5 years and more substantially decreased in longer-term users, showing a dose-response relationship (**Figure 2**). Of note, this reduced risk of cancer in E-only users was present only in the first MR stage. The MR-Egger test showed no significant evidence of directional pleiotropy across the tested associations (**Table 1** and **Figure 2**).

**Table 1:** Mendelian randomization analysis: the effect of genetically predicted chronic inflammation status on breast cancer risk by exogenous estrogen plus progestin use.

GWAS examining CRP as a binary outcome reflecting high immune response and chronic inflammation (CRP > 3.0 mg/L)								
Analytic method	Stage 1				Stage 2			
	Adjustment for age and 10 PCs				Adjustment for covariates * in addition to age and 10PCs			
	HR^¶^	(95% CI)	p^§^	p-het^†^	HR^¶^	(95% CI)	p^§^	p-het^†^
**<Exogenous estrogen plus progestin non-users>**								
Inverse-variance weighted	1.02	(0.69–1.50)	0.892	0.652	0.95	(0.65–1.40)	0.735	0.656
Weighted median	0.96	(0.62–1.49)	0.860		0.93	(0.60–1.44)	0.753	
Penalized weighted median	0.96	(0.63–1.46)	0.854		0.93	(0.60–1.45)	0.756	
MR-Egger: slope	2.98	(0.38–23.60)	0.192		2.82	(0.36–21.83)	0.206	
intercept	0.79	(0.50–1.24)	0.193		0.79	(0.50–1.23)	0.185	
**<Exogenous estrogen plus progestin use <5 years>**								
Inverse-variance weighted	0.27	(0.04–1.65)	0.115	0.110	0.00	(0.00–2.76E+17)	0.305	<0.005
Weighted median	**0.19**	**(0.05–0.69)**	**0.011**		**0.03**	**(0.003–0.20)**	**<0.005**	
Penalized weighted median	**0.17**	**(0.05–0.63)**	**0.007**		0.02	(0.00–1.02)	0.051	
MR-Egger: slope	0.05	(0.00–18942.43)	0.512		0.00	(0.00–1.05E+87)	0.205	
intercept	1.46	(0.09–24.79)	0.700		1.13E+17	(0.00–4.09E+55)	0.254	

CI, confidence interval; CRP, C-reactive protein; GWAS, genome-wide association study; HR, hazard ratio; MR, Mendelian randomization; PCs, principal components; SFA, saturated fatty acids; SNP, single-nucleotide polymorphism. Numbers in bold face are statistically significant.

*Covariates adjusted in the analyses for the association between genome-wide SNPs and breast cancer risk include education; annual family income; family history of breast cancer; body mass index; waist-to-hip ratio; physical activity; depressive symptoms; number of cigarettes per day; dietary alcohol in g/day; % calories from SFA/day; age at menopause; duration of oral contraceptive use; and duration of exogenous estrogen-only use.

^§^p values were adjusted to correct for multiple testing via the Benjamini–Hochberg approach.

^¶^The Mendelian randomization estimate (except weighted/penalized weighted medians) was adjusted for a correlation between CRP phenotype and breast cancer risk within the same population.

^†^Heterogeneity in estimates among genome-wide SNPs was evaluated with Cochran’s Q test with fixed effects.

**Figure 3:**
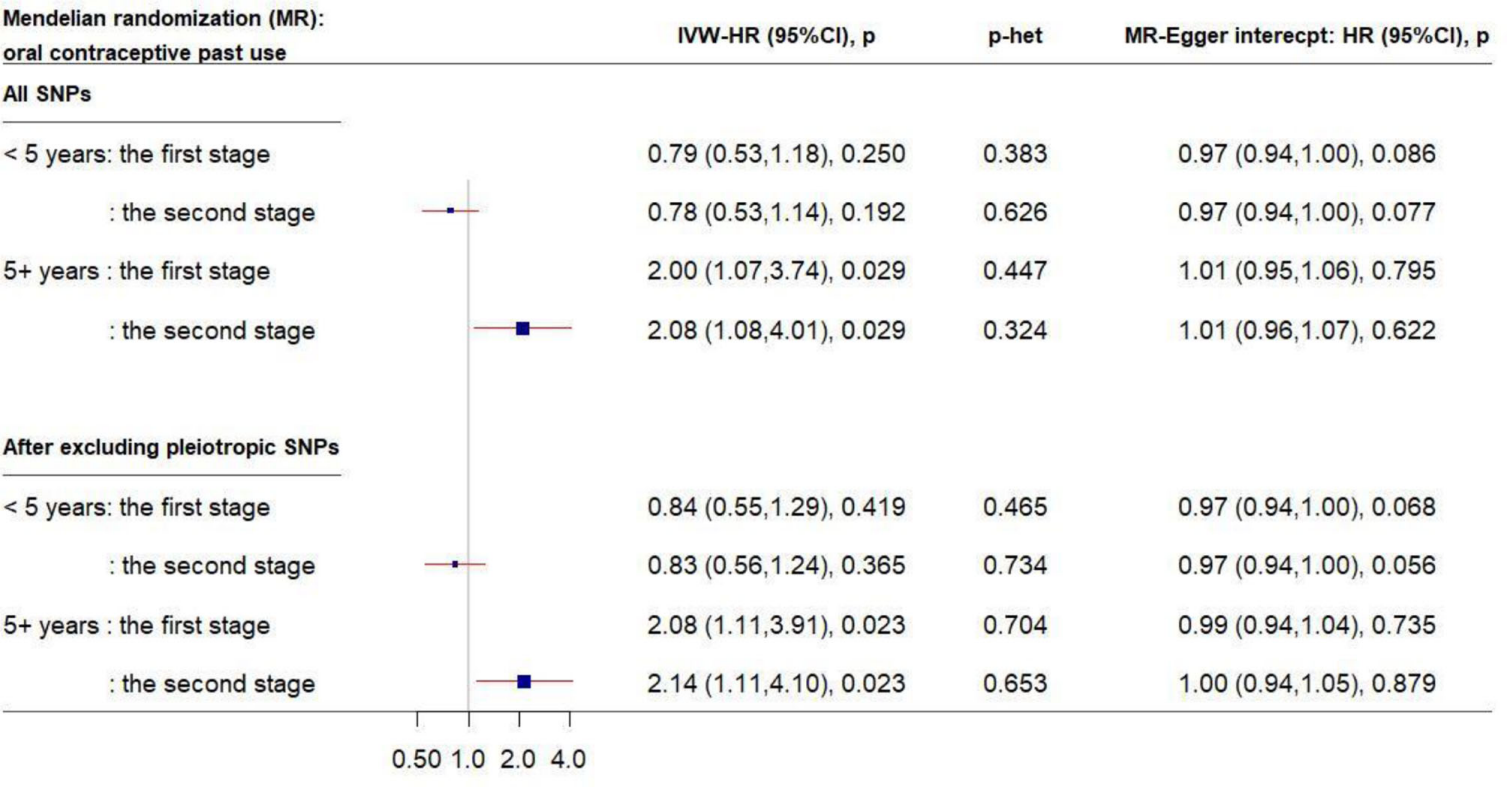
Forest plot of MR estimates by past OC use. The plot shows the effects of genetically predicted CRP phenotype on breast cancer risk in OC user subgroups, presented as the 95% CIs (red lines) of the estimates and the inverse-variance weights (percentages proportional to the size of the blue squares). The MR estimates were based on the SNP–breast cancer association that was adjusted for 1) only age and 10 principal components (PCs) in the first stage and 2) lifestyle covariates in addition to age and 10 PCs in the second stage. *p* values were adjusted to correct for multiple testing *via* the Benjamini–Hochberg approach. (CI, confidence interval; CRP, C-reactive protein; HR, hazard ratio; IVW, inverse-variance weighted; MR, Mendelian randomization; OC, oral contraceptive; SNP, single-nucleotide polymorphism.)

Similarly, in the MR analysis of the other 56 SNPs, a 1-unit increase in the log-transformed genetically elevated CRP was associated with about 20% reduced risk for breast cancer among women who had used OC for <5 years (**Figure 3**); the estimates remained consistent in the second stage of MR analysis after excluding pleiotropic SNPs. However, a different pattern was observed among longer-term past OC users. Genetically elevated CRP levels were strongly associated with increased breast cancer risk in past OC users for ≥5 year (IVW-HR_2nd-stage_ = 2.14, 95% CI: 1.11–4.10, after exclusion of pleiotropic SNPs). No directional pleiotropy was observed.

In addition, we stratified breast cancer patients by molecular subtype [estrogen and progesterone receptor (ER/PR) and human epidermal growth factor receptor 2 (HER2/neu) status]. A 1-unit increase in the log-transformed genetically elevated CRP level was associated with 80% or more increased risk for ER/PR-positive breast cancer (IVW-HR_1st-stage_ = 1.79, 95% CI: 1.17–2.76; WM-HR_2nd-stage_ = 2.11, 95% CI: 1.00–4.46) (**Table 2**). The IVW estimate in the first stage remained statistically significant after exclusion of pleiotropic SNPs (**Table 2**), with no evidence of heterogeneous and directional pleiotropic effects of the SNPs. Of note, a stronger increased risk of breast cancer was observed for the genetic instruments of CRP in HER2/neu-negative breast cancer. For example, women with genetically elevated CRP were three times more likely to develop HER2/neu-negative breast cancer (PWM-HR_2nd-stage_ = 3.20, 95% CI: 1.27–8.08, after exclusion of pleiotropic SNPs) (**Table 2**). No other subgroups revealed a statistically significant association between genetically determined chronic inflammation or CRP levels and breast cancer risk (**Tables S4** and **S5**).

**Table 2:** Mendelian randomization analysis: the effect of genetically predicted CRP phenotype on breast cancer risk by ER/PR and HER2/neu status.

GWASs analyzing CRP as a continuous variable that was naturally log-transformed (mg/L)																		
Analytic method	All SNPs										After exclusion of pleiotropic SNPs							
	Stage 1 Adjustment for age and 10 PCs				Stage 2 Adjustment for covariates * in addition to age and 10PCs						Stage 1 Adjustment for age and 10 PCs				Stage 2 Adjustment for covariates * in addition to age and 10PCs			
	HR	(95% CI)	p^§^	p-het^†^	HR	(95% CI)			p^§^	p-het^†^	HR	(95% CI)	p^§^	p-het^†^	HR	(95% CI)	p^§^	p-het^†^
**<ER/PR positive>**																		
Inverse-variance weighted	**1.79**	**(1.17–2.76)**	**0.009**	0.209	1.81	(0.97–3.39)			0.064	<0.005	**1.80**	**(1.11–2.93)**	**0.018**	0.129	1.68	(0.83–3.41)	0.149	<0.005
Weighted median	1.72	(0.97–3.07)	0.065		**2.11**	**(1.00–4.46)**			**0.049**		1.85	(0.99–3.47)	0.055		2.08	(0.89–4.90)	0.093	
Penalized weighted median	1.77	(0.98–3.17)	0.057		2.10	(1.00–4.40)			0.050		1.89	(0.98–3.64) 0.056			2.07	(0.89–4.83)	0.091	
MR-Egger: slope	1.94	(1.02–3.69)	0.042		2.30	(0.91–5.81)			0.078		1.98	(0.96–4.10)	0.065		2.17	(0.75–6.27)	0.150	
intercept	0.99	(0.96–1.03)	0.731		0.98	(0.93–1.04)			0.484		0.99	(0.95–1.04)	0.730		0.98	(0.92–1.04)	0.517	
**<HER2/neu negative>**																		
Inverse-variance weighted	1.89	(1.15–3.10)	0.013	0.187	1.83	(0.91–3.67)			0.089	0.006	**2.09**	**(1.21–3.64)**	**0.010**	0.123	2.11	(0.97–4.60)	0.061	0.003
Weighted median	**2.07**	**(1.02–4.17)**	**0.043**		2.10	(0.87–5.08)			0.099		**2.80**	**(1.34–5.87)**	**0.006**		**3.23**	**(1.30–8.05)**	**0.012**	
Penalized weighted median	**2.13**	**(1.11–4.11)**	**0.024**		2.01	(0.83–4.85)			0.121		**3.00**	**(1.48–6.08)**	**0.002**		**3.20**	**(1.27–8.08)**	**0.014**	
MR-Egger: slope	**2.60**	**(1.25–5.41)**	**0.011**		2.08	(0.74–5.89)			0.163		**3.19**	**(1.41–7.23)**	**0.006**		2.78	(0.86–8.97)	0.087	
intercept	0.98	(0.93–1.02)	0.240		0.99	(0.93	–	1.05)	0.732		0.97	(0.93–1.01)	0.172		0.98	(0.92–1.05)	0.529	

CI, confidence interval; CRP, C-reactive protein; ER/PR, estrogen and progesterone receptor; GWAS, genome-wide association study; HER2/neu, human epidermal growth factor receptor 2; HR, hazard ratio; PCs, principal components; SFA, saturated fatty acids; SNP, single-nucleotide polymorphism. Numbers in bold face are statistically significant.

*Covariates adjusted in the analyses for the association between genome-wide SNPs and breast cancer risk include education; annual family income; family history of breast cancer; body mass index; waist-to-hip ratio; physical activity; depressive symptoms; number of cigarettes per day; dietary alcohol in g/day; % calories from SFA/day; age at menopause; duration of oral contraceptive use; and durations of exogenous estrogen [E]–only use and E plus progestin use.

^§^p values were adjusted to correct for multiple testing via the Benjamini-Hochberg approach.

^†^Heterogeneity in estimates among genome-wide SNPs was evaluated with Cochran’s Q test with fixed effects.

We further performed MR G × E analyses to estimate the corrected MR estimates by incorporating the G × E interactions with the selected obesity and sex-hormone lifestyle factors; none of the estimates reached statistical significance and no pleiotropic effect of the estimates was detected (**Table S6**).

## Discussion

We performed MR analyses for genetically predicted CRP levels (>3 mg/L *vs*. ≤3.0 mg/L, indicating chronic low-grade inflammation or a natural log-transformed 1 mg/L increase) in association with postmenopausal breast cancer risk and showed that genetically determined CRP levels were associated with the risk for breast cancer in women with particular lifestyle factors and breast cancer subtypes. MR findings, if the modeled genetic instruments are not linked to the outcome *via* any alternative pathway and are not associated with confounders of the exposure–outcome association, may be comparable with those of randomized clinical trials (54), thus providing a robust causal inference. Our MR analysis reduced the pleiotropic effect by identifying a wide range of confounding factors that are connected to the CRP–breast cancer pathway and accounting for them in the analysis of the genetic instrument–cancer outcome association, and by removing the pleiotropic SNPs that may confound the association between CRP and breast cancer. Our MR estimates, including WM and PWM, allow some relaxation of the restrictions on instrumental variables, and thus provide a more robust estimate of the causal effect than a traditional MR estimate. To our knowledge, this study is the first to report the causal effect of genetically elevated CRP levels on increased breast cancer risk in an MR framework.

Most previous epidemiological studies examining the measured CRP levels showed no significant association with breast cancer risk (11–13), despite the potential role of CRP in breast cancer carcinogenesis both systemically and locally. As pointed out in the previous studies, reverse causation could not be ruled out. For example, the chronic inflammatory status with elevated CRP levels may be involved in cancer cell initiation and growth (13, 56), but it may also be the consequence of tumor progression, as shown by the infiltration of CD4+ and CD8+ regulatory T lymphocytes in breast cancer tissues that is associated with poor cancer survival (57). Thus, several studies have showed the effect of high CRP levels as leading to a worse prognosis after the diagnosis of breast cancer (6, 13). In addition, CRP levels are easily influenced by various modifiable and non-modifiable factors such as physiologic and pathologic stimuli, reflecting the inconclusiveness of one or a few time measurements.

An MR study is not likely to be susceptible to reverse causation and potential confounding owing to a random assortment of the genetic alleles at the time of gamete formation before the disease onset. Further, MR can allow the assessment of a long-standing effect of CRP on cancer development. Until now, we have found only one published MR study on the CRP phenotype and breast cancer risk (58). That study used four SNPs in the *CRP* gene with nine genotype combinations and adjusted for lifestyle confounding; no significant association was reported. Our study utilized 61 CRP-associated SNPs from the most recently updated GWASs and, with no evidence of violation for weak genetic instruments and directional pleiotropy, we conducted MR analyses with two separate stages of lifestyle adjustments.

We further conducted stratification analyses by obesity, sex-hormone and breast cancer subtype to determine whether these lifestyle and pathologic factors modified the association between genetically elevated CRP and breast cancer risk. We detected a substantially reduced risk of breast cancer in relation to CRP in E-only, E + P, and past OC users, but only among relatively short-term users (<5 years). In particular, longer-term E-only users (≥5 years) had more profound CPR-decreased cancer risk, in dose-response fashion. This finding is not align with our other finding of CRP-increased ER/PR-positive breast cancer risk. It may reflect the different effect of estrogen on cancer risk when it is taken orally, and our finding is supported by those of previous studies (59, 60) showing that oral intake of estrogen has its first-pass metabolic effect of suppressing hepatic production of insulin-like growth factor-I (IGF-I), a hormone partly interacting with CRP as a carcinogenic promotor, thus suggesting the protective role of exogenous estrogen in postmenopausal breast cancer risk (61). Altogether, that evidence and our findings suggest that longer-term use of E-only may be implicated in the breast cancer inhibitory pathway that is presumably involved in inflammation.

In contrast, E + P users have different levels of IGF-I and cancer risk owing to non-progesterone-like effects (*i.e.*, different effects from natural progesterone), contrasting with the hepatocellular effect of oral estrogen (62); but, the mechanism is not completely clear. In addition, synthetic progestin has an affinity for androgen and mineralocorticoid receptors, leading to cell proliferation and anti-apoptosis, contributing to breast carcinogenesis (63). In our MR study, only longer-term users of E + P (≥5 years) had CRP-increased risk for breast cancer, implying an effect of long-term cumulative exposure to synthetic progestin that interacts with inflammation, although this association did not reach statistical significance; that result warrants future studies with a larger population for more definitive results. Similarly, the women in our study who had used OC for ≥5 years in the past had a strongly CRP-increased risk for breast cancer. This result is consistent with previously published findings from other studies (64, 65) that showed increased breast cancer risk with long-term duration of OC use. The use of OC, especially those containing E+P, increases the proliferation of human breast epithelial cells (63), which may partially support our findings of increased cancer risk in long-term OC and E + P users. Our data sources had no information about the type of OC preparation our participants had taken; this calls for a future study that examines the potentially different effects on cancer risk according to specific OC formulations.

In addition, genetically elevated CRP in our study was strongly associated with increased risk for ER/PR-positive breast cancer, which is consistent with recent findings (66). Also, genetically determined CRP was associated with increased breast cancer risk in obese groups (BMI ≥ 30; WHR > 0.85), despite a lack of statistical power. Those findings suggest, in part, the potential existence of the inflammation-related pathway that may be involved with adiposity in hormone receptor–positive breast cancer development. Excessive adiposity characterized by adipocyte hypertrophy leads to chronic inflammation of adipose tissues, forming CLSs; the inflamed breast CLSs in turn produce inflammatory molecules such as CRP and other cytokines, leading to the activation of nuclear factor-kB that elevates aromatase production, thus logically driving hormone receptor–positive tumor growth (66). We also found that genetically predicted CRP was associated with increased risk of HER2/neu-negative breast cancer. In accord with the results of a few previous studies (56, 67), our finding supports a potential mechanism connecting inflammation to HER2/neu-negative breast cancer, in which pro-inflammatory markers trigger *JAK/STAT* signaling pathways, activating genes responsible for cell proliferation and angiogenesis, and those aberrant pathways then contribute to an immunosuppressive tumorigenic microenvironment, leading to more aggressive breast cancer such as basal-like tumors (13, 56, 67).

We performed a diagnostic test for MR directional pleiotropy and reduced any problematic pleiotropic effect by applying several statistical methods: removal of pleiotropic SNPs, adjustment for confounding, incorporation of the gene–lifestyle interactions (MR G×E tests and stratification), and use of WM/PWM estimates. Nevertheless, we cannot completely rule out residual and unmeasured confounding that might have affected our study findings. In addition, potential violation of a linearity assumption in the SNP–exposure and exposure–outcome analyses would result toward a null, rather than generating a spurious association (68). The genetic effects in this study were associated with the SNPs involved in CRP phenotype, one of the key pro-inflammatory biomarkers in the inflammatory cytokine pathways. Lastly, our study results may not be generalized to other races or ethnicity, in which the relationship between genetic instruments, CRP, and breast cancer risk may be different.

Although our MR study was not designed to discover biologic mechanisms, our findings suggest a potential causal relationship between genetically elevated CRP levels and risk of postmenopausal breast cancer, particularly in relatively short-term users of opposed and long-term users of unopposed exogenous estrogen. Also, lifetime exposure to elevated CRP levels is likely to influence the development of hormone receptor–positive and HER2/neu-negative breast cancer. Further biologic mechanistic study may help to elaborate the sex-hormone interactions with CRP on breast cancer carcinogenesis and the molecular pathways connected to CRP and ER/PR/HER2 receptors. Our findings may provide important novel evidence on immune-related etiologic pathways that interact with lifestyle factors, influencing breast cancer risk, and suggest the potential clinical use of CRP to predict specific cancer subtypes and CRP–inflammatory marker-targeting interventions to reduce breast cancer risk in postmenopausal women.

## Data Availability Statement

Publicly available datasets were analyzed in this study. This data can be found here: The data that support the findings of this study are available in accordance with policies developed by the NHLBI and WHI in order to protect sensitive participant information and approved by the Fred Hutchinson Cancer Research Center, which currently serves as the IRB of record for the WHI. Data requests may be made by emailing helpdesk@WHI.org.

## Ethics Statement

All the participant data for the WHI Harmonized and Imputed GWASs under the WHI dbGaP Study used in this study were de-identified by the NHLBI and WHI and consent was obtained from the participants at the source. The study was approved by the institutional review boards of each participating clinical center of the WHI and the University of California, Los Angeles. The patients/participants provided their written informed consent to participate in this study.

## Author Contributions

SJ, JP, ES, MP, HY, and Z-FZ designed the study. SJ performed the genomic data QC. SJ performed the statistical analysis and SJ, JP, ES, MP, and HY interpreted the data. ES and Z-FZ supervised the genomic data QC and analysis and participated in the study coordination and interpreting the data. SJ secured funding for this project. All participated in the paper writing and editing. All authors contributed to the article and approved the submitted version.

## Funding

This study was supported by the National Institute of Nursing Research of the National Institutes of Health under Award Number K01NR017852 and a University of California Cancer Research Coordinating Committee grant (CRN-18-522722).

## Conflict of Interest

The authors declare that the research was conducted in the absence of any commercial or financial relationships that could be construed as a potential conflict of interest.
