# Supplementary material for: Genetically Predicted C-Reactive Protein Associated With Postmenopausal Breast Cancer Risk: Interrelation With Estrogen and Cancer Molecular Subtypes Using Mendelian Randomization
Source: Front Oncol. 2021 Feb 3;10:630994. doi: 10.3389/fonc.2020.630994 (PMC7888276; doi:10.3389/fonc.2020.630994)
Supplement: Supplementary file 1 [file DataSheet_1.zip › TableS1_2020Dec15.docx]

Table 1. Genome-wide SNPs and their associations with CRP concentrations in previous GWASs

|  |  |  |  | **Alt allele frequency** | | | | | | | | |  |  |  |  |  |  |
| --- | --- | --- | --- | --- | --- | --- | --- | --- | --- | --- | --- | --- | --- | --- | --- | --- | --- | --- |
|  |  |  |  | **Allele*** | |  | **AS264** | **GARNET** | **GECCO** | **HIPFX** | | **WHIMS** |  |  |  |  |  |  |
| **SNP&** | **Chr** | **Position**¥ | **Gene** | **Ref** | **Alt** |  | **n=1,603** | **n=2,382** | **n=1,393** | **n=1,909** | **n=3,511** | |  | **OR/beta** | **SE** | **p** | **Q / Study (19-22)** |  |
|  | | | | | | | | | | | | | | | | | |  |
| GWAS examining CRP as a binary outcome reflecting high immune response and chronic inflammation (CRP > 3.0 mg/L) | | | | | | | | | | | | | | | | | |  |
|  | | | | | | | | | | | | | | | | | |  |
| rs2794520 | 1 | 159678816 | *CRPP1/CRP* | C | T |  | 0.34 | 0.34 | 0.33 | 0.33 | 0.33 | |  | 1.216† | 0.031 | 1.60E-10 | 3.00E-04 / Jung et al. |  |
| rs2243458 | 12 | 121424490 | *HNF1A* | C | T |  | 0.31 | 0.32 | 0.33 | 0.30 | 0.32 | |  | 1.244† | 0.031 | 2.26E-12 | 0.2637 / Jung et al. |  |
| rs1169311 | 12 | 121440731 | *C12orf43* | C | T |  | 0.35 | 0.37 | 0.36 | 0.34 | 0.36 | |  | 1.190† | 0.030 | 5.64E-09 | 0.3752 / Jung et al. |  |
| rs429358 | 19 | 45411941 | *APOE* | T | C |  | 0.15 | 0.13 | 0.13 | 0.13 | 0.14 | |  | 1.319† | 0.045 | 8.30E-10 | 0.0332 / Jung et al. |  |
| rs5117 | 19 | 45418790 | *APOC1* | T | C |  | NA | 0.21 | NA | NA | 0.22 | |  | 1.327† | 0.051 | 3.06E-08 | 0.7675 / Jung et al. |  |
|  | | | | | | | | | | | | | | | | | |  |
| GWASs analyzing CRP as a continuous variable that was naturally log-transformed (mg/L) | | | | | | | | | | | | | | | | | |  |
|  | | | | | | | | | | | | | | | | | |  |
| rs75460349 | 1 | 27180088 | *ZDHHC18* | C | A |  | 0.98 | 0.97 | 0.98 | 0.97 | 0.98 | |  | 0.086 | 0.014 | 4.50E-10 | Ligthart et al. |  |
| rs2293476 | 1 | 40036847 | *PABPC4* | G | C |  | 0.21 | 0.22 | 0.25 | 0.23 | 0.23 | |  | 0.030 | 0.004 | 8.27E-13 | Ligthart et al. |  |
| rs1805096 | 1 | 66102257 | *LEPR* | A | G |  | 0.63 | 0.62 | 0.65 | 0.63 | 0.63 | |  | 0.104 | 0.004 | 2.17E-183 | Ligthart et al. |  |
| rs469772 | 1 | 91530305 | *ZNF644* | T | C |  | 0.80 | 0.80 | 0.81 | 0.82 | 0.81 | |  | 0.031 | 0.005 | 5.54E-12 | Ligthart et al. |  |
| rs4129267 | 1 | 154426264 | *IL6R* | T | C |  | 0.59 | 0.60 | 0.60 | 0.60 | 0.59 | |  | 0.088 | 0.004 | 1.20E-129 | Ligthart et al. |  |
| rs2794520 | 1 | 159678816 | *CRPP1/CRP* | T | C |  | 0.66 | 0.66 | 0.67 | 0.67 | 0.67 | |  | 0.182 | 0.004 | 4.17E-523 | Ligthart et al. |  |
| rs1800947 | 1 | 159683438 | *CRP* | G | C |  | 0.94 | 0.94 | 0.94 | 0.94 | 0.95 | |  | 0.270 | 0.039 | 4.82E-12 | Schick et al. |  |
| rs1417938 | 1 | 159684186 | *CRP* | T | C |  | 0.30 | 0.31 | 0.32 | 0.32 | 0.32 | |  | 0.120 | 0.020 | 7.27E-09 | Schick et al. |  |
| rs10925027 | 1 | 247612562 | *NLRP3* | C | T |  | 0.40 | 0.40 | 0.42 | 0.40 | 0.39 | |  | 0.036 | 0.004 | 4.25E-21 | Ligthart et al. |  |
| rs12995480 | 2 | 629881 | *TMEM18* | T | C |  | 0.83 | 0.82 | 0.83 | 0.83 | 0.82 | |  | 0.031 | 0.005 | 1.24E-10 | Ligthart et al. |  |
| rs1260326 | 2 | 27730940 | *GCKR* | C | T |  | 0.41 | 0.41 | 0.41 | 0.41 | 0.40 | |  | 0.073 | 0.004 | 2.72E-92 | Ligthart et al. |  |
| rs4246598 | 2 | 88438050 | *FABP1* | C | A |  | 0.45 | 0.46 | 0.46 | 0.45 | 0.46 | |  | 0.022 | 0.004 | 5.11E-10 | Ligthart et al. |  |
| rs9284725 | 2 | 102744854 | *IL1R1* | A | C |  | 0.24 | 0.25 | 0.24 | 0.25 | 0.25 | |  | 0.027 | 0.004 | 7.34E-10 | Ligthart et al. |  |
| rs13409371 | 2 | 113838145 | *IL1F10* | G | A |  | 0.39 | 0.39 | 0.42 | 0.39 | 0.40 | |  | 0.048 | 0.004 | 5.07E-36 | Ligthart et al. |  |
| rs1441169 | 2 | 214033530 | *IKZF2* | G | A |  | 0.49 | 0.49 | 0.49 | 0.48 | 0.48 | |  | 0.025 | 0.004 | 2.27E-11 | Ligthart et al. |  |
| rs2352975 | 3 | 49891885 | *TRAIP* | T | C |  | NA | 0.31 | 0.30 | 0.31 | 0.31 | |  | 0.025 | 0.004 | 6.43E-10 | Ligthart et al. |  |
| rs1514895 | 3 | 170705693 | *EIF5A2* | A | G |  | 0.30 | 0.30 | 0.29 | 0.28 | 0.29 | |  | 0.027 | 0.004 | 2.70E-09 | Ligthart et al. |  |

Table 1 (Continued)

|  |  |  |  | **Alt allele frequency** | | | | | | | | |  |  |  |  |  |  |
| --- | --- | --- | --- | --- | --- | --- | --- | --- | --- | --- | --- | --- | --- | --- | --- | --- | --- | --- |
|  |  |  |  | **Allele*** | |  | **AS264** | **GARNET** | **GECCO** | **HIPFX** | | **WHIMS** |  |  |  |  |  |  |
| **SNP&** | **Chr** | **Position**¥ | **Gene** | **Ref** | **Alt** |  | **n=1,603** | **n=2,382** | **n=1,393** | **n=1,909** | **n=3,511** | |  | **OR/beta** | **SE** | **p** | **Q / Study (19-22)** |  |
|  | | | | | | | | | | | | | | | | | | |
| (Cont.) GWASs analyzing CRP as a continuous variable that was naturally log-transformed (mg/L) | | | | | | | | | | | | | | | | | |  |
|  | | | | | | | | | | | | | | | | | | |
| rs4705952 | 5 | 131839618 | *IRF1* | A | G |  | 0.25 | NA | 0.26 | 0.25 | 0.24 | |  | 0.042 | 0.007 | 1.30E-08 | Dehghan et al. |  |
| rs17658229 | 5 | 172191052 | *DUSP1* | T | C |  | 0.04 | NA | 0.04 | 0.04 | 0.04 | |  | 0.056 | 0.010 | 5.50E-09 | Ligthart et al. |  |
| rs9271608 | 6 | 32591588 | *HLA-DQA1* | A | G |  | 0.17 | NA | NA | NA | 0.16 | |  | 0.042 | 0.005 | 2.33E-17 | Ligthart et al. |  |
| rs12202641 | 6 | 116314634 | *FRK* | T | C |  | 0.59 | NA | 0.59 | 0.60 | 0.59 | |  | 0.023 | 0.004 | 3.00E-10 | Ligthart et al. |  |
| rs6901250 | 6 | 117114025 | *GPRC6A* | G | A |  | 0.33 | 0.32 | 0.32 | 0.33 | 0.31 | |  | 0.035 | 0.006 | 4.80E-08 | Dehghan et al. |  |
| rs1490384 | 6 | 126851160 | *CENPW* | T | C |  | 0.49 | 0.51 | 0.53 | 0.47 | 0.49 | |  | 0.025 | 0.004 | 2.65E-12 | Ligthart et al. |  |
| rs9385532 | 6 | 130371227 | *L3MBTL3* | T | C |  | 0.67 | 0.69 | 0.68 | 0.68 | 0.67 | |  | 0.026 | 0.004 | 1.90E-11 | Ligthart et al. |  |
| rs1880241 | 7 | 22759469 | *IL6* | G | A |  | 0.52 | 0.52 | 0.51 | 0.51 | 0.51 | |  | 0.028 | 0.004 | 8.41E-14 | Ligthart et al. |  |
| rs2710804 | 7 | 36084529 | *EEPD1* | T | C |  | 0.37 | 0.36 | 0.39 | 0.38 | 0.38 | |  | 0.021 | 0.004 | 1.30E-08 | Ligthart et al. |  |
| rs13233571 | 7 | 72971231 | *BCL7B* | T | C |  | 0.89 | 0.87 | 0.88 | 0.88 | 0.88 | |  | 0.057 | 0.005 | 2.95E-25 | Ligthart et al. |  |
| rs4841132 | 8 | 9183596 | *PPP1R3B* | A | G |  | 0.91 | 0.91 | 0.92 | 0.91 | 0.91 | |  | 0.065 | 0.006 | 2.00E-25 | Ligthart et al. |  |
| rs2064009 | 8 | 117007850 | *TRPS1* | C | T |  | 0.58 | 0.57 | 0.61 | 0.59 | 0.59 | |  | 0.027 | 0.004 | 2.28E-14 | Ligthart et al. |  |
| rs2891677 | 8 | 126344208 | *NSMCE2* | C | T |  | 0.55 | 0.54 | 0.56 | 0.55 | 0.55 | |  | 0.020 | 0.004 | 1.59E-08 | Ligthart et al. |  |
| rs643434 | 9 | 136142355 | *ABO* | G | A |  | 0.36 | 0.37 | 0.33 | 0.35 | 0.35 | |  | 0.023 | 0.004 | 1.02E-09 | Ligthart et al. |  |
| rs1051338 | 10 | 91007360 | *LIPA* | T | G |  | 0.30 | 0.30 | 0.29 | 0.30 | 0.29 | |  | 0.024 | 0.004 | 2.27E-09 | Ligthart et al. |  |
| rs10832027 | 11 | 13357183 | *ARNTL* | G | A |  | 0.68 | 0.68 | 0.67 | 0.68 | 0.68 | |  | 0.026 | 0.004 | 4.44E-12 | Ligthart et al. |  |
| rs10838687 | 11 | 47312892 | *MADD* | G | T |  | 0.79 | 0.79 | 0.79 | 0.79 | 0.80 | |  | 0.031 | 0.004 | 9.12E-13 | Ligthart et al. |  |
| rs1582763 | 11 | 60021948 | *MS4A4A* | A | G |  | 0.64 | 0.62 | 0.63 | 0.64 | 0.62 | |  | 0.022 | 0.004 | 2.37E-09 | Ligthart et al. |  |
| rs7121935 | 11 | 72496148 | *STARD10* | A | G |  | 0.66 | 0.63 | 0.62 | 0.62 | 0.62 | |  | 0.022 | 0.004 | 5.28E-09 | Ligthart et al. |  |
| rs11108056 | 12 | 95855385 | *METAP2* | G | C |  | 0.56 | 0.57 | 0.56 | 0.54 | 0.57 | |  | 0.028 | 0.004 | 5.42E-14 | Ligthart et al. |  |
| rs10778215 | 12 | 103537266 | *C12orf42* | A | T |  | 0.53 | 0.53 | 0.56 | 0.52 | 0.53 | |  | 0.033 | 0.004 | 1.86E-20 | Ligthart et al. |  |
| rs7310409 | 12 | 121424861 | *HNF1A* | A | G |  | 0.62 | 0.61 | 0.61 | 0.63 | 0.61 | |  | 0.137 | 0.004 | 2.54E-299 | Ligthart et al. |  |
| rs2239222 | 14 | 73011885 | *RGS6* | A | G |  | 0.36 | 0.36 | 0.34 | 0.36 | 0.35 | |  | 0.035 | 0.004 | 9.87E-20 | Ligthart et al. |  |
| rs112635299 | 14 | 94838142 | *SERPINA1/SERPINA2P* | T | G |  | 0.98 | 0.98 | 0.98 | 0.98 | 0.98 | |  | 0.107 | 0.017 | 2.10E-10 | Ligthart et al. |  |

Table 1 (Continued)

|  |  |  |  | **Alt allele frequency** | | | | | | | | |  |  |  |  |  |  |
| --- | --- | --- | --- | --- | --- | --- | --- | --- | --- | --- | --- | --- | --- | --- | --- | --- | --- | --- |
|  |  |  |  | **Allele*** | |  | **AS264** | **GARNET** | **GECCO** | **HIPFX** | | **WHIMS** |  |  |  |  |  |  |
| **SNP&** | **Chr** | **Position**¥ | **Gene** | **Ref** | **Alt** |  | **n=1,603** | **n=2,382** | **n=1,393** | **n=1,909** | **n=3,511** | |  | **OR/beta** | **SE** | **p** | **Q / Study (19-22)** |  |
|  | | | | | | | | | | | | | | | | | | |
| (Cont.) GWASs analyzing CRP as a continuous variable that was naturally log-transformed (mg/L) | | | | | | | | | | | | | | | | | |  |
|  | | | | | | | | | | | | | | | | | | |
| rs4774590 | 15 | 51745277 | *DMXL2* | A | G |  | 0.62 | 0.63 | 0.63 | 0.62 | 0.61 | |  | 0.022 | 0.004 | 2.71E-08 | Ligthart et al. |  |
| rs1189402 | 15 | 53728154 | *WDR72* | G | A |  | 0.63 | 0.63 | 0.62 | 0.64 | 0.62 | |  | 0.025 | 0.004 | 3.90E-09 | Ligthart et al. |  |
| rs340005 | 15 | 60878030 | *RORA* | G | A |  | 0.62 | 0.63 | 0.63 | 0.61 | 0.62 | |  | 0.030 | 0.004 | 1.01E-15 | Ligthart et al. |  |
| rs10521222 | 16 | 51158710 | *SALL1* | T | C |  | NA | 0.96 | 0.96 | 0.95 | NA | |  | 0.104 | 0.011 | 2.06E-22 | Ligthart et al. |  |
| rs1558902 | 16 | 53803574 | *FTO* | T | A |  | 0.39 | 0.41 | 0.41 | 0.40 | 0.40 | |  | 0.034 | 0.004 | 5.20E-20 | Ligthart et al. |  |
| rs178810 | 17 | 16097430 | *NCOR1* | C | T |  | NA | 0.56 | 0.57 | 0.57 | 0.56 | |  | 0.020 | 0.004 | 2.95E-08 | Ligthart et al. |  |
| rs10512597 | 17 | 72699833 | *CD300LF/RAB37* | T | C |  | 0.81 | 0.81 | 0.83 | 0.82 | 0.81 | |  | 0.037 | 0.005 | 4.44E-14 | Ligthart et al. |  |
| rs2852151 | 18 | 12841176 | *PTPN2* | G | A |  | 0.39 | 0.39 | 0.39 | 0.40 | 0.40 | |  | 0.025 | 0.004 | 1.36E-11 | Ligthart et al. |  |
| rs4092465 | 18 | 55080437 | *ONECUT2* | A | G |  | 0.62 | 0.63 | NA | 0.65 | 0.64 | |  | 0.027 | 0.004 | 3.11E-10 | Ligthart et al. |  |
| rs12960928 | 18 | 57897803 | *MC4R* | T | C |  | 0.26 | 0.26 | 0.26 | 0.27 | 0.27 | |  | 0.024 | 0.004 | 1.91E-09 | Ligthart et al. |  |
| rs4420638 | 19 | 45422946 | *APOC1* | G | A |  | 0.83 | NA | NA | NA | 0.83 | |  | 0.229 | 0.006 | 1.23E-305 | Ligthart et al. |  |
| rs1800961 | 20 | 43042364 | *HNF4A* | T | C |  | 0.97 | 0.97 | 0.96 | 0.97 | 0.97 | |  | 0.112 | 0.011 | 4.63E-23 | Ligthart et al. |  |
| rs2315008 | 20 | 62343956 | *ZGPAT* | T | G |  | 0.69 | 0.68 | 0.69 | 0.69 | 0.67 | |  | 0.023 | 0.004 | 5.36E-10 | Ligthart et al. |  |
| rs2836878 | 21 | 40465534 | *PSMG1* | A | G |  | 0.73 | 0.73 | 0.74 | 0.73 | 0.73 | |  | 0.043 | 0.004 | 7.71E-26 | Ligthart et al. |  |
| rs6001193 | 22 | 39074737 | *TOMM22* | G | A |  | NA | 0.65 | 0.67 | 0.64 | 0.64 | |  | 0.028 | 0.004 | 6.53E-14 | Ligthart et al. |  |

Alt, alternative; Chr, chromosome; CI, confidence interval; CRP, C-reactive protein; GWAS, genome-wide association study; LD, linkage disequilibrium; OR, odds ratio; Q, Cochran’s Q test; Ref, reference; SNP, single-nucleotide polymorphism.

& Among SNPs with LD ≥ 0.3, the SNPs with the lowest p value were selected.

¥ GRCh37 coordinated.

* The allele having a positive effect was assigned to an alternative allele.

† The effect estimate of the CRP SNP was exponentiated, reflecting the OR predicting chronic inflammation status (CRP > 3.0 mg/L compared with ≤ 3.0 mg/L)
