# Supplementary material for: Genetically Predicted C-Reactive Protein Associated With Postmenopausal Breast Cancer Risk: Interrelation With Estrogen and Cancer Molecular Subtypes Using Mendelian Randomization
Source: Front Oncol. 2021 Feb 3;10:630994. doi: 10.3389/fonc.2020.630994 (PMC7888276; doi:10.3389/fonc.2020.630994)
Supplement: Supplementary file 1 [file DataSheet_1.zip › TableS2_2020Nov18.docx]

Table S2. Pleiotropic genome-wide SNPs that are associated with obesity (BMI and WHR), diabetic syndromes and diabetes (FG, FI, post 2-hr glucose, and T2DM), and lipid (HDL, LDL, total cholesterol, and triglycerides) in previous studies (BMI, body mass index; FG, fasting glucose; FI, fasting insulin; HDL, high-density lipoprotein; LD, linkage disequilibrium; LDL, low-density lipoprotein; SE, standard error; SNP, single-nucleotide polymorphism; T2DM, type 2 diabetes; WHR, waist-to-hip ratio.)

1. **Shu et al. (16)**

| **SNPs** | **Traits** | **SNPs overlapped/in LD between traits** | **Effect allele** | **β** |
| --- | --- | --- | --- | --- |
| rs1019503 | 2hrGlu |  | A | 0.063 |
| rs10423928 | 2hrGlu | BMI, rs1800437, R^2^=1 | A | 0.13 |
| rs11782386 | 2hrGlu |  | C | 0.099 |
| rs12255372 | 2hrGlu |  | T | 0.092 |
| rs1260326 | 2hrGlu | FI, FG: rs780094, R^2^=0.84 | T | 0.059 |
| rs1436958 | 2hrGlu |  | T | 0.064 |
| rs2877716 | 2hrGlu | FG: rs11708067, R^2^=0.84 | C | 0.081 |
| rs6975024 | 2hrGlu | FG: rs4607517, R^2^=0.99 | C | 0.103 |
| rs7651090 | 2hrGlu | FG: rs7651090 | G | 0.064 |
| rs10747083 | FG |  | A | 0.013 |
| rs10811661 | FG |  | T | 0.024 |
| rs10830963 | FG |  | G | 0.078 |
| rs10885122 | FG |  | G | 0.027 |
| rs11071657 | FG |  | A | 0.008 |
| rs11558471 | FG |  | A | 0.029 |
| rs11603334 | FG |  | G | 0.019 |
| rs11605924 | FG |  | A | 0.02 |
| rs11619319 | FG |  | G | 0.02 |
| rs11708067 | FG | 2hrGlu: rs2877716, R^2^=0.84 | A | 0.023 |
| rs11715915 | FG |  | C | 0.012 |
| rs11920090 | FG |  | T | 0.026 |
| rs16913693 | FG |  | T | 0.043 |
| rs174550 | FG |  | T | 0.019 |
| rs17762454 | FG |  | T | 0.014 |
| rs2191349 | FG |  | T | 0.029 |
| rs2302593 | FG |  | C | 0.014 |
| rs2657879 | FG |  | G | 0.016 |
| rs340874 | FG |  | C | 0.014 |
| rs3783347 | FG |  | G | 0.017 |
| rs3829109 | FG |  | G | 0.017 |
| rs4506565 | FG | BMI: rs7903146, R^2^: 0.88; FI: rs7903146, R^2^: 0.88 | T | 0.021 |
| rs4607517 | FG | 2hrGlu: rs6975024, R^2^=0.99 | A | 0.058 |
| rs4869272 | FG |  | T | 0.018 |
| rs560887 | FG |  | C | 0.071 |
| rs576674 | FG |  | G | 0.017 |
| rs6072275 | FG |  | A | 0.016 |
| rs6113722 | FG |  | G | 0.035 |
| rs6943153 | FG |  | T | 0.015 |
| rs7034200 | FG |  | A | 0.018 |
| rs7651090 | FG | 2hrGlu: rs7651090 | G | 0.013 |
| rs7708285 | FG |  | G | 0.015 |
| rs780094 | FG | FI: rs780094; 2hrGlu: rs1260326, R^2^=0.84 | C | 0.027 |
| rs7944584 | FG |  | A | 0.023 |
| rs9368222 | FG |  | A | 0.014 |
| rs983309 | FG |  | T | 0.026 |
| rs10195252 | FI | WHR_adj BMI_: rs10195252 | T | 0.016 |
| rs1167800 | FI |  | A | 0.016 |
| rs1530559 | FI |  | A | 0.015 |
| rs17036328 | FI | BMI: rs1801282, R^2^=0.99 | T | 0.021 |
| rs2126259 | FI |  | T | 0.024 |
| rs2745353 | FI | WHR_adj BMI_: rs1936805, R^2^=1 | T | 0.014 |
| rs2943645 | FI | BMI: rs2176040, R2=0.99 | T | 0.019 |
| rs3822072 | FI | WHR_adj BMI_: rs9991328, R^2^=0.96 | A | 0.012 |
| rs459193 | FI | WHR_adj BMI_: rs459193 | G | 0.015 |
| rs4846565 | FI |  | G | 0.013 |
| rs4865796 | FI |  | A | 0.015 |
| rs6822892 | FI |  | A | 0.014 |
| rs6912327 | FI |  | T | 0.017 |
| rs731839 | FI |  | G | 0.015 |
| rs780094 | FI | FG: rs780094, 2hrGlu: rs1260326, R^2^=0.84 | C | 0.029 |
| rs7903146 | FI | BMI: rs7903146; FG: rs4506565, R^2^=0.88 | C | 0.018 |
| rs860598 | FI |  | A | 0.018 |
| rs974801 | FI |  | G | 0.014 |
| rs1000940 | BMI |  | G | 0.019 |
| rs10132280 | BMI |  | C | 0.023 |
| rs1045706 | BMI |  | C | 0.011 |
| rs1049205 | BMI |  | C | 0.012 |
| rs1051695 | BMI |  | A | 0.016 |
| rs1052618 | BMI |  | G | 0.011 |
| rs1053874 | BMI |  | A | 0.012 |
| rs1062633 | BMI |  | C | 0.019 |
| rs10733682 | BMI |  | A | 0.017 |
| rs10938397 | BMI |  | G | 0.04 |
| rs10968576 | BMI |  | G | 0.025 |
| rs1105223 | BMI |  | T | 0.011 |
| rs11057405 | BMI |  | G | 0.031 |
| rs11071896 | BMI |  | A | 0.013 |
| rs11189513 | BMI |  | A | 0.014 |
| rs1126930 | BMI |  | C | 0.034 |
| rs1131877 | BMI |  | C | 0.017 |
| rs1136001 | BMI |  | G | 0.011 |
| rs11539157 | BMI |  | C | 0.014 |
| rs11546878 | BMI |  | C | 0.017 |
| rs11554159 | BMI |  | A | 0.015 |
| rs11555762 | BMI |  | T | 0.014 |
| rs11583200 | BMI |  | C | 0.018 |
| rs11676272 | BMI |  | G | 0.029 |
| rs1167827 | BMI |  | G | 0.02 |
| rs11688816 | BMI |  | G | 0.017 |
| rs1169081 | BMI |  | G | 0.012 |
| rs11755393 | BMI |  | G | 0.02 |
| rs11847697 | BMI |  | T | 0.049 |
| rs12140153 | BMI |  | G | 0.03 |
| rs12199003 | BMI |  | T | 0.011 |
| rs12236219 | BMI |  | C | 0.025 |
| rs12273892 | BMI |  | A | 0.015 |
| rs12711521 | BMI |  | C | 0.013 |
| rs12828016 | BMI |  | G | 0.014 |
| rs12885454 | BMI |  | C | 0.021 |
| rs13078807 | BMI |  | G | 0.03 |
| rs13107325 | BMI |  | T | 0.048 |
| rs13191362 | BMI |  | A | 0.028 |
| rs13303 | BMI |  | C | 0.011 |
| rs141845046 | BMI |  | T | 0.048 |
| rs1441264 | BMI |  | A | 0.018 |
| rs145878042 | BMI |  | G | 0.066 |
| rs1528435 | BMI |  | T | 0.018 |
| rs1550116 | BMI |  | G | 0.016 |
| rs1555543 | BMI |  | C | 0.022 |
| rs1558902 | BMI |  | A | 0.082 |
| rs15818 | BMI |  | G | 0.013 |
| rs16849342 | BMI |  | C | 0.02 |
| rs16851483 | BMI |  | T | 0.048 |
| rs16907751 | BMI |  | C | 0.047 |
| rs17001654 | BMI |  | G | 0.031 |
| rs17024393 | BMI |  | C | 0.066 |
| rs17094222 | BMI |  | C | 0.025 |
| rs17203016 | BMI |  | G | 0.021 |
| rs17405819 | BMI |  | T | 0.022 |
| rs17724992 | BMI |  | A | 0.019 |
| rs17751061 | BMI |  | C | 0.021 |
| rs1800437 | BMI | 2hrGlu: rs10423928, R^2^=1 | G | 0.031 |
| rs1801265 | BMI |  | G | 0.013 |
| rs1801282 | BMI | FI: rs17036328, R^2^=0.99 | G | 0.024 |
| rs1805123 | BMI |  | T | 0.017 |
| rs1808579 | BMI |  | C | 0.017 |
| rs1885987 | BMI |  | T | 0.01 |
| rs1928295 | BMI |  | T | 0.019 |
| rs1933437 | BMI |  | G | 0.016 |
| rs197412 | BMI |  | C | 0.013 |
| rs203462 | BMI |  | T | 0.01 |
| rs2075650 | BMI |  | A | 0.026 |
| rs2075803 | BMI |  | G | 0.01 |
| rs2076559 | BMI |  | A | 0.016 |
| rs2080454 | BMI |  | C | 0.017 |
| rs2111119 | BMI |  | C | 0.02 |
| rs215607 | BMI |  | G | 0.013 |
| rs2176040 | BMI | FI: rs2943645, R^2^=0.99 | A | 0.014 |
| rs2178403 | BMI |  | A | 0.014 |
| rs2228210 | BMI |  | A | 0.014 |
| rs2228273 | BMI |  | A | 0.024 |
| rs2228552 | BMI |  | T | 0.012 |
| rs2241423 | BMI |  | G | 0.031 |
| rs2274741 | BMI |  | T | 0.016 |
| rs2277598 | BMI |  | C | 0.015 |
| rs2280843 | BMI |  | G | 0.015 |
| rs2296172 | BMI |  | G | 0.015 |
| rs2297792 | BMI |  | C | 0.013 |
| rs2301680 | BMI |  | G | 0.014 |
| rs2306590 | BMI |  | G | 0.016 |
| rs2307111 | BMI |  | T | 0.023 |
| rs2396359 | BMI |  | T | 0.015 |
| rs2815752 | BMI |  | A | 0.033 |
| rs2820292 | BMI |  | C | 0.02 |
| rs2836754 | BMI |  | C | 0.016 |
| rs284860 | BMI |  | T | 0.01 |
| rs2867125 | BMI |  | C | 0.06 |
| rs2890652 | BMI |  | C | 0.025 |
| rs29941 | BMI |  | G | 0.018 |
| rs30187 | BMI |  | C | 0.01 |
| rs3088142 | BMI |  | T | 0.017 |
| rs3184504 | BMI |  | C | 0.013 |
| rs34149579 | BMI |  | G | 0.033 |
| rs34811474 | BMI |  | G | 0.024 |
| rs3732530 | BMI |  | C | 0.015 |
| rs3736485 | BMI |  | A | 0.018 |
| rs3749591 | BMI |  | G | 0.012 |
| rs3760128 | BMI |  | G | 0.013 |
| rs3765407 | BMI |  | G | 0.015 |
| rs3766160 | BMI |  | G | 0.012 |
| rs3781409 | BMI |  | T | 0.011 |
| rs3810291 | BMI |  | A | 0.028 |
| rs3817334 | BMI |  | T | 0.026 |
| rs3849570 | BMI |  | A | 0.019 |
| rs4077410 | BMI |  | G | 0.017 |
| rs41312309 | BMI |  | T | 0.021 |
| rs45465594 | BMI |  | A | 0.04 |
| rs455527 | BMI |  | T | 0.02 |
| rs459552 | BMI |  | T | 0.012 |
| rs4741510 | BMI |  | T | 0.012 |
| rs4771122 | BMI |  | G | 0.03 |
| rs4851287 | BMI |  | A | 0.013 |
| rs4889891 | BMI |  | C | 0.012 |
| rs492400 | BMI |  | C | 0.016 |
| rs5219 | BMI |  | C | 0.012 |
| rs540742 | BMI |  | T | 0.012 |
| rs543874 | BMI |  | G | 0.048 |
| rs56084453 | BMI |  | A | 0.012 |
| rs56214831 | BMI |  | T | 0.057 |
| rs56384862 | BMI |  | G | 0.013 |
| rs571312 | BMI |  | A | 0.056 |
| rs5758651 | BMI |  | T | 0.012 |
| rs6050446 | BMI |  | G | 0.034 |
| rs6065 | BMI |  | T | 0.024 |
| rs61747555 | BMI |  | A | 0.015 |
| rs61754230 | BMI |  | T | 0.04 |
| rs62051555 | BMI |  | C | 0.024 |
| rs6234 | BMI |  | C | 0.017 |
| rs6265 | BMI |  | C | 0.041 |
| rs6477694 | BMI |  | C | 0.017 |
| rs657452 | BMI |  | A | 0.023 |
| rs6893216 | BMI |  | T | 0.018 |
| rs7126805 | BMI |  | A | 0.011 |
| rs7138803 | BMI |  | A | 0.032 |
| rs7191155 | BMI |  | T | 0.017 |
| rs7239883 | BMI |  | G | 0.023 |
| rs7243357 | BMI |  | T | 0.022 |
| rs7359397 | BMI |  | T | 0.031 |
| rs7406910 | BMI |  | C | 0.018 |
| rs7599312 | BMI |  | G | 0.022 |
| rs7601000 | BMI |  | T | 0.012 |
| rs7636 | BMI |  | A | 0.023 |
| rs7653652 | BMI |  | C | 0.015 |
| rs7715256 | BMI |  | G | 0.016 |
| rs78648104 | BMI |  | T | 0.02 |
| rs7899106 | BMI |  | G | 0.04 |
| rs7903146 | BMI | FG: rs4506565, R^2^=0.88; FI: rs7903146 | C | 0.023 |
| rs7978353 | BMI |  | A | 0.01 |
| rs80317617 | BMI |  | C | 0.019 |
| rs861539 | BMI |  | G | 0.013 |
| rs887912 | BMI |  | T | 0.023 |
| rs9374842 | BMI |  | T | 0.023 |
| rs9400239 | BMI |  | C | 0.019 |
| rs9438 | BMI |  | C | 0.013 |
| rs9652588 | BMI |  | C | 0.013 |
| rs977747 | BMI |  | T | 0.017 |
| rs987237 | BMI |  | G | 0.045 |
| rs9891146 | BMI |  | T | 0.015 |
| rs9925964 | BMI |  | A | 0.019 |
| rs10195252 | WHR_adj BMI_ | FI: rs10195252 | T | 0.027 |
| rs10245353 | WHR_adj BMI_ |  | A | 0.035 |
| rs1045241 | WHR_adj BMI_ |  | C | 0.035 |
| rs10783615 | WHR_adj BMI_ |  | G | 0.036 |
| rs10804591 | WHR_adj BMI_ |  | A | 0.025 |
| rs10842707 | WHR_adj BMI_ |  | T | 0.032 |
| rs10919388 | WHR_adj BMI_ |  | C | 0.024 |
| rs10991437 | WHR_adj BMI_ |  | A | 0.031 |
| rs11231693 | WHR_adj BMI_ |  | A | 0.041 |
| rs12143789 | WHR_adj BMI_ |  | C | 0.024 |
| rs12454712 | WHR_adj BMI_ |  | T | 0.035 |
| rs12608504 | WHR_adj BMI_ |  | A | 0.022 |
| rs12679556 | WHR_adj BMI_ |  | G | 0.027 |
| rs1294410 | WHR_adj BMI_ |  | C | 0.031 |
| rs1385167 | WHR_adj BMI_ |  | G | 0.029 |
| rs1440372 | WHR_adj BMI_ |  | C | 0.024 |
| rs1534696 | WHR_adj BMI_ |  | C | 0.027 |
| rs1569135 | WHR_adj BMI_ |  | A | 0.021 |
| rs17451107 | WHR_adj BMI_ |  | T | 0.026 |
| rs1776897 | WHR_adj BMI_ |  | G | 0.052 |
| rs17819328 | WHR_adj BMI_ |  | G | 0.021 |
| rs1936805 | WHR_adj BMI_ | FI: rs2745353, R^2^=1 | T | 0.043 |
| rs2071449 | WHR_adj BMI_ |  | A | 0.028 |
| rs224333 | WHR_adj BMI_ |  | G | 0.02 |
| rs2276824 | WHR_adj BMI_ |  | C | 0.024 |
| rs2294239 | WHR_adj BMI_ |  | A | 0.025 |
| rs2371767 | WHR_adj BMI_ |  | G | 0.036 |
| rs2645294 | WHR_adj BMI_ |  | T | 0.031 |
| rs2820443 | WHR_adj BMI_ |  | T | 0.035 |
| rs2925979 | WHR_adj BMI_ |  | T | 0.032 |
| rs303084 | WHR_adj BMI_ |  | A | 0.023 |
| rs3805389 | WHR_adj BMI_ |  | A | 0.027 |
| rs4081724 | WHR_adj BMI_ |  | G | 0.035 |
| rs459193 | WHR_adj BMI_ | FI: rs459193 | A | 0.026 |
| rs4646404 | WHR_adj BMI_ |  | G | 0.027 |
| rs4765219 | WHR_adj BMI_ |  | C | 0.028 |
| rs6090583 | WHR_adj BMI_ |  | A | 0.022 |
| rs6556301 | WHR_adj BMI_ |  | T | 0.022 |
| rs714515 | WHR_adj BMI_ |  | G | 0.027 |
| rs72959041 | WHR_adj BMI_ |  | A | 0.125 |
| rs7705502 | WHR_adj BMI_ |  | A | 0.027 |
| rs7759742 | WHR_adj BMI_ |  | A | 0.023 |
| rs7801581 | WHR_adj BMI_ |  | T | 0.027 |
| rs7830933 | WHR_adj BMI_ |  | A | 0.037 |
| rs7917772 | WHR_adj BMI_ |  | A | 0.027 |
| rs8030605 | WHR_adj BMI_ |  | A | 0.03 |
| rs8042543 | WHR_adj BMI_ |  | C | 0.026 |
| rs8066985 | WHR_adj BMI_ |  | A | 0.026 |
| rs863750 | WHR_adj BMI_ |  | T | 0.026 |
| rs905938 | WHR_adj BMI_ |  | T | 0.025 |
| rs9687846 | WHR_adj BMI_ |  | A | 0.041 |
| rs979012 | WHR_adj BMI_ |  | T | 0.027 |
| rs998584 | WHR_adj BMI_ |  | A | 0.043 |
| rs9991328 | WHR_adj BMI_ | FI: rs3822072, R^2^=0.96 | T | 0.019 |

1. **Carreras-Torres et al. (44)**
   1. **BMI**

| **SNP** | **Chr** | **Position** | **Mean imputation quality** | **Effect allele** | **Other allele** | **Estimate** | **SE** | **Trait** |
| --- | --- | --- | --- | --- | --- | --- | --- | --- |
| rs1000940 | 17 | 5283252 | 1 | G | A | 0.02 | 0 | BMI |
| rs10132280 | 14 | 25928179 | 0.99 | C | A | 0.02 | 0 | BMI |
| rs1016287 | 2 | 59305625 | 1 | T | C | 0.02 | 0 | BMI |
| rs10182181 | 2 | 25150296 | 1 | G | A | 0.03 | 0 | BMI |
| rs10464483 | 7 | 93193242 | 1 | T | C | 0.02 | 0 | BMI |
| rs10733682 | 9 | 129460914 | 1 | A | G | 0.02 | 0 | BMI |
| rs10938397 | 4 | 45182527 | 1 | G | A | 0.04 | 0 | BMI |
| rs10968576 | 9 | 28414339 | 1 | G | A | 0.03 | 0 | BMI |
| rs11030104 | 11 | 27684517 | 1 | A | G | 0.04 | 0 | BMI |
| rs11057405 | 12 | 122781897 | 1 | G | A | 0.03 | 0.01 | BMI |
| rs11126666 | 2 | 26928811 | 1 | A | G | 0.02 | 0 | BMI |
| rs11165643 | 1 | 96924097 | 1 | T | C | 0.02 | 0 | BMI |
| rs11191560 | 10 | 104869038 | 1 | C | T | 0.03 | 0.01 | BMI |
| rs11583200 | 1 | 50559820 | 1 | C | T | 0.02 | 0 | BMI |
| rs1167827 | 7 | 75163169 | 1 | G | A | 0.02 | 0 | BMI |
| rs11688816 | 2 | 63053048 | 1 | G | A | 0.02 | 0 | BMI |
| rs11727676 | 4 | 145659064 | 0.84 | T | C | 0.04 | 0.01 | BMI |
| rs11847697 | 14 | 30515112 | 0.98 | T | C | 0.05 | 0.01 | BMI |
| rs12286929 | 11 | 115022404 | 0.99 | G | A | 0.02 | 0 | BMI |
| rs12401738 | 1 | 78446761 | 0.99 | A | G | 0.02 | 0 | BMI |
| rs12429545 | 13 | 54102206 | 1 | A | G | 0.03 | 0.01 | BMI |
| rs12446632 | 16 | 19935389 | 1 | G | A | 0.04 | 0.01 | BMI |
| rs12566985 | 1 | 75002193 | 1 | G | A | 0.02 | 0 | BMI |
| rs12885454 | 14 | 29736838 | 0.99 | C | A | 0.02 | 0 | BMI |
| rs12940622 | 17 | 78615571 | 1 | G | A | 0.02 | 0 | BMI |
| rs13021737 | 2 | 632348 | 0.99 | G | A | 0.06 | 0 | BMI |
| rs13078960 | 3 | 85807590 | 1 | G | T | 0.03 | 0 | BMI |
| rs13107325 | 4 | 103188709 | 0.99 | T | C | 0.05 | 0.01 | BMI |
| rs13191362 | 6 | 163033350 | 0.99 | A | G | 0.03 | 0.01 | BMI |
| rs13201877 | 6 | 137675541 | 0.94 | G | A | 0.02 | 0.01 | BMI |
| rs1421085 | 16 | 53800954 | 1 | C | T | 0.08 | 0 | BMI |
| rs1441264 | 13 | 79580919 | 1 | A | G | 0.02 | 0 | BMI |
| rs1460676 | 2 | 164567689 | 0.99 | C | T | 0.02 | 0 | BMI |
| rs1516725 | 3 | 185824004 | 0.99 | C | T | 0.05 | 0.01 | BMI |
| rs1528435 | 2 | 181550962 | 1 | T | C | 0.02 | 0 | BMI |
| rs16851483 | 3 | 141275436 | 1 | T | G | 0.05 | 0.01 | BMI |
| rs16907751 | 8 | 81375457 | 0.98 | C | T | 0.04 | 0.01 | BMI |
| rs16951275 | 15 | 68077168 | 1 | T | C | 0.03 | 0 | BMI |
| rs17024393 | 1 | 110154688 | 0.99 | C | T | 0.07 | 0.01 | BMI |
| rs17094222 | 10 | 102395440 | 0.98 | C | T | 0.03 | 0 | BMI |
| rs17203016 | 2 | 208255518 | 1 | G | A | 0.02 | 0 | BMI |
| rs17405819 | 8 | 76806584 | 1 | T | C | 0.02 | 0 | BMI |
| rs17724992 | 19 | 18454825 | 1 | A | G | 0.02 | 0 | BMI |
| rs1808579 | 18 | 21104888 | 1 | C | T | 0.02 | 0 | BMI |
| rs1928295 | 9 | 120378483 | 1 | T | C | 0.02 | 0 | BMI |
| rs2033529 | 6 | 40348653 | 1 | G | A | 0.02 | 0 | BMI |
| rs2033732 | 8 | 85079709 | 0.99 | C | T | 0.02 | 0 | BMI |
| rs205262 | 6 | 34563164 | 1 | G | A | 0.02 | 0 | BMI |
| rs2075650 | 19 | 45395619 | 1 | A | G | 0.03 | 0.01 | BMI |
| rs2080454 | 16 | 49062590 | 1 | C | A | 0.02 | 0 | BMI |
| rs2112347 | 5 | 75015242 | 0.99 | T | G | 0.03 | 0 | BMI |
| rs2121279 | 2 | 143043285 | 1 | T | C | 0.03 | 0 | BMI |
| rs2176040 | 2 | 227092802 | 1 | A | G | 0.01 | 0 | BMI |
| rs2176598 | 11 | 43864278 | 1 | T | C | 0.02 | 0 | BMI |
| rs2207139 | 6 | 50845490 | 1 | G | A | 0.05 | 0 | BMI |
| rs2245368 | 7 | 76608143 | 0.94 | C | T | 0.03 | 0.01 | BMI |
| rs2287019 | 19 | 46202172 | 1 | C | T | 0.04 | 0 | BMI |
| rs2316901 | 11 | 8679016 | 1 | G | A | 0.02 | 0 | BMI |
| rs2365389 | 3 | 61236462 | 0.99 | C | T | 0.02 | 0 | BMI |
| rs2650492 | 16 | 28333411 | 1 | A | G | 0.02 | 0 | BMI |
| rs2820292 | 1 | 201784287 | 0.99 | C | A | 0.02 | 0 | BMI |
| rs2836754 | 21 | 40291740 | 1 | C | T | 0.02 | 0 | BMI |
| rs29941 | 19 | 34309532 | 1 | G | A | 0.02 | 0 | BMI |
| rs3101336 | 1 | 72751185 | 1 | C | T | 0.03 | 0 | BMI |
| rs3736485 | 15 | 51748610 | 0.99 | A | G | 0.02 | 0 | BMI |
| rs3810291 | 19 | 47569003 | 0.91 | A | G | 0.03 | 0 | BMI |
| rs3817334 | 11 | 47650993 | 1 | T | C | 0.03 | 0 | BMI |
| rs3849570 | 3 | 81792112 | 1 | A | C | 0.02 | 0 | BMI |
| rs3888190 | 16 | 28889486 | 1 | A | C | 0.03 | 0 | BMI |
| rs4740619 | 9 | 15634326 | 1 | T | C | 0.02 | 0 | BMI |
| rs4787491 | 16 | 30015337 | 0.99 | G | A | 0.02 | 0 | BMI |
| rs492400 | 2 | 219349752 | 0.99 | C | T | 0.02 | 0 | BMI |
| rs543874 | 1 | 177889480 | 1 | G | A | 0.05 | 0 | BMI |
| rs6091540 | 20 | 51087862 | 1 | C | T | 0.02 | 0 | BMI |
| rs6465468 | 7 | 95169514 | 0.98 | T | G | 0.02 | 0 | BMI |
| rs6477694 | 9 | 111932342 | 1 | C | T | 0.02 | 0 | BMI |
| rs6567160 | 18 | 57829135 | 0.99 | C | T | 0.06 | 0 | BMI |
| rs657452 | 1 | 49589847 | 1 | A | G | 0.02 | 0 | BMI |
| rs6804842 | 3 | 25106437 | 1 | G | A | 0.02 | 0 | BMI |
| rs7138803 | 12 | 50247468 | 1 | A | G | 0.03 | 0 | BMI |
| rs7141420 | 14 | 79899454 | 1 | T | C | 0.02 | 0 | BMI |
| rs7164727 | 15 | 73093991 | 1 | T | C | 0.02 | 0 | BMI |
| rs7239883 | 18 | 40147671 | 1 | G | A | 0.02 | 0 | BMI |
| rs7243357 | 18 | 56883319 | 0.99 | T | G | 0.02 | 0 | BMI |
| rs7599312 | 2 | 213413231 | 1 | G | A | 0.02 | 0 | BMI |
| rs7715256 | 5 | 153537893 | 1 | G | T | 0.02 | 0 | BMI |
| rs7899106 | 10 | 87410904 | 1 | G | A | 0.04 | 0.01 | BMI |
| rs7903146 | 10 | 114758349 | 1 | C | T | 0.02 | 0 | BMI |
| rs8082647 | 17 | 2010597 | 1 | C | T | 0.01 | 0 | BMI |
| rs9374842 | 6 | 120185665 | 1 | T | C | 0.02 | 0 | BMI |
| rs9400239 | 6 | 108977663 | 0.99 | C | T | 0.02 | 0 | BMI |
| rs9540493 | 13 | 66205704 | 0.98 | A | G | 0.02 | 0 | BMI |
| rs9581854 | 13 | 28017782 | 0.99 | T | C | 0.03 | 0.01 | BMI |
| rs977747 | 1 | 47684677 | 1 | T | G | 0.02 | 0 | BMI |
| rs9925964 | 16 | 31129895 | 1 | A | G | 0.02 | 0 | BMI |

- 1. **WHR**

| **SNP** | **Chr** | **Position** | **Mean imputation quality** | **Effect allele** | **Other allele** | **Estimate** | **SE** | **Trait** |
| --- | --- | --- | --- | --- | --- | --- | --- | --- |
| rs2765539 | 1 | 119549418 | 1 | T | C | 0.03 | 0 | Waist-to-hip ratio |
| rs1011731 | 1 | 172346548 | 1 | G | A | 0.02 | 0 | Waist-to-hip ratio |
| rs1563355 | 1 | 219653101 | 1 | C | T | 0.03 | 0 | Waist-to-hip ratio |
| rs929641 | 2 | 58792377 | 1 | A | G | 0.02 | 0 | Waist-to-hip ratio |
| rs1128249 | 2 | 165528624 | 0.99 | G | T | 0.02 | 0 | Waist-to-hip ratio |
| rs1569135 | 2 | 188115398 | 1 | A | G | 0.02 | 0 | Waist-to-hip ratio |
| rs2972164 | 3 | 12334416 | 0.99 | C | T | 0.02 | 0 | Waist-to-hip ratio |
| rs904453 | 3 | 12704894 | 1 | T | G | 0.02 | 0 | Waist-to-hip ratio |
| rs9860730 | 3 | 64701146 | 0.99 | A | G | 0.02 | 0 | Waist-to-hip ratio |
| rs17451107 | 3 | 156797609 | 0.98 | T | C | 0.02 | 0 | Waist-to-hip ratio |
| rs459193 | 5 | 55806751 | 0.98 | A | G | 0.03 | 0 | Waist-to-hip ratio |
| rs1294421 | 6 | 6743149 | 0.99 | G | T | 0.03 | 0 | Waist-to-hip ratio |
| rs11755724 | 6 | 7118990 | 1 | G | A | 0.02 | 0 | Waist-to-hip ratio |
| rs998584 | 6 | 43757896 | 0.92 | A | C | 0.03 | 0 | Waist-to-hip ratio |
| rs2207139 | 6 | 50845490 | 1 | G | A | 0.03 | 0 | Waist-to-hip ratio |
| rs2745359 | 6 | 127381956 | 0.9 | C | T | 0.06 | 0.01 | Waist-to-hip ratio |
| rs10245353 | 7 | 25858614 | 0.99 | A | C | 0.03 | 0 | Waist-to-hip ratio |
| rs7801581 | 7 | 27223771 | 0.99 | T | C | 0.02 | 0 | Waist-to-hip ratio |
| rs12549058 | 8 | 72492238 | 1 | G | T | 0.04 | 0.01 | Waist-to-hip ratio |
| rs4929927 | 11 | 8658485 | 1 | G | A | 0.02 | 0 | Waist-to-hip ratio |
| rs11048470 | 12 | 26487283 | 1 | T | G | 0.03 | 0 | Waist-to-hip ratio |
| rs10783615 | 12 | 54349773 | 0.95 | G | A | 0.04 | 0 | Waist-to-hip ratio |
| rs10876528 | 12 | 54421476 | 0.99 | A | C | 0.03 | 0 | Waist-to-hip ratio |
| rs1316952 | 12 | 124399550 | 1 | T | C | 0.03 | 0 | Waist-to-hip ratio |
| rs17109256 | 14 | 79939993 | 1 | A | G | 0.02 | 0 | Waist-to-hip ratio |
| rs1440372 | 15 | 67033151 | 0.99 | C | T | 0.02 | 0 | Waist-to-hip ratio |
| rs1121980 | 16 | 53809247 | 1 | A | G | 0.04 | 0 | Waist-to-hip ratio |
| rs4640244 | 17 | 21284223 | 1 | G | A | 0.02 | 0 | Waist-to-hip ratio |
| rs11663816 | 18 | 57876227 | 1 | C | T | 0.03 | 0 | Waist-to-hip ratio |
| rs3786897 | 19 | 33893008 | 0.99 | G | A | 0.02 | 0 | Waist-to-hip ratio |
| rs2075650 | 19 | 45395619 | 1 | A | G | 0.03 | 0 | Waist-to-hip ratio |
| rs2287019 | 19 | 46202172 | 1 | C | T | 0.03 | 0 | Waist-to-hip ratio |
| rs16996700 | 20 | 50981945 | 1 | T | C | 0.02 | 0 | Waist-to-hip ratio |
| rs2179129 | 22 | 29450923 | 1 | A | G | 0.02 | 0 | Waist-to-hip ratio |

- 1. **FG**

| **SNP** | **Chr** | **Position** | **Mean imputation quality** | **Effect allele** | **Other allele** | **Estimate** | **SE** | **Trait** |
| --- | --- | --- | --- | --- | --- | --- | --- | --- |
| rs340874 | 1 | 214159256 | 1 | C | T | 0.02 | 0 | Fasting glucose |
| rs780094 | 2 | 27741237 | 1 | C | T | 0.04 | 0 | Fasting glucose |
| rs560887 | 2 | 169763148 | 1 | C | T | 0.09 | 0 | Fasting glucose |
| rs11715915 | 3 | 49455330 | 1 | C | T | 0.02 | 0 | Fasting glucose |
| rs11708067 | 3 | 123065778 | 0.99 | A | G | 0.03 | 0 | Fasting glucose |
| rs1280 | 3 | 170713290 | 1 | T | C | 0.03 | 0 | Fasting glucose |
| rs7651090 | 3 | 185513392 | 1 | G | A | 0.02 | 0 | Fasting glucose |
| rs7708285 | 5 | 76425867 | 1 | G | A | 0.02 | 0 | Fasting glucose |
| rs4869272 | 5 | 95539448 | 1 | T | C | 0.02 | 0 | Fasting glucose |
| rs17762454 | 6 | 7213200 | 1 | T | C | 0.02 | 0 | Fasting glucose |
| rs9368222 | 6 | 20686996 | 1 | A | C | 0.02 | 0 | Fasting glucose |
| rs2191349 | 7 | 15064309 | 1 | T | G | 0.04 | 0 | Fasting glucose |
| rs2908289 | 7 | 44223942 | 1 | A | G | 0.08 | 0 | Fasting glucose |
| rs6943153 | 7 | 50791579 | 1 | T | C | 0.02 | 0 | Fasting glucose |
| rs983309 | 8 | 9177732 | 1 | T | G | 0.03 | 0 | Fasting glucose |
| rs11558471 | 8 | 118185733 | 1 | A | G | 0.04 | 0 | Fasting glucose |
| rs10814916 | 9 | 4293150 | 1 | C | A | 0.02 | 0 | Fasting glucose |
| rs10811661 | 9 | 22134094 | 0.97 | T | C | 0.03 | 0 | Fasting glucose |
| rs16913693 | 9 | 111680359 | 0.99 | T | G | 0.06 | 0.01 | Fasting glucose |
| rs3829109 | 9 | 139256766 | 0.92 | G | A | 0.02 | 0 | Fasting glucose |
| rs11195502 | 10 | 113039667 | 1 | C | T | 0.04 | 0 | Fasting glucose |
| rs10885122 | 10 | 113042093 | 1 | G | T | 0.04 | 0 | Fasting glucose |
| rs7901695 | 10 | 114754088 | 1 | C | T | 0.03 | 0 | Fasting glucose |
| rs11605924 | 11 | 45873091 | 0.99 | A | C | 0.03 | 0 | Fasting glucose |
| rs11039182 | 11 | 47346723 | 0.99 | T | C | 0.03 | 0 | Fasting glucose |
| rs174576 | 11 | 61603510 | 1 | C | A | 0.03 | 0 | Fasting glucose |
| rs11603334 | 11 | 72432985 | 1 | G | A | 0.03 | 0 | Fasting glucose |
| rs11020124 | 11 | 92690661 | 0.99 | C | T | 0.08 | 0 | Fasting glucose |
| rs2657879 | 12 | 56865338 | 0.99 | G | A | 0.02 | 0 | Fasting glucose |
| rs10747083 | 12 | 133041618 | 0.97 | A | G | 0.02 | 0 | Fasting glucose |
| rs11619319 | 13 | 28487599 | 1 | G | A | 0.03 | 0 | Fasting glucose |
| rs576674 | 13 | 33554302 | 1 | G | A | 0.02 | 0 | Fasting glucose |
| rs3783347 | 14 | 100839261 | 0.99 | G | T | 0.02 | 0 | Fasting glucose |
| rs4502156 | 15 | 62383155 | 0.98 | T | C | 0.03 | 0 | Fasting glucose |
| rs12440695 | 15 | 62435156 | 1 | T | C | 0.01 | 0 | Fasting glucose |
| rs6113722 | 20 | 22557099 | 0.99 | G | A | 0.05 | 0.01 | Fasting glucose |
| rs6072275 | 20 | 39743905 | 1 | A | G | 0.02 | 0 | Fasting glucose |

- 1. **FI**

| **SNP** | **Chr** | **Position** | **Mean imputation quality** | **Effect allele** | **Other allele** | **Estimate** | **SE** | **Trait** |
| --- | --- | --- | --- | --- | --- | --- | --- | --- |
| rs4846565 | 1 | 219722104 | 1 | G | A | 0.03 | 0 | Fasting insulin |
| rs1530559 | 2 | 135755629 | 0.87 | A | G | 0.03 | 0 | Fasting insulin |
| rs10195252 | 2 | 165513091 | 1 | T | C | 0.03 | 0 | Fasting insulin |
| rs2943645 | 2 | 227099180 | 1 | T | C | 0.02 | 0 | Fasting insulin |
| rs17036328 | 3 | 12390484 | 1 | T | C | 0.03 | 0.01 | Fasting insulin |
| rs3822072 | 4 | 89741269 | 0.99 | A | G | 0.02 | 0 | Fasting insulin |
| rs974801 | 4 | 106071064 | 1 | G | A | 0.03 | 0 | Fasting insulin |
| rs6822892 | 4 | 157734675 | 0.99 | A | G | 0.02 | 0 | Fasting insulin |
| rs4865796 | 5 | 53272664 | 0.99 | A | G | 0.03 | 0 | Fasting insulin |
| rs459193 | 5 | 55806751 | 0.98 | G | A | 0.03 | 0 | Fasting insulin |
| rs6912327 | 6 | 34764922 | 1 | T | C | 0.03 | 0.01 | Fasting insulin |
| rs2745353 | 6 | 127452935 | 1 | T | C | 0.03 | 0 | Fasting insulin |
| rs1167800 | 7 | 75176196 | 0.99 | A | G | 0.03 | 0 | Fasting insulin |
| rs2126259 | 8 | 9185146 | 1 | T | C | 0.05 | 0.01 | Fasting insulin |
| rs7903146 | 10 | 114758349 | 1 | C | T | 0.03 | 0.01 | Fasting insulin |
| rs1421085 | 16 | 53800954 | 1 | C | T | 0.04 | 0 | Fasting insulin |
| rs731839 | 19 | 33899065 | 0.99 | G | A | 0.03 | 0 | Fasting insulin |

- 1. **Post 2-hr glucose**

| **SNP** | **Chr** | **Position** | **Mean imputation quality** | **Effect allele** | **Other allele** | **Estimate** | **SE** | **Trait** |
| --- | --- | --- | --- | --- | --- | --- | --- | --- |
| rs1019503 | 5 | 96254817 | 1 | A | G | 0.04 | 0.01 | Glucose post 2-hours |
| rs11672660 | 19 | 46180184 | 1 | T | C | 0.07 | 0.01 | Glucose post 2-hours |
| rs11782386 | 8 | 9201787 | 0.99 | C | T | 0.06 | 0.01 | Glucose post 2-hours |
| rs12255372 | 10 | 114808902 | 1 | T | G | 0.05 | 0.01 | Glucose post 2-hours |
| rs1260326 | 2 | 27730940 | 1 | T | C | 0.03 | 0.01 | Glucose post 2-hours |
| rs1436958 | 15 | 62338797 | 1 | T | G | 0.03 | 0.01 | Glucose post 2-hours |
| rs2877716 | 3 | 123094451 | 1 | C | T | 0.05 | 0.01 | Glucose post 2-hours |
| rs6975024 | 7 | 44231886 | 1 | C | T | 0.06 | 0.01 | Glucose post 2-hours |
| rs7651090 | 3 | 185513392 | 1 | G | A | 0.03 | 0.01 | Glucose post 2-hours |

- 1. **T2DM**

| **SNP** | **Chr** | **Position** | **Mean imputation quality** | **Effect allele** | **Other allele** | **Estimate** | **SE** | **Trait** |
| --- | --- | --- | --- | --- | --- | --- | --- | --- |
| rs340874 | 1 | 214159256 | 1 | C | T | 0.07 | 0.01 | Type 2 diabetes |
| rs780094 | 2 | 27741237 | 1 | C | T | 0.06 | 0.01 | Type 2 diabetes |
| rs77981966 | 2 | 43777964 | 0.98 | C | T | 0.15 | 0.02 | Type 2 diabetes |
| rs243020 | 2 | 60585028 | 1 | G | A | 0.06 | 0.01 | Type 2 diabetes |
| rs75297654 | 2 | 165545615 | 1 | C | T | 0.1 | 0.01 | Type 2 diabetes |
| rs2943645 | 2 | 227099180 | 1 | T | C | 0.09 | 0.01 | Type 2 diabetes |
| rs17036160 | 3 | 12329783 | 0.99 | C | T | 0.13 | 0.01 | Type 2 diabetes |
| rs17676309 | 3 | 64730121 | 0.99 | C | T | 0.07 | 0.01 | Type 2 diabetes |
| rs11708067 | 3 | 123065778 | 0.99 | A | G | 0.1 | 0.01 | Type 2 diabetes |
| rs35510946 | 3 | 185518910 | 1 | A | G | 0.13 | 0.01 | Type 2 diabetes |
| rs1046314 | 4 | 6303955 | 0.99 | A | G | 0.09 | 0.01 | Type 2 diabetes |
| rs7732130 | 5 | 76435004 | 0.99 | G | A | 0.08 | 0.01 | Type 2 diabetes |
| rs35261542 | 6 | 20675792 | 1 | A | C | 0.16 | 0.01 | Type 2 diabetes |
| rs10276674 | 7 | 14922007 | 0.98 | C | T | 0.08 | 0.01 | Type 2 diabetes |
| rs1974620 | 7 | 15065467 | 0.98 | T | C | 0.06 | 0.01 | Type 2 diabetes |
| rs1513272 | 7 | 28200097 | 0.98 | C | T | 0.1 | 0.01 | Type 2 diabetes |
| rs878521 | 7 | 44255643 | 0.98 | A | G | 0.07 | 0.01 | Type 2 diabetes |
| rs13266634 | 8 | 118184783 | 1 | C | T | 0.11 | 0.01 | Type 2 diabetes |
| rs10974438 | 9 | 4291928 | 0.98 | C | A | 0.07 | 0.01 | Type 2 diabetes |
| rs10811660 | 9 | 22134068 | 0.97 | G | A | 0.24 | 0.02 | Type 2 diabetes |
| rs10757283 | 9 | 22134172 | 0.99 | T | C | 0.11 | 0.01 | Type 2 diabetes |
| rs11187140 | 10 | 94466910 | 0.99 | G | A | 0.11 | 0.01 | Type 2 diabetes |
| rs7903146 | 10 | 114758349 | 1 | T | C | 0.31 | 0.01 | Type 2 diabetes |
| rs231360 | 11 | 2692249 | 0.98 | T | C | 0.08 | 0.01 | Type 2 diabetes |
| rs2283220 | 11 | 2755548 | 0.98 | A | G | 0.06 | 0.01 | Type 2 diabetes |
| rs2237895 | 11 | 2857194 | 0.94 | C | A | 0.07 | 0.01 | Type 2 diabetes |
| rs74046911 | 11 | 2858636 | 0.96 | C | T | 0.25 | 0.02 | Type 2 diabetes |
| rs5215 | 11 | 17408630 | 1 | C | T | 0.07 | 0.01 | Type 2 diabetes |
| rs74333814 | 11 | 72457487 | 0.99 | C | T | 0.1 | 0.01 | Type 2 diabetes |
| rs2583941 | 12 | 66204598 | 1 | A | G | 0.1 | 0.01 | Type 2 diabetes |
| rs7961581 | 12 | 71663102 | 0.99 | C | T | 0.06 | 0.01 | Type 2 diabetes |
| rs1169288 | 12 | 121416650 | 0.98 | C | A | 0.09 | 0.01 | Type 2 diabetes |
| rs1800574 | 12 | 121416864 | 0.92 | T | C | 0.2 | 0.03 | Type 2 diabetes |
| rs7172432 | 15 | 62396389 | 1 | A | G | 0.06 | 0.01 | Type 2 diabetes |
| rs3803563 | 15 | 91531352 | 1 | A | C | 0.08 | 0.01 | Type 2 diabetes |
| rs7193144 | 16 | 53810686 | 1 | C | T | 0.13 | 0.01 | Type 2 diabetes |
| rs4430796 | 17 | 36098040 | 1 | G | A | 0.09 | 0.01 | Type 2 diabetes |
| rs7234864 | 18 | 57734857 | 1 | T | C | 0.06 | 0.01 | Type 2 diabetes |
| rs17066842 | 18 | 58040624 | 0.99 | G | A | 0.11 | 0.02 | Type 2 diabetes |
| rs72999033 | 19 | 19366632 | 0.96 | T | C | 0.15 | 0.02 | Type 2 diabetes |
| rs4399645 | 19 | 46166073 | 0.96 | T | C | 0.06 | 0.01 | Type 2 diabetes |
| rs2238689 | 19 | 46178661 | 0.98 | C | T | 0.08 | 0.01 | Type 2 diabetes |
| rs1800961 | 20 | 43042364 | 0.99 | T | C | 0.15 | 0.03 | Type 2 diabetes |

- 1. **Lipid**

| **SNP** | **Chr** | **Position** | **Mean imputation quality** | **Effect allele** | **Other allele** | **Estimate** | **SE** | **Trait** |
| --- | --- | --- | --- | --- | --- | --- | --- | --- |
| rs12748152 | 1 | 27138393 | 0.99 | C | T | 0.05 | 0.01 | HDL |
| rs4660293 | 1 | 40028180 | 1 | A | G | 0.04 | 0 | HDL |
| rs12145743 | 1 | 156700651 | 1 | G | T | 0.02 | 0 | HDL |
| rs4650994 | 1 | 178515312 | 1 | G | A | 0.02 | 0 | HDL |
| rs1689800 | 1 | 182168885 | 0.99 | A | G | 0.03 | 0 | HDL |
| rs4846914 | 1 | 230295691 | 0.98 | A | G | 0.05 | 0 | HDL |
| rs1042034 | 2 | 21225281 | 1 | C | T | 0.07 | 0 | HDL |
| rs12328675 | 2 | 165540800 | 0.99 | C | T | 0.04 | 0.01 | HDL |
| rs1047891 | 2 | 211540507 | 0.9 | C | A | 0.03 | 0 | HDL |
| rs2972146 | 2 | 227100698 | 1 | G | T | 0.03 | 0 | HDL |
| rs2606736 | 3 | 11400249 | 0.99 | C | T | 0.02 | 0 | HDL |
| rs2290547 | 3 | 47061183 | 1 | G | A | 0.03 | 0 | HDL |
| rs2013208 | 3 | 50129399 | 1 | T | C | 0.03 | 0 | HDL |
| rs13326165 | 3 | 52532118 | 1 | A | G | 0.03 | 0 | HDL |
| rs6805251 | 3 | 119560606 | 1 | T | C | 0.02 | 0 | HDL |
| rs17404153 | 3 | 132163200 | 1 | T | G | 0.01 | 0.01 | HDL |
| rs10019888 | 4 | 26062990 | 0.99 | A | G | 0.03 | 0 | HDL |
| rs3822072 | 4 | 89741269 | 0.99 | G | A | 0.03 | 0 | HDL |
| rs2602836 | 4 | 100014805 | 1 | A | G | 0.02 | 0 | HDL |
| rs13107325 | 4 | 103188709 | 0.99 | C | T | 0.07 | 0.01 | HDL |
| rs6450176 | 5 | 53298025 | 1 | G | A | 0.03 | 0 | HDL |
| rs2814944 | 6 | 34552797 | 1 | G | A | 0.03 | 0 | HDL |
| rs998584 | 6 | 43757896 | 0.92 | C | A | 0.03 | 0 | HDL |
| rs1936800 | 6 | 127436064 | 0.99 | C | T | 0.02 | 0 | HDL |
| rs605066 | 6 | 139829666 | 0.99 | T | C | 0.03 | 0 | HDL |
| rs702485 | 7 | 6449272 | 1 | G | A | 0.02 | 0 | HDL |
| rs4142995 | 7 | 17919258 | 1 | G | T | 0.03 | 0 | HDL |
| rs4917014 | 7 | 50305863 | 1 | G | T | 0.02 | 0 | HDL |
| rs17145738 | 7 | 72982874 | 0.98 | T | C | 0.04 | 0.01 | HDL |
| rs4731702 | 7 | 130433384 | 1 | T | C | 0.03 | 0 | HDL |
| rs17173637 | 7 | 150529449 | 1 | T | C | 0.04 | 0.01 | HDL |
| rs9987289 | 8 | 9183358 | 1 | G | A | 0.08 | 0.01 | HDL |
| rs12678919 | 8 | 19844222 | 1 | G | A | 0.16 | 0.01 | HDL |
| rs2293889 | 8 | 116599199 | 1 | G | T | 0.03 | 0 | HDL |
| rs638491 | 9 | 15290012 | 1 | G | A | 0.04 | 0 | HDL |
| rs1883025 | 9 | 107664301 | 0.99 | C | T | 0.07 | 0 | HDL |
| rs970548 | 10 | 46013277 | 1 | C | A | 0.03 | 0 | HDL |
| rs2923084 | 11 | 10388782 | 1 | A | G | 0.03 | 0 | HDL |
| rs3136441 | 11 | 46743247 | 1 | C | T | 0.05 | 0 | HDL |
| rs11246602 | 11 | 51512090 | 1 | C | T | 0.03 | 0.01 | HDL |
| rs174546 | 11 | 61569830 | 1 | C | T | 0.04 | 0 | HDL |
| rs12801636 | 11 | 65391317 | 1 | A | G | 0.02 | 0 | HDL |
| rs499974 | 11 | 75455021 | 1 | C | A | 0.03 | 0 | HDL |
| rs3741298 | 11 | 116657561 | 0.98 | T | C | 0.05 | 0.01 | HDL |
| rs7134375 | 12 | 20473758 | 1 | A | C | 0.02 | 0 | HDL |
| rs11613352 | 12 | 57792580 | 1 | T | C | 0.03 | 0 | HDL |
| rs7134594 | 12 | 110000193 | 1 | T | C | 0.04 | 0 | HDL |
| rs4759375 | 12 | 123796238 | 0.95 | T | C | 0.06 | 0.01 | HDL |
| rs4765127 | 12 | 124460167 | 1 | T | G | 0.03 | 0.01 | HDL |
| rs838880 | 12 | 125261593 | 0.99 | C | T | 0.05 | 0 | HDL |
| rs4983559 | 14 | 105277209 | 1 | G | A | 0.02 | 0 | HDL |
| rs1532085 | 15 | 58683366 | 1 | A | G | 0.11 | 0 | HDL |
| rs2652834 | 15 | 63396867 | 1 | G | A | 0.03 | 0 | HDL |
| rs1121980 | 16 | 53809247 | 1 | G | A | 0.02 | 0 | HDL |
| rs3764261 | 16 | 56993324 | 0.98 | A | C | 0.24 | 0 | HDL |
| rs16942887 | 16 | 67928042 | 1 | A | G | 0.08 | 0.01 | HDL |
| rs2925979 | 16 | 81534790 | 1 | C | T | 0.04 | 0 | HDL |
| rs1877031 | 17 | 37814080 | 1 | A | G | 0.03 | 0 | HDL |
| rs4148005 | 17 | 66882466 | 1 | T | G | 0.03 | 0 | HDL |
| rs4129767 | 17 | 76403984 | 1 | A | G | 0.02 | 0 | HDL |
| rs7241918 | 18 | 47160953 | 1 | T | G | 0.09 | 0.01 | HDL |
| rs12967135 | 18 | 57849023 | 1 | G | A | 0.03 | 0 | HDL |
| rs7255436 | 19 | 8433196 | 1 | A | C | 0.03 | 0.01 | HDL |
| rs737337 | 19 | 11347493 | 1 | T | C | 0.06 | 0.01 | HDL |
| rs731839 | 19 | 33899065 | 0.99 | A | G | 0.02 | 0 | HDL |
| rs4420638 | 19 | 45422946 | 0.81 | A | G | 0.07 | 0.01 | HDL |
| rs17695224 | 19 | 52324216 | 1 | G | A | 0.03 | 0 | HDL |
| rs103294 | 19 | 54797848 | 1 | T | C | 0.05 | 0 | HDL |
| rs1800961 | 20 | 43042364 | 0.99 | C | T | 0.13 | 0.01 | HDL |
| rs6065906 | 20 | 44554015 | 1 | T | C | 0.06 | 0 | HDL |
| rs10903129 | 1 | 25768937 | 1 | G | A | 0.03 | 0 | LDL |
| rs12748152 | 1 | 27138393 | 0.99 | T | C | 0.05 | 0.01 | LDL |
| rs2479409 | 1 | 55504650 | 0.95 | G | A | 0.06 | 0 | LDL |
| rs2131925 | 1 | 63025942 | 1 | T | G | 0.05 | 0 | LDL |
| rs629301 | 1 | 109818306 | 1 | T | G | 0.17 | 0 | LDL |
| rs267733 | 1 | 150958836 | 1 | A | G | 0.03 | 0.01 | LDL |
| rs2642442 | 1 | 220973563 | 0.96 | T | C | 0.04 | 0.01 | LDL |
| rs484084 | 1 | 234857676 | 0.98 | C | T | 0.03 | 0.01 | LDL |
| rs1367117 | 2 | 21263900 | 0.98 | A | G | 0.12 | 0 | LDL |
| rs4299376 | 2 | 44072576 | 1 | G | T | 0.08 | 0 | LDL |
| rs2710642 | 2 | 63149557 | 1 | A | G | 0.02 | 0 | LDL |
| rs10490626 | 2 | 118835841 | 1 | G | A | 0.05 | 0.01 | LDL |
| rs2030746 | 2 | 121309488 | 1 | T | C | 0.02 | 0 | LDL |
| rs1250229 | 2 | 216304384 | 0.98 | C | T | 0.02 | 0 | LDL |
| rs11563251 | 2 | 234679384 | 0.99 | T | C | 0.03 | 0.01 | LDL |
| rs7640978 | 3 | 32533010 | 0.99 | C | T | 0.04 | 0.01 | LDL |
| rs17404153 | 3 | 132163200 | 1 | G | T | 0.03 | 0.01 | LDL |
| rs6831256 | 4 | 3473139 | 1 | G | A | 0.02 | 0 | LDL |
| rs12916 | 5 | 74656539 | 1 | C | T | 0.07 | 0 | LDL |
| rs4530754 | 5 | 122855416 | 1 | A | G | 0.03 | 0 | LDL |
| rs6882076 | 5 | 156390297 | 0.99 | C | T | 0.05 | 0 | LDL |
| rs3757354 | 6 | 16127407 | 1 | C | T | 0.04 | 0 | LDL |
| rs1800562 | 6 | 26093141 | 1 | G | A | 0.06 | 0.01 | LDL |
| rs3798236 | 6 | 116309649 | 0.99 | T | C | 0.02 | 0 | LDL |
| rs1564348 | 6 | 160578860 | 1 | C | T | 0.05 | 0.01 | LDL |
| rs12670798 | 7 | 21607352 | 1 | C | T | 0.03 | 0 | LDL |
| rs4722551 | 7 | 25991826 | 1 | C | T | 0.04 | 0 | LDL |
| rs2072183 | 7 | 44581986 | 1 | C | T | 0.05 | 0 | LDL |
| rs9987289 | 8 | 9183358 | 1 | G | A | 0.07 | 0.01 | LDL |
| rs10102164 | 8 | 55421614 | 0.99 | A | G | 0.03 | 0 | LDL |
| rs2081687 | 8 | 59388565 | 1 | T | C | 0.03 | 0.01 | LDL |
| rs10808546 | 8 | 126495818 | 1 | C | T | 0.05 | 0 | LDL |
| rs11136341 | 8 | 145043543 | 0.96 | G | A | 0.04 | 0.01 | LDL |
| rs3780181 | 9 | 2640759 | 1 | A | G | 0.04 | 0.01 | LDL |
| rs2255141 | 10 | 113933886 | 1 | A | G | 0.03 | 0 | LDL |
| rs11220462 | 11 | 126243952 | 1 | A | G | 0.06 | 0.01 | LDL |
| rs11065987 | 12 | 112072424 | 1 | A | G | 0.03 | 0 | LDL |
| rs1169288 | 12 | 121416650 | 0.98 | C | A | 0.04 | 0 | LDL |
| rs4942486 | 13 | 32953388 | 1 | T | C | 0.02 | 0 | LDL |
| rs8017377 | 14 | 24883887 | 1 | A | G | 0.03 | 0 | LDL |
| rs3764261 | 16 | 56993324 | 0.98 | C | A | 0.05 | 0 | LDL |
| rs2000999 | 16 | 72108093 | 1 | A | G | 0.07 | 0 | LDL |
| rs314253 | 17 | 7091650 | 1 | T | C | 0.02 | 0 | LDL |
| rs7206971 | 17 | 45425115 | 1 | A | G | 0.03 | 0.01 | LDL |
| rs1801689 | 17 | 64210580 | 0.72 | C | A | 0.1 | 0.01 | LDL |
| rs6511720 | 19 | 11202306 | 1 | G | T | 0.22 | 0.01 | LDL |
| rs10401969 | 19 | 19407718 | 0.99 | T | C | 0.12 | 0.01 | LDL |
| rs4420638 | 19 | 45422946 | 0.81 | G | A | 0.23 | 0.01 | LDL |
| rs364585 | 20 | 12962718 | 1 | G | A | 0.02 | 0 | LDL |
| rs2328223 | 20 | 17845921 | 1 | C | A | 0.03 | 0.01 | LDL |
| rs2902940 | 20 | 39091487 | 1 | A | G | 0.03 | 0 | LDL |
| rs6016505 | 20 | 39678289 | 0.98 | T | C | 0.04 | 0.01 | LDL |
| rs5763662 | 22 | 30378703 | 1 | T | C | 0.08 | 0.01 | LDL |
| rs4253772 | 22 | 46627603 | 1 | T | C | 0.03 | 0.01 | LDL |
| rs1077514 | 1 | 23766233 | 0.99 | T | C | 0.03 | 0.01 | Total cholesterol |
| rs10903129 | 1 | 25768937 | 1 | G | A | 0.03 | 0 | Total cholesterol |
| rs2479409 | 1 | 55504650 | 0.95 | G | A | 0.05 | 0 | Total cholesterol |
| rs2131925 | 1 | 63025942 | 1 | T | G | 0.07 | 0 | Total cholesterol |
| rs7515577 | 1 | 93009438 | 1 | A | C | 0.04 | 0.01 | Total cholesterol |
| rs629301 | 1 | 109818306 | 1 | T | G | 0.13 | 0 | Total cholesterol |
| rs2642442 | 1 | 220973563 | 0.96 | T | C | 0.04 | 0.01 | Total cholesterol |
| rs484084 | 1 | 234857676 | 0.98 | C | T | 0.03 | 0.01 | Total cholesterol |
| rs1367117 | 2 | 21263900 | 0.98 | A | G | 0.1 | 0 | Total cholesterol |
| rs1260326 | 2 | 27730940 | 1 | T | C | 0.05 | 0 | Total cholesterol |
| rs4299376 | 2 | 44072576 | 1 | G | T | 0.08 | 0 | Total cholesterol |
| rs10490626 | 2 | 118835841 | 1 | G | A | 0.04 | 0.01 | Total cholesterol |
| rs2030746 | 2 | 121309488 | 1 | T | C | 0.02 | 0 | Total cholesterol |
| rs7570971 | 2 | 135837906 | 0.92 | A | C | 0.03 | 0 | Total cholesterol |
| rs2287623 | 2 | 169830155 | 1 | G | A | 0.03 | 0 | Total cholesterol |
| rs11694172 | 2 | 203532304 | 1 | G | A | 0.03 | 0 | Total cholesterol |
| rs11563251 | 2 | 234679384 | 0.99 | T | C | 0.04 | 0.01 | Total cholesterol |
| rs7956 | 3 | 12624763 | 1 | T | C | 0.03 | 0.01 | Total cholesterol |
| rs7640978 | 3 | 32533010 | 0.99 | C | T | 0.04 | 0.01 | Total cholesterol |
| rs13315871 | 3 | 58381287 | 1 | G | A | 0.04 | 0.01 | Total cholesterol |
| rs6831256 | 4 | 3473139 | 1 | G | A | 0.02 | 0 | Total cholesterol |
| rs12916 | 5 | 74656539 | 1 | C | T | 0.07 | 0 | Total cholesterol |
| rs4530754 | 5 | 122855416 | 1 | A | G | 0.02 | 0 | Total cholesterol |
| rs6882076 | 5 | 156390297 | 0.99 | C | T | 0.05 | 0 | Total cholesterol |
| rs3757354 | 6 | 16127407 | 1 | C | T | 0.03 | 0 | Total cholesterol |
| rs1800562 | 6 | 26093141 | 1 | G | A | 0.06 | 0.01 | Total cholesterol |
| rs2814982 | 6 | 34546560 | 0.99 | C | T | 0.04 | 0.01 | Total cholesterol |
| rs2758886 | 6 | 39250837 | 1 | A | G | 0.02 | 0 | Total cholesterol |
| rs3798236 | 6 | 116309649 | 0.99 | T | C | 0.03 | 0 | Total cholesterol |
| rs9376090 | 6 | 135411228 | 0.99 | T | C | 0.03 | 0 | Total cholesterol |
| rs1564348 | 6 | 160578860 | 1 | C | T | 0.05 | 0 | Total cholesterol |
| rs1997243 | 7 | 1083777 | 1 | G | A | 0.03 | 0.01 | Total cholesterol |
| rs12670798 | 7 | 21607352 | 1 | C | T | 0.04 | 0 | Total cholesterol |
| rs4722551 | 7 | 25991826 | 1 | C | T | 0.03 | 0 | Total cholesterol |
| rs2072183 | 7 | 44581986 | 1 | C | T | 0.04 | 0 | Total cholesterol |
| rs9987289 | 8 | 9183358 | 1 | G | A | 0.08 | 0.01 | Total cholesterol |
| rs1495741 | 8 | 18272881 | 1 | G | A | 0.03 | 0.01 | Total cholesterol |
| rs10102164 | 8 | 55421614 | 0.99 | A | G | 0.03 | 0 | Total cholesterol |
| rs2081687 | 8 | 59388565 | 1 | T | C | 0.04 | 0.01 | Total cholesterol |
| rs2737229 | 8 | 116648565 | 1 | A | C | 0.03 | 0 | Total cholesterol |
| rs10808546 | 8 | 126495818 | 1 | C | T | 0.06 | 0 | Total cholesterol |
| rs11136341 | 8 | 145043543 | 0.96 | G | A | 0.04 | 0.01 | Total cholesterol |
| rs3780181 | 9 | 2640759 | 1 | A | G | 0.04 | 0.01 | Total cholesterol |
| rs638491 | 9 | 15290012 | 1 | G | A | 0.03 | 0 | Total cholesterol |
| rs1883025 | 9 | 107664301 | 0.99 | C | T | 0.07 | 0 | Total cholesterol |
| rs10904908 | 10 | 17260290 | 0.99 | G | A | 0.03 | 0 | Total cholesterol |
| rs970548 | 10 | 46013277 | 1 | C | A | 0.03 | 0 | Total cholesterol |
| rs2255141 | 10 | 113933886 | 1 | A | G | 0.03 | 0 | Total cholesterol |
| rs10128711 | 11 | 18632984 | 0.98 | C | T | 0.03 | 0 | Total cholesterol |
| rs174546 | 11 | 61569830 | 1 | C | T | 0.05 | 0 | Total cholesterol |
| rs3741298 | 11 | 116657561 | 0.98 | C | T | 0.07 | 0.01 | Total cholesterol |
| rs11603023 | 11 | 118486067 | 1 | T | C | 0.02 | 0 | Total cholesterol |
| rs11220462 | 11 | 126243952 | 1 | A | G | 0.05 | 0.01 | Total cholesterol |
| rs4883201 | 12 | 9082581 | 1 | A | G | 0.04 | 0.01 | Total cholesterol |
| rs11065987 | 12 | 112072424 | 1 | A | G | 0.03 | 0 | Total cholesterol |
| rs1169288 | 12 | 121416650 | 0.98 | C | A | 0.03 | 0 | Total cholesterol |
| rs1532085 | 15 | 58683366 | 1 | A | G | 0.05 | 0 | Total cholesterol |
| rs3764261 | 16 | 56993324 | 0.98 | A | C | 0.05 | 0 | Total cholesterol |
| rs2000999 | 16 | 72108093 | 1 | A | G | 0.06 | 0 | Total cholesterol |
| rs314253 | 17 | 7091650 | 1 | T | C | 0.02 | 0 | Total cholesterol |
| rs7206971 | 17 | 45425115 | 1 | A | G | 0.03 | 0.01 | Total cholesterol |
| rs7241918 | 18 | 47160953 | 1 | T | G | 0.06 | 0.01 | Total cholesterol |
| rs6511720 | 19 | 11202306 | 1 | G | T | 0.19 | 0.01 | Total cholesterol |
| rs10401969 | 19 | 19407718 | 0.99 | T | C | 0.14 | 0.01 | Total cholesterol |
| rs4420638 | 19 | 45422946 | 0.81 | G | A | 0.2 | 0.01 | Total cholesterol |
| rs492602 | 19 | 49206417 | 1 | G | A | 0.03 | 0 | Total cholesterol |
| rs2277862 | 20 | 34152782 | 1 | C | T | 0.03 | 0.01 | Total cholesterol |
| rs2902940 | 20 | 39091487 | 1 | A | G | 0.02 | 0 | Total cholesterol |
| rs6016505 | 20 | 39678289 | 0.98 | T | C | 0.04 | 0.01 | Total cholesterol |
| rs1800961 | 20 | 43042364 | 0.99 | C | T | 0.11 | 0.01 | Total cholesterol |
| rs138777 | 22 | 35711098 | 1 | A | G | 0.02 | 0 | Total cholesterol |
| rs4253772 | 22 | 46627603 | 1 | T | C | 0.03 | 0.01 | Total cholesterol |
| rs12748152 | 1 | 27138393 | 0.99 | T | C | 0.04 | 0.01 | Triglycerides |
| rs2131925 | 1 | 63025942 | 1 | T | G | 0.07 | 0 | Triglycerides |
| rs4846914 | 1 | 230295691 | 0.98 | G | A | 0.04 | 0 | Triglycerides |
| rs1042034 | 2 | 21225281 | 1 | T | C | 0.07 | 0 | Triglycerides |
| rs1260326 | 2 | 27730940 | 1 | T | C | 0.11 | 0 | Triglycerides |
| rs10195252 | 2 | 165513091 | 1 | T | C | 0.03 | 0 | Triglycerides |
| rs2972146 | 2 | 227100698 | 1 | T | G | 0.03 | 0 | Triglycerides |
| rs645040 | 3 | 135926622 | 1 | T | G | 0.03 | 0 | Triglycerides |
| rs6831256 | 4 | 3473139 | 1 | G | A | 0.03 | 0 | Triglycerides |
| rs442177 | 4 | 88030261 | 1 | T | G | 0.03 | 0 | Triglycerides |
| rs9686661 | 5 | 55861786 | 0.98 | T | C | 0.04 | 0 | Triglycerides |
| rs6882076 | 5 | 156390297 | 0.99 | C | T | 0.03 | 0 | Triglycerides |
| rs998584 | 6 | 43757896 | 0.92 | A | C | 0.03 | 0 | Triglycerides |
| rs1936800 | 6 | 127436064 | 0.99 | T | C | 0.02 | 0 | Triglycerides |
| rs4722551 | 7 | 25991826 | 1 | T | C | 0.03 | 0 | Triglycerides |
| rs17145738 | 7 | 72982874 | 0.98 | C | T | 0.11 | 0.01 | Triglycerides |
| rs38855 | 7 | 116358044 | 1 | A | G | 0.02 | 0 | Triglycerides |
| rs2271357 | 8 | 10683623 | 1 | A | G | 0.02 | 0 | Triglycerides |
| rs1495741 | 8 | 18272881 | 1 | G | A | 0.04 | 0.01 | Triglycerides |
| rs12678919 | 8 | 19844222 | 1 | A | G | 0.17 | 0.01 | Triglycerides |
| rs10808546 | 8 | 126495818 | 1 | C | T | 0.08 | 0 | Triglycerides |
| rs1832007 | 10 | 5254847 | 1 | A | G | 0.03 | 0 | Triglycerides |
| rs7080386 | 10 | 65048306 | 0.99 | C | A | 0.03 | 0 | Triglycerides |
| rs2068888 | 10 | 94839642 | 1 | G | A | 0.02 | 0 | Triglycerides |
| rs174546 | 11 | 61569830 | 1 | T | C | 0.04 | 0 | Triglycerides |
| rs3741298 | 11 | 116657561 | 0.98 | C | T | 0.15 | 0.01 | Triglycerides |
| rs11613352 | 12 | 57792580 | 1 | C | T | 0.03 | 0 | Triglycerides |
| rs4765127 | 12 | 124460167 | 1 | G | T | 0.03 | 0 | Triglycerides |
| rs2412710 | 15 | 42683787 | 0.95 | A | G | 0.1 | 0.01 | Triglycerides |
| rs1532085 | 15 | 58683366 | 1 | A | G | 0.03 | 0 | Triglycerides |
| rs3198697 | 16 | 15129940 | 1 | C | T | 0.02 | 0 | Triglycerides |
| rs1121980 | 16 | 53809247 | 1 | A | G | 0.02 | 0 | Triglycerides |
| rs8077889 | 17 | 41878166 | 0.99 | C | A | 0.03 | 0 | Triglycerides |
| rs7248104 | 19 | 7224431 | 1 | G | A | 0.02 | 0 | Triglycerides |
| rs10401969 | 19 | 19407718 | 0.99 | T | C | 0.12 | 0.01 | Triglycerides |
| rs731839 | 19 | 33899065 | 0.99 | G | A | 0.02 | 0 | Triglycerides |
| rs439401 | 19 | 45414451 | 1 | C | T | 0.07 | 0 | Triglycerides |
| rs6065906 | 20 | 44554015 | 1 | C | T | 0.05 | 0 | Triglycerides |
| rs5756931 | 22 | 38546033 | 0.98 | T | C | 0.02 | 0 | Triglycerides |
