# Supplementary material for: Genetically Predicted C-Reactive Protein Associated With Postmenopausal Breast Cancer Risk: Interrelation With Estrogen and Cancer Molecular Subtypes Using Mendelian Randomization
Source: Front Oncol. 2021 Feb 3;10:630994. doi: 10.3389/fonc.2020.630994 (PMC7888276; doi:10.3389/fonc.2020.630994)
Supplement: Supplementary file 1 [file DataSheet_1.zip › TableS3_2020Nov18.docx]

Table S3. Two-stage multiple Cox regressions of genome-wide SNPs associated with CRP predicting breast cancer risk

|  |  |  |  | **Allele** | |  | **Stage1**  **Adjustment for age and 10PCs** | | | | | |  | **Stage 2**  **Adjustment for covariates***  **in addition to age and 10PCs** | | | | | | |
| --- | --- | --- | --- | --- | --- | --- | --- | --- | --- | --- | --- | --- | --- | --- | --- | --- | --- | --- | --- | --- |
| **SNP** | **Chr** | **Position¥** | **Gene** | **Ref** | **Alt** |  | **HR** | **(95% CI)** | | | **p** | **p-het†** |  | **HR** | **(95% CI)** | | | **p** | **p-het†** |  |
|  | | | | | | | | | | | | | | | | | | | | |
| GWAS examining CRP as a binary outcome reflecting high immune response and chronic inflammation (CRP > 3.0 mg/L) | | | | | | | | | | | | | | | | | | | | |
| rs2794520 | 1 | 159678816 | *CRPP1/CRP* | C | T |  | 1.02 | (0.90 | - | 1.16) | 0.743 | 0.663 |  | 1.02 | (0.89 | - | 1.16) | 0.791 | 0.669 |  |
| rs2243458 | 12 | 121424490 | *HNF1A* | C | T |  | 0.96 | (0.84 | - | 1.09) | 0.552 | 0.676 |  | 0.94 | (0.82 | - | 1.07) | 0.333 | 0.648 |  |
| rs1169311 | 12 | 121440731 | *C12orf43* | C | T |  | 0.89 | (0.79 | - | 1.01) | 0.065 | 0.270 |  | 0.89 | (0.78 | - | 1.01) | 0.068 | 0.147 |  |
| rs429358 | 19 | 45411941 | *APOE* | T | C |  | 1.14 | (0.94 | - | 1.38) | 0.184 | 0.896 |  | 1.12 | (0.92 | - | 1.36) | 0.264 | 0.806 |  |
| rs5117 | 19 | 45418790 | *APOC1* | T | C |  | 0.94 | (0.76 | - | 1.16) | 0.550 | 0.565 |  | 0.93 | (0.76 | - | 1.15) | 0.506 | 0.801 |  |
|  |  |  |  |  |  |  |  |  |  |  |  |  |  |  |  |  |  |  |  |  |
| GWASs analyzing CRP as a continuous variable that was naturally log-transformed (mg/L) | | | | | | | | | | | | | | | | | | | | |
|  |  |  |  |  |  |  |  |  |  |  |  |  |  |  |  |  |  |  |  |  |
| rs75460349 | 1 | 27180088 | *ZDHHC18* | C | A |  | 0.98 | (0.64 | - | 1.51) | 0.937 | 0.358 |  | 1.02 | (0.66 | - | 1.58) | 0.923 | 0.302 |  |
| rs2293476 | 1 | 40036847 | *PABPC4* | G | C |  | 0.84 | (0.73 | - | 0.97) | 0.016 | 0.724 |  | 0.86 | (0.75 | - | 0.99) | 0.031 | 0.816 |  |
| rs1805096 | 1 | 66102257 | *LEPR* | A | G |  | 0.97 | (0.86 | - | 1.10) | 0.688 | 0.643 |  | 0.98 | (0.86 | - | 1.11) | 0.713 | 0.536 |  |
| rs469772 | 1 | 91530305 | *ZNF644* | T | C |  | 1.08 | (0.93 | - | 1.25) | 0.330 | 0.375 |  | 1.05 | (0.90 | - | 1.23) | 0.521 | 0.345 |  |
| rs4129267 | 1 | 154426264 | *IL6R* | T | C |  | 1.01 | (0.89 | - | 1.14) | 0.896 | 0.772 |  | 0.99 | (0.88 | - | 1.12) | 0.920 | 0.672 |  |
| rs2794520 | 1 | 159678816 | *CRPP1/CRP* | T | C |  | 0.98 | (0.86 | - | 1.11) | 0.743 | 0.663 |  | 0.98 | (0.86 | - | 1.12) | 0.791 | 0.669 |  |
| rs1800947 | 1 | 159683438 | *CRP* | G | C |  | 1.12 | (0.84 | - | 1.50) | 0.449 | 0.321 |  | 1.15 | (0.86 | - | 1.55) | 0.348 | 0.181 |  |
| rs1417938 | 1 | 159684186 | *CRP* | T | C |  | 1.00 | (0.88 | - | 1.14) | 0.966 | 0.438 |  | 1.00 | (0.88 | - | 1.14) | 0.955 | 0.513 |  |
| rs10925027 | 1 | 247612562 | *NLRP3* | C | T |  | 0.96 | (0.84 | - | 1.09) | 0.513 | 0.617 |  | 0.94 | (0.82 | - | 1.08) | 0.401 | 0.256 |  |
| rs12995480 | 2 | 629881 | *TMEM18* | T | C |  | 1.06 | (0.91 | - | 1.23) | 0.474 | 0.204 |  | 1.08 | (0.93 | - | 1.26) | 0.330 | 0.103 |  |
| rs1260326 | 2 | 27730940 | *GCKR* | C | T |  | 0.97 | (0.86 | - | 1.10) | 0.634 | 0.513 |  | 0.95 | (0.84 | - | 1.08) | 0.421 | 0.530 |  |
| rs4246598 | 2 | 88438050 | *FABP1* | C | A |  | 1.12 | (0.99 | - | 1.26) | 0.078 | 0.675 |  | 1.09 | (0.97 | - | 1.24) | 0.147 | 0.646 |  |
| rs9284725 | 2 | 102744854 | *IL1R1* | A | C |  | 0.94 | (0.82 | - | 1.08) | 0.373 | 0.614 |  | 0.93 | (0.81 | - | 1.07) | 0.313 | 0.700 |  |
| rs13409371 | 2 | 113838145 | *IL1F10* | G | A |  | 1.06 | (0.93 | - | 1.20) | 0.380 | 0.740 |  | 1.07 | (0.95 | - | 1.22) | 0.273 | 0.860 |  |
| rs1441169 | 2 | 214033530 | *IKZF2* | G | A |  | 0.95 | (0.85 | - | 1.08) | 0.443 | 0.217 |  | 0.95 | (0.84 | - | 1.08) | 0.424 | 0.271 |  |
| rs2352975 | 3 | 49891885 | *TRAIP* | T | C |  | 0.82 | (0.71 | - | 0.96) | 0.014 | 0.124 |  | 0.83 | (0.71 | - | 0.97) | 0.018 | < 0.001 |  |
| rs1514895 | 3 | 170705693 | *EIF5A2* | A | G |  | 1.08 | (0.94 | - | 1.24) | 0.264 | 0.980 |  | 1.07 | (0.94 | - | 1.23) | 0.317 | 0.994 |  |

Table S3 (Continued)

|  |  |  |  | **Allele** | |  | **Stage1**  **Adjustment for age and 10PCs** | | | | | |  | **Stage 2**  **Adjustment for covariates***  **in addition to age and 10PCs** | | | | | | |
| --- | --- | --- | --- | --- | --- | --- | --- | --- | --- | --- | --- | --- | --- | --- | --- | --- | --- | --- | --- | --- |
| **SNP** | **Chr** | **Position¥** | **Gene** | **Ref** | **Alt** |  | **HR** | **(95% CI)** | | | **p** | **p-het†** |  | **HR** | **(95% CI)** | | | **p** | **p-het†** | |
|  |  |  |  |  |  |  |  |  |  |  |  |  |  |  |  |  |  |  |  |  |
| (Cont.) GWASs analyzing CRP as a continuous variable that was naturally log-transformed (mg/L) | | | | | | | | | | | | | | | | | | | | |
|  |  |  |  |  |  |  |  |  |  |  |  |  |  |  |  |  |  |  |  |  |
| rs4705952 | 5 | 131839618 | *IRF1* | A | G |  | 1.03 | (0.87 | - | 1.23) | 0.706 | 0.020 |  | 1.01 | (0.85 | - | 1.21) | 0.875 | 0.010 | |
| rs17658229 | 5 | 172191052 | *DUSP1* | T | C |  | 1.10 | (0.75 | - | 1.61) | 0.625 | 0.554 |  | 1.11 | (0.76 | - | 1.63) | 0.579 | 0.557 | |
| rs9271608 | 6 | 32591588 | *HLA-DQA1* | A | G |  | 0.87 | (0.67 | - | 1.14) | 0.324 | 0.041 |  | 0.86 | (0.66 | - | 1.13) | 0.282 | 0.041 | |
| rs12202641 | 6 | 116314634 | *FRK* | T | C |  | 1.09 | (0.95 | - | 1.25) | 0.243 | 0.209 |  | 1.09 | (0.95 | - | 1.26) | 0.220 | 0.166 | |
| rs6901250 | 6 | 117114025 | *GPRC6A* | G | A |  | 1.00 | (0.88 | - | 1.14) | 0.982 | 0.629 |  | 1.00 | (0.87 | - | 1.14) | 0.962 | 0.620 | |
| rs1490384 | 6 | 126851160 | *CENPW* | T | C |  | 0.88 | (0.78 | - | 0.99) | 0.037 | 0.668 |  | 0.89 | (0.79 | - | 1.01) | 0.067 | 0.814 | |
| rs9385532 | 6 | 130371227 | *L3MBTL3* | T | C |  | 1.06 | (0.93 | - | 1.20) | 0.386 | 0.389 |  | 1.06 | (0.93 | - | 1.20) | 0.406 | 0.291 | |
| rs1880241 | 7 | 22759469 | *IL6* | G | A |  | 1.09 | (0.97 | - | 1.23) | 0.159 | 0.765 |  | 1.09 | (0.97 | - | 1.24) | 0.147 | 0.858 | |
| rs2710804 | 7 | 36084529 | *EEPD1* | T | C |  | 1.00 | (0.88 | - | 1.13) | 0.958 | 0.042 |  | 1.00 | (0.88 | - | 1.14) | 0.972 | 0.044 | |
| rs13233571 | 7 | 72971231 | *BCL7B* | T | C |  | 1.08 | (0.90 | - | 1.30) | 0.394 | 0.502 |  | 1.05 | (0.87 | - | 1.26) | 0.607 | 0.464 | |
| rs4841132 | 8 | 9183596 | *PPP1R3B* | A | G |  | 0.96 | (0.77 | - | 1.19) | 0.715 | 0.829 |  | 0.94 | (0.76 | - | 1.17) | 0.597 | 0.916 | |
| rs2064009 | 8 | 117007850 | *TRPS1* | C | T |  | 1.07 | (0.94 | - | 1.20) | 0.304 | 0.087 |  | 1.07 | (0.94 | - | 1.21) | 0.312 | 0.072 | |
| rs2891677 | 8 | 126344208 | *NSMCE2* | C | T |  | 0.97 | (0.86 | - | 1.10) | 0.648 | 0.945 |  | 0.97 | (0.86 | - | 1.10) | 0.678 | 0.848 | |
| rs643434 | 9 | 136142355 | *ABO* | G | A |  | 0.98 | (0.87 | - | 1.11) | 0.778 | 0.920 |  | 1.00 | (0.88 | - | 1.13) | 0.964 | 0.879 | |
| rs1051338 | 10 | 91007360 | *LIPA* | T | G |  | 0.93 | (0.82 | - | 1.06) | 0.292 | 0.753 |  | 0.94 | (0.83 | - | 1.08) | 0.397 | 0.693 | |
| rs10832027 | 11 | 13357183 | *ARNTL* | G | A |  | 0.90 | (0.79 | - | 1.02) | 0.109 | 0.998 |  | 0.91 | (0.80 | - | 1.04) | 0.152 | 0.955 | |
| rs10838687 | 11 | 47312892 | *MADD* | G | T |  | 0.98 | (0.84 | - | 1.14) | 0.770 | 0.752 |  | 0.98 | (0.84 | - | 1.14) | 0.762 | 0.867 | |
| rs1582763 | 11 | 60021948 | *MS4A4A* | A | G |  | 1.01 | (0.89 | - | 1.14) | 0.891 | 0.365 |  | 1.02 | (0.90 | - | 1.16) | 0.703 | 0.306 | |
| rs7121935 | 11 | 72496148 | *STARD10* | A | G |  | 0.90 | (0.79 | - | 1.02) | 0.105 | 0.245 |  | 0.92 | (0.81 | - | 1.05) | 0.210 | 0.168 | |
| rs11108056 | 12 | 95855385 | *METAP2* | G | C |  | 0.93 | (0.82 | - | 1.06) | 0.294 | 0.121 |  | 0.95 | (0.83 | - | 1.09) | 0.457 | 0.175 | |
| rs10778215 | 12 | 103537266 | *C12orf42* | A | T |  | 1.03 | (0.91 | - | 1.16) | 0.685 | 0.259 |  | 1.03 | (0.92 | - | 1.17) | 0.600 | 0.364 | |
| rs7310409 | 12 | 121424861 | *HNF1A* | A | G |  | 1.08 | (0.96 | - | 1.23) | 0.199 | 0.326 |  | 1.10 | (0.98 | - | 1.25) | 0.117 | 0.174 | |
| rs2239222 | 14 | 73011885 | *RGS6* | A | G |  | 1.00 | (0.87 | - | 1.14) | 0.967 | 0.653 |  | 1.00 | (0.87 | - | 1.14) | 0.954 | 0.639 | |
| rs112635299 | 14 | 94838142 | *SERPINA1/SERPINA2P* | T | G |  | 0.89 | (0.53 | - | 1.48) | 0.655 | 0.842 |  | 0.84 | (0.50 | - | 1.40) | 0.496 | 0.925 | |

Table S3 (Continued)

|  |  |  |  | **Allele** | |  | **Stage1**  **Adjustment for age and 10PCs** | | | | | |  | **Stage 2**  **Adjustment for covariates***  **in addition to age and 10PCs** | | | | | | |
| --- | --- | --- | --- | --- | --- | --- | --- | --- | --- | --- | --- | --- | --- | --- | --- | --- | --- | --- | --- | --- |
| **SNP** | **Chr** | **Position¥** | **Gene** | **Ref** | **Alt** |  | **HR** | **(95% CI)** | | | **p** | **p-het†** |  | **HR** | **(95% CI)** | | | **p** | **p-het†** | |
|  |  |  |  |  |  |  |  |  |  |  |  |  |  |  |  |  |  |  |  |  |
| (Cont.) GWASs analyzing CRP as a continuous variable that was naturally log-transformed (mg/L) | | | | | | | | | | | | | | | | | | | | |
|  |  |  |  |  |  |  |  |  |  |  |  |  |  |  |  |  |  |  |  |  |
| rs4774590 | 15 | 51745277 | *DMXL2* | A | G |  | 1.00 | (0.89 | - | 1.14) | 0.966 | 0.778 |  | 1.01 | (0.90 | - | 1.15) | 0.823 | 0.888 | |
| rs1189402 | 15 | 53728154 | *WDR72* | G | A |  | 1.01 | (0.89 | - | 1.14) | 0.911 | 0.809 |  | 1.01 | (0.89 | - | 1.15) | 0.861 | 0.749 | |
| rs340005 | 15 | 60878030 | *RORA* | G | A |  | 0.93 | (0.82 | - | 1.06) | 0.277 | 0.827 |  | 0.93 | (0.82 | - | 1.06) | 0.281 | 0.747 | |
| rs10521222 | 16 | 51158710 | *SALL1* | T | C |  | 0.97 | (0.57 | - | 1.63) | 0.895 | 0.368 |  | 1.02 | (0.60 | - | 1.72) | 0.953 | 0.382 | |
| rs1558902 | 16 | 53803574 | *FTO* | T | A |  | 1.06 | (0.93 | - | 1.20) | 0.396 | 0.131 |  | 1.05 | (0.93 | - | 1.20) | 0.412 | 0.090 | |
| rs178810 | 17 | 16097430 | *NCOR1* | C | T |  | 0.93 | (0.81 | - | 1.07) | 0.299 | 0.089 |  | 0.94 | (0.82 | - | 1.08) | 0.391 | 0.028 | |
| rs10512597 | 17 | 72699833 | *CD300LF/RAB37* | T | C |  | 1.03 | (0.88 | - | 1.20) | 0.751 | 0.261 |  | 1.04 | (0.89 | - | 1.21) | 0.640 | 0.178 | |
| rs2852151 | 18 | 12841176 | *PTPN2* | G | A |  | 1.02 | (0.90 | - | 1.16) | 0.746 | 0.143 |  | 1.04 | (0.91 | - | 1.18) | 0.588 | 0.090 | |
| rs4092465 | 18 | 55080437 | *ONECUT2* | A | G |  | 0.97 | (0.85 | - | 1.12) | 0.700 | 0.960 |  | 0.98 | (0.85 | - | 1.12) | 0.741 | 0.997 | |
| rs12960928 | 18 | 57897803 | *MC4R* | T | C |  | 0.99 | (0.86 | - | 1.13) | 0.873 | 0.347 |  | 0.97 | (0.85 | - | 1.11) | 0.667 | 0.159 | |
| rs4420638 | 19 | 45422946 | *APOC1* | G | A |  | 0.86 | (0.67 | - | 1.09) | 0.218 | 0.928 |  | 0.85 | (0.67 | - | 1.09) | 0.202 | 0.873 | |
| rs1800961 | 20 | 43042364 | *HNF4A* | T | C |  | 1.14 | (0.81 | - | 1.62) | 0.450 | 0.889 |  | 1.11 | (0.78 | - | 1.58) | 0.574 | 0.892 | |
| rs2315008 | 20 | 62343956 | *ZGPAT* | T | G |  | 1.03 | (0.90 | - | 1.17) | 0.667 | 0.073 |  | 1.03 | (0.90 | - | 1.17) | 0.659 | 0.098 | |
| rs2836878 | 21 | 40465534 | *PSMG1* | A | G |  | 0.90 | (0.79 | - | 1.04) | 0.157 | 0.543 |  | 0.91 | (0.79 | - | 1.04) | 0.160 | 0.670 | |
| rs6001193 | 22 | 39074737 | *TOMM22* | G | A |  | 1.00 | (0.87 | - | 1.15) | 0.952 | 0.843 |  | 1.00 | (0.86 | - | 1.15) | 0.953 | 0.676 | |

Alt, alternative; Chr, chromosome; CI, confidence interval; CRP, C-reactive protein; GWAS, genome-wide association study; HR, hazard ratio; PCs, principal components; Ref, reference; SFA, saturated fatty acids; SNP, single-nucleotide polymorphism.

* Covariates adjusted in the analyses include education; annual family income; family history of breast cancer; body mass index; waist-to-hip ratio; physical activity; depressive symptoms; number of cigarettes per day; dietary alcohol in g/day; % calories from SFA/day; age at menopause; duration of oral contraceptive use; and durations of exogenous estrogen [E]–only use and E plus progestin use.

¥ GRCh 37 coordinated.

† Heterogeneity in estimates among the 5 WHI sub-GWASs was evaluated by Cochran’s Q test with fixed effects.
