# Supplementary material for: Genetically Predicted C-Reactive Protein Associated With Postmenopausal Breast Cancer Risk: Interrelation With Estrogen and Cancer Molecular Subtypes Using Mendelian Randomization
Source: Front Oncol. 2021 Feb 3;10:630994. doi: 10.3389/fonc.2020.630994 (PMC7888276; doi:10.3389/fonc.2020.630994)
Supplement: Supplementary file 1 [file DataSheet_1.zip › TableS4_2020Nov18.docx]

Table S4. Mendelian randomization analysis: the effect of genetically predicted chronic inflammation status on breast cancer risk by subgroup

| **GWAS examining CRP as a binary outcome reflecting high immune response and chronic inflammation (CRP > 3.0 mg/L)** | | | | | | | | | | | | | | | | |
| --- | --- | --- | --- | --- | --- | --- | --- | --- | --- | --- | --- | --- | --- | --- | --- | --- |
|  | |  | **Stage 1**  **Adjustment for age and 10 PCs** | | | | | |  | **Stage 2**  **Adjustment for covariates***  **in addition to age and 10PCs** | | | | | | |
| **Subgroup** | | **Analytic method** | **HR¶** | **(95% CI)** | | | **p** | **p-het†** |  | **HR¶** | **(95% CI)** | | | **p** | **p-het†** | |
| **Family history of breast cancer** | **No** | Inverse-variance weighted | 0.99 | (0.57 | - | 1.72) | 0.974 | 0.285 |  | 0.96 | (0.55 | - | 1.69) | 0.858 | | 0.272 |
|  |  | Weighted median | 0.97 | 0.62 | - | 1.53) | 0.896 |  |  | 0.88 | (0.54 | - | 1.44) | 0.617 | |  |
|  |  | Penalized weighted median | 0.97 | (0.62 | - | 1.52) | 0.895 |  |  | 0.88 | (0.56 | - | 1.40) | 0.596 | |  |
|  |  | MR-Egger: slope | 2.71 | (0.07 | - | 110.40) | 0.455 |  |  | 2.33 | (0.05 | - | 114.08) | 0.539 | |  |
|  |  | intercept | 0.80 | 0.36 | - | 1.79) | 0.446 |  |  | 0.82 | (0.35 | - | 1.91) | 0.516 | |  |
|  | **Yes** | Inverse-variance weighted | 0.71 | (0.51 | - | 0.98) | 0.043 | 0.970 |  | 0.51 | (0.35 | - | 0.75) | 0.008 | | 0.967 |
|  |  | Weighted median | 0.71 | (0.34 | - | 1.51) | 0.378 |  |  | 0.46 | (0.20 | - | 1.03) | 0.058 | |  |
|  |  | Penalized weighted median | 0.71 | (0.33 | - | 1.54) | 0.388 |  |  | 0.46 | (0.19 | - | 1.07) | 0.070 | |  |
|  |  | MR-Egger: slope | 2.35 | (0.80 | - | 6.86) | 0.085 |  |  | 1.82 | (0.35 | - | 9.33) | 0.329 | |  |
|  |  | intercept | 0.77 | (0.61 | - | 0.97) | 0.036 |  |  | 0.76 | (0.53 | - | 1.08) | 0.088 | |  |
| **ER/PR** | **Positive** | Inverse-variance weighted | 0.84 | (0.59 | - | 1.19) | 0.234 | 0.746 |  | 1.04 | (0.49 | - | 2.20) | 0.893 | | 0.183 |
|  |  | Weighted median | 0.91 | (0.59 | - | 1.40) | 0.653 |  |  | 0.85 | (0.46 | - | 1.57) | 0.604 | |  |
|  |  | Penalized weighted median | 0.91 | (0.58 | - | 1.42) | 0.664 |  |  | 0.85 | (0.47 | - | 1.54) | 0.593 | |  |
|  |  | MR-Egger: slope | 1.80 | (0.19 | - | 17.36) | 0.470 |  |  | 0.32 | (0.00 | - | 50.27) | 0.528 | |  |
|  |  | intercept | 0.84 | (0.52 | - | 1.38 | 0.354 |  |  | 1.30 | (0.43 | - | 3.93) | 0.509 | |  |
|  | **Negative** | Inverse-variance weighted | 21.04 | (0.00 | - | 1.75E+05) | 0.402 | 0.054 |  | NA | NA | - | NA | NA | | NA |
|  |  | Weighted median | 22.56 | (0.06 | - | 9262.90) | 0.310 |  |  | NA | NA | - | NA | NA | |  |
|  |  | Penalized weighted median | 22.56 | (0.05 | - | 9806.12) | 0.315 |  |  | NA | NA | - | NA | NA | |  |
|  |  | MR-Egger: slope | NA | NA | - | NA | NA |  |  | NA | NA | - | NA | NA | |  |
|  |  | intercept | 167.26 | (0.02 | - | 1.17E+06) | 0.163 |  |  | NA | NA | - | NA | NA | |  |

Table S4 (Continued)

| **GWAS examining CRP as a binary outcome reflecting high immune response and chronic inflammation (CRP > 3.0 mg/L)** | | | | | | | | | | | | | | | | |
| --- | --- | --- | --- | --- | --- | --- | --- | --- | --- | --- | --- | --- | --- | --- | --- | --- |
|  | |  | **Stage 1**  **Adjustment for age and 10 PCs** | | | | | |  | **Stage 2**  **Adjustment for covariates***  **in addition to age and 10PCs** | | | | | | |
| **Subgroup** | | **Analytic method** | **HR¶** | **(95% CI)** | | | **p** | **p-het†** |  | **HR¶** | **(95% CI)** | | | **p** | **p-het†** | |
| **Dietary alcohol** | **≤ 1 drink/d** | Inverse-variance weighted | 0.88 | (0.54 | - | 1.43) | 0.493 | 0.392 |  | 0.87 | (0.58 | - | 1.28) | 0.363 | | 0.620 |
|  |  | Weighted median | 0.83 | (0.53 | - | 1.28) | 0.391 |  |  | 0.82 | (0.54 | - | 1.24) | 0.340 | |  |
|  |  | Penalized weighted median | 0.83 | (0.53 | - | 1.28) | 0.393 |  |  | 0.82 | (0.53 | - | 1.26) | 0.355 | |  |
|  |  | MR-Egger: slope | 2.36 | (0.10 | - | 56.05) | 0.452 |  |  | 1.57 | (0.10 | - | 24.39) | 0.635 | |  |
|  |  | intercept | 0.80 | (0.41 | - | 1.60) | 0.386 |  |  | 0.88 | (0.48 | - | 1.59) | 0.531 | |  |
|  | **> 1 drink/d** | Inverse-variance weighted | 0.93 | (0.53 | - | 1.64) | 0.753 | 0.873 |  | 0.92 | (0.41 | - | 2.05) | 0.779 | | 0.709 |
|  |  | Weighted median | 0.94 | (0.39 | - | 2.28) | 0.895 |  |  | 0.98 | (0.35 | - | 2.75) | 0.973 | |  |
|  |  | Penalized weighted median | 0.94 | (0.40 | - | 2.23) | 0.892 |  |  | 0.98 | (0.37 | - | 2.63) | 0.972 | |  |
|  |  | MR-Egger: slope | 5.60 | (0.42 | - | 75.18) | 0.125 |  |  | 12.44 | (0.44 | - | 350.82) | 0.096 | |  |
|  |  | intercept | 0.68 | (0.39 | - | 1.18) | 0.112 |  |  | 0.56 | (0.27 | - | 1.16) | 0.086 | |  |
| **% calories from SFA** | **< 9.0%** | Inverse-variance weighted | 0.86 | (0.57 | - | 1.28) | 0.339 | 0.928 |  | 0.76 | (0.42 | - | 1.36) | 0.254 | | 0.796 |
|  |  | Weighted median | 0.86 | (0.42 | - | 1.77) | 0.686 |  |  | 0.79 | (0.36 | - | 1.75) | 0.566 | |  |
|  |  | Penalized weighted median | 0.86 | (0.42 | - | 1.79) | 0.689 |  |  | 0.79 | (0.35 | - | 1.78) | 0.574 | |  |
|  |  | MR-Egger: slope | 3.10 | (0.59 | - | 16.33) | 0.118 |  |  | 4.84 | (0.42 | - | 56.41) | 0.134 | |  |
|  |  | intercept | 0.75 | (0.52 | - | 1.08) | 0.087 |  |  | 0.66 | (0.38 | - | 1.13) | 0.092 | |  |
|  | **≥ 9.0%** | Inverse-variance weighted | 0.95 | (0.50 | - | 1.77) | 0.814 | 0.182 |  | 0.90 | (0.52 | - | 1.54) | 0.605 | | 0.338 |
|  |  | Weighted median | 0.83 | (0.51 | - | 1.33) | 0.435 |  |  | 0.82 | (0.51 | - | 1.32) | 0.416 | |  |
|  |  | Penalized weighted median | 0.83 | (0.51 | - | 1.34) | 0.443 |  |  | 0.82 | (0.51 | - | 1.33) | 0.427 | |  |
|  |  | MR-Egger: slope | 4.37 | (0.09 | - | 205.55) | 0.310 |  |  | 3.04 | (0.10 | - | 94.50) | 0.379 | |  |
|  |  | intercept | 0.72 | (0.31 | - | 1.64) | 0.289 |  |  | 0.77 | (0.37 | - | 1.60) | 0.334 | |  |

Table S4 (Continued)

| **GWAS examining CRP as a binary outcome reflecting high immune response and chronic inflammation (CRP > 3.0 mg/L)** | | | | | | | | | | | | | | | | |
| --- | --- | --- | --- | --- | --- | --- | --- | --- | --- | --- | --- | --- | --- | --- | --- | --- |
|  | |  | **Stage 1**  **Adjustment for age and 10 PCs** | | | | | |  | **Stage 2**  **Adjustment for covariates***  **in addition to age and 10PCs** | | | | | | |
| **Subgroup** | | **Analytic method** | **HR¶** | **(95% CI)** | | | **p** | **p-het†** |  | **HR¶** | **(95% CI)** | | | **p** | **p-het†** | |
| **HER2/neu** | **Positive** | Inverse-variance weighted | 0.39 | (0.03 | - | 5.13) | 0.368 | 0.619 |  | NA | NA | - | NA | NA | | NA |
|  |  | Weighted median | 0.32 | (0.02 | - | 5.88) | 0.443 |  |  | NA | NA | - | NA | NA | |  |
|  |  | Penalized weighted median | 0.32 | (0.02 | - | 5.03) | 0.418 |  |  | NA | NA | - | NA | NA | |  |
|  |  | MR-Egger: slope | 0.52 | (0.00 | - | 5.81E+07) | 0.918 |  |  | NA | NA | - | NA | NA | |  |
|  |  | intercept | 0.94 | (0.01 | - | 61.52) | 0.963 |  |  | 0.00 | (0.00 | - | 9.18E+36) | 0.368 | |  |
|  | **Negative** | Inverse-variance weighted | 0.70 | (0.32 | - | 1.54) | 0.279 | 0.120 |  | 0.87 | (0.35 | - | 2.16) | 0.684 | | 0.148 |
|  |  | Weighted median | 0.80 | (0.46 | - | 1.38) | 0.418 |  |  | 1.10 | (0.56 | - | 2.16) | 0.773 | |  |
|  |  | Penalized weighted median | 0.83 | (0.47 | - | 1.46) | 0.512 |  |  | 1.16 | (0.59 | - | 2.29) | 0.666 | |  |
|  |  | MR-Egger: slope | 3.94 | (0.03 | - | 552.16) | 0.442 |  |  | 1.34 | (0.00 | - | 1054.46) | 0.898 | |  |
|  |  | intercept | 0.68 | (0.24 | - | 1.99) | 0.341 |  |  | 0.91 | (0.21 | - | 3.88) | 0.846 | |  |
| **OC use** | **< 5 years** | Inverse-variance weighted | 0.87 | (0.53 | - | 1.44) | 0.494 | 0.431 |  | 0.88 | (0.55 | - | 1.42) | 0.494 | | 0.494 |
|  |  | Weighted median | 0.77 | (0.49 | - | 1.21) | 0.257 |  |  | 0.79 | (0.49 | - | 1.27) | 0.329 | |  |
|  |  | Penalized weighted median | 0.77 | (0.48 | - | 1.23) | 0.267 |  |  | 0.79 | (0.49 | - | 1.26) | 0.321 | |  |
|  |  | MR-Egger: slope | 1.40 | (0.04 | - | 52.77) | 0.789 |  |  | 1.31 | (0.04 | - | 42.74) | 0.819 | |  |
|  |  | intercept | 0.90 | (0.41 | - | 1.99) | 0.705 |  |  | 0.92 | (0.43 | - | 1.95) | 0.734 | |  |
|  | **≥ 5 years** | Inverse-variance weighted | 0.85 | (0.43 | - | 1.68) | 0.540 | 0.586 |  | 0.86 | (0.40 | - | 1.83) | 0.603 | | 0.498 |
|  |  | Weighted median | 0.77 | (0.38 | - | 1.56) | 0.463 |  |  | 0.68 | (0.33 | - | 1.42) | 0.305 | |  |
|  |  | Penalized weighted median | 0.77 | (0.37 | - | 1.58) | 0.469 |  |  | 0.68 | (0.32 | - | 1.43) | 0.310 | |  |
|  |  | MR-Egger: slope | 6.48 | (0.18 | - | 234.11) | 0.196 |  |  | 6.03 | (0.07 | - | 543.02) | 0.294 | |  |
|  |  | intercept | 0.64 | (0.30 | - | 1.39) | 0.164 |  |  | 0.65 | (0.25 | - | 1.72) | 0.256 | |  |

Table S4 (Continued)

| **GWAS examining CRP as a binary outcome reflecting high immune response and chronic inflammation (CRP > 3.0 mg/L)** | | | | | | | | | | | | | | | | |
| --- | --- | --- | --- | --- | --- | --- | --- | --- | --- | --- | --- | --- | --- | --- | --- | --- |
|  | |  | **Stage 1**  **Adjustment for age and 10 PCs** | | | | | |  | **Stage 2**  **Adjustment for covariates***  **in addition to age and 10PCs** | | | | | | |
| **Subgroup** | | **Analytic method** | **HR¶** | **(95% CI)** | | | **p** | **p-het†** |  | **HR¶** | **(95% CI)** | | | **p** | **p-het†** | |
| **MET** | **≥ 10** | Inverse-variance weighted | 0.87 | (0.53 | - | 1.45) | 0.498 | 0.700 |  | 0.81 | (0.53 | - | 1.25) | 0.251 | | 0.827 |
|  |  | Weighted median | 0.81 | (0.44 | - | 1.49) | 0.491 |  |  | 0.80 | (0.44 | - | 1.46) | 0.466 | |  |
|  |  | Penalized weighted median | 0.81 | (0.44 | - | 1.49) | 0.494 |  |  | 0.80 | (0.45 | - | 1.42) | 0.447 | |  |
|  |  | MR-Egger: slope | 5.12 | (0.66 | - | 39.56) | 0.085 |  |  | 3.11 | (0.37 | - | 25.84) | 0.187 | |  |
|  |  | intercept | 0.68 | (0.44 | - | 1.06) | 0.068 |  |  | 0.75 | (0.47 | - | 1.18) | 0.134 | |  |
|  | **< 10** | Inverse-variance weighted | 0.98 | (0.58 | - | 1.65) | 0.904 | 0.473 |  | 0.90 | (0.53 | - | 1.53) | 0.617 | | 0.480 |
|  |  | Weighted median | 1.01 | (0.59 | - | 1.71) | 0.980 |  |  | 0.87 | (0.51 | - | 1.48) | 0.604 | |  |
|  |  | Penalized weighted median | 1.01 | (0.59 | - | 1.71) | 0.980 |  |  | 0.87 | (0.51 | - | 1.47) | 0.595 | |  |
|  |  | MR-Egger: slope | 1.77 | (0.04 | - | 74.47) | 0.662 |  |  | 1.91 | (0.05 | - | 74.22) | 0.612 | |  |
|  |  | intercept | 0.88 | (0.39 | - | 1.98) | 0.644 |  |  | 0.85 | (0.38 | - | 1.88) | 0.554 | |  |
| **E+P** | **≥ 5 years** | Inverse-variance weighted | 0.96 | (0.24 | - | 3.83) | 0.940 | 0.527 |  | 1.02 | (0.00 | - | 8754.06) | 0.996 | | 0.000 |
|  |  | Weighted median | 0.85 | (0.22 | - | 3.28) | 0.814 |  |  | 2.18 | (0.46 | - | 10.35) | 0.328 | |  |
|  |  | Penalized weighted median | 0.85 | (0.22 | - | 3.26) | 0.814 |  |  | 1.60 | (0.31 | - | 8.24) | 0.574 | |  |
|  |  | MR-Egger: slope | 237.03 | (0.23 | - | 2.42E+05) | 0.087 |  |  | NA | NA | - | NA | NA | |  |
|  |  | intercept | 0.32 | (0.08 | - | 1.32) | 0.083 |  |  | 12.51 | (0.00 | - | 9.97E+07) | 0.648 | |  |
| **WHR** | **≤ 0.85** | Inverse-variance weighted | 0.99 | (0.65 | - | 1.52) | 0.964 | 0.637 |  | 0.96 | (0.63 | - | 1.46) | 0.793 | | 0.651 |
|  |  | Weighted median | 0.99 | (0.61 | - | 1.61) | 0.961 |  |  | 0.91 | (0.56 | - | 1.48) | 0.709 | |  |
|  |  | Penalized weighted median | 0.99 | (0.62 | - | 1.58) | 0.960 |  |  | 0.91 | (0.56 | - | 1.48) | 0.709 | |  |
|  |  | MR-Egger: slope | 0.95 | (0.04 | - | 23.35) | 0.965 |  |  | 0.71 | (0.03 | - | 16.32) | 0.754 | |  |
|  |  | intercept | 1.01 | (0.51 | - | 2.02) | 0.970 |  |  | 1.07 | (0.54 | - | 2.10) | 0.781 | |  |
|  | **> 0.85** | Inverse-variance weighted | 0.75 | (0.29 | - | 1.90) | 0.433 | 0.175 |  | 0.65 | (0.23 | - | 1.85) | 0.319 | | 0.114 |
|  |  | Weighted median | 0.72 | (0.37 | - | 1.41) | 0.334 |  |  | 0.57 | (0.26 | - | 1.23) | 0.151 | |  |
|  |  | Penalized weighted median | 0.72 | (0.36 | - | 1.44) | 0.349 |  |  | 0.57 | (0.27 | - | 1.19) | 0.131 | |  |
|  |  | MR-Egger: slope | 24.43 | (1.67 | - | 356.72) | 0.032 |  |  | 32.71 | (2.07 | - | 516.91) | 0.028 | |  |
|  |  | intercept | 0.47 | (0.26 | - | 0.83) | 0.025 |  |  | 0.42 | (0.23 | - | 0.77) | 0.019 | |  |

Table S4 (Continued)

| **GWAS examining CRP as a binary outcome reflecting high immune response and chronic inflammation (CRP > 3.0 mg/L)** | | | | | | | | | | | | | | | | |
| --- | --- | --- | --- | --- | --- | --- | --- | --- | --- | --- | --- | --- | --- | --- | --- | --- |
|  | |  | **Stage 1**  **Adjustment for age and 10 PCs** | | | | | |  | **Stage 2**  **Adjustment for covariates***  **in addition to age and 10PCs** | | | | | | |
| **Subgroup** | | **Analytic method** | **HR¶** | **(95% CI)** | | | **p** | **p-het†** |  | **HR¶** | **(95% CI)** | | | **p** | **p-het†** | |
| **BMI** | **< 30** | Inverse-variance weighted | 1.15 | (0.67 | - | 1.96) | 0.521 | 0.469 |  | 1.10 | (0.63 | - | 1.92) | 0.653 | | 0.446 |
|  |  | Weighted median | 1.10 | (0.66 | - | 1.81) | 0.720 |  |  | 1.01 | (0.60 | - | 1.69) | 0.981 | |  |
|  |  | Penalized weighted median | 1.10 | (0.66 | - | 1.82) | 0.723 |  |  | 1.01 | (0.59 | - | 1.70) | 0.981 | |  |
|  |  | MR-Egger: slope | 4.06 | (0.14 | - | 118.56) | 0.278 |  |  | 5.26 | (0.24 | - | 113.81) | 0.184 | |  |
|  |  | intercept | 0.76 | (0.37 | - | 1.57) | 0.313 |  |  | 0.71 | (0.37 | - | 1.38) | 0.199 | |  |
|  | **≥ 30** | Inverse-variance weighted | 0.60 | (0.33 | - | 1.07) | 0.070 | 0.546 |  | 0.54 | (0.27 | - | 1.05) | 0.061 | | 0.422 |
|  |  | Weighted median | 0.57 | (0.32 | - | 1.02) | 0.058 |  |  | 0.50 | (0.26 | - | 0.94) | 0.033 | |  |
|  |  | Penalized weighted median | 0.57 | (0.31 | - | 1.04) | 0.068 |  |  | 0.50 | (0.26 | - | 0.95) | 0.034 | |  |
|  |  | MR-Egger: slope | 1.62 | (0.03 | - | 78.24) | 0.717 |  |  | 1.26 | (0.01 | - | 137.44) | 0.888 | |  |
|  |  | intercept | 0.80 | (0.34 | - | 1.87) | 0.465 |  |  | 0.83 | (0.30 | - | 2.31) | 0.599 | |  |
| **Cigarettes/d** | **< 15** | Inverse-variance weighted | 0.80 | (0.57 | - | 1.11) | 0.130 | 0.888 |  | 0.74 | (0.52 | - | 1.07) | 0.088 | | 0.862 |
|  |  | Weighted median | 0.80 | (0.47 | - | 1.36) | 0.409 |  |  | 0.75 | (0.44 | - | 1.31) | 0.313 | |  |
|  |  | Penalized weighted median | 0.80 | (0.47 | - | 1.37) | 0.413 |  |  | 0.75 | (0.44 | - | 1.30) | 0.312 | |  |
|  |  | MR-Egger: slope | 1.93 | (0.28 | - | 13.32) | 0.359 |  |  | 2.50 | (0.52 | - | 12.11) | 0.162 | |  |
|  |  | intercept | 0.82 | (0.54 | - | 1.25) | 0.235 |  |  | 0.77 | (0.55 | - | 1.08) | 0.089 | |  |
|  | **≥ 15** | Inverse-variance weighted | 0.93 | (0.34 | - | 2.56) | 0.859 | 0.020 |  | 0.90 | (0.31 | - | 2.59) | 0.795 | | 0.015 |
|  |  | Weighted median | 0.73 | (0.39 | - | 1.37) | 0.321 |  |  | 0.72 | (0.38 | - | 1.36) | 0.314 | |  |
|  |  | Penalized weighted median | 0.79 | (0.43 | - | 1.45) | 0.443 |  |  | 0.76 | (0.40 | - | 1.47) | 0.421 | |  |
|  |  | MR-Egger: slope | 4.61 | (0.00 | - | 4752.21) | 0.534 |  |  | 4.97 | (0.00 | - | 6730.57) | 0.530 | |  |
|  |  | intercept | 0.70 | (0.16 | - | 3.16) | 0.511 |  |  | 0.69 | (0.14 | - | 3.28) | 0.499 | |  |

Table S4 (Continued)

| **GWAS examining CRP as a binary outcome reflecting high immune response and chronic inflammation (CRP > 3.0 mg/L)** | | | | | | | | | | | | | | | | |
| --- | --- | --- | --- | --- | --- | --- | --- | --- | --- | --- | --- | --- | --- | --- | --- | --- |
|  | |  | **Stage 1**  **Adjustment for age and 10 PCs** | | | | | |  | **Stage 2**  **Adjustment for covariates***  **in addition to age and 10PCs** | | | | | | |
| **Subgroup** | | **Analytic method** | **HR¶** | **(95% CI)** | | | **p** | **p-het†** |  | **HR¶** | **(95% CI)** | | | **p** | **p-het†** | |
| **Depressive symptoms¥** | **< 0.06** | Inverse-variance weighted | 0.88 | (0.61 | - | 1.27) | 0.387 | 0.601 |  | 0.84 | (0.60 | - | 1.17) | 0.213 | | 0.699 |
|  |  | Weighted median | 0.87 | (0.59 | - | 1.28) | 0.489 |  |  | 0.79 | (0.53 | - | 1.18) | 0.240 | |  |
|  |  | Penalized weighted median | 0.87 | (0.58 | - | 1.31) | 0.508 |  |  | 0.79 | (0.53 | - | 1.17) | 0.233 | |  |
|  |  | MR-Egger: slope | 1.85 | (0.17 | - | 20.37) | 0.476 |  |  | 1.53 | (0.17 | - | 14.18) | 0.583 | |  |
|  |  | intercept | 0.85 | (0.51 | - | 1.43) | 0.391 |  |  | 0.88 | (0.54 | - | 1.42) | 0.443 | |  |
|  | **≥ 0.06** | Inverse-variance weighted | 2.65 | (0.52 | - | 13.48) | 0.171 | 0.684 |  | 14.60 | (0.58 | - | 368.22) | 0.082 | | 0.542 |
|  |  | Weighted median | 3.53 | (0.50 | - | 25.02) | 0.207 |  |  | 7.35 | (0.24 | - | 227.38) | 0.255 | |  |
|  |  | Penalized weighted median | 3.53 | (0.59 | - | 21.24) | 0.169 |  |  | 7.35 | (0.30 | - | 182.49) | 0.224 | |  |
|  |  | MR-Egger: slope | 681.83 | (0.60 | - | 7.76E+05) | 0.060 |  |  | NA | NA | - | NA | NA | |  |
|  |  | intercept | 0.30 | (0.07 | - | 1.35) | 0.084 |  |  | 2.02 | (0.01 | - | 295.20) | 0.684 | |  |

BMI, body mass index; CI, confidence interval; CRP, C-reactive protein; E+P, exogenous estrogen plus progestin; ER/PR, estrogen and progesterone receptor; GWAS, genome-wide association study; HER2/neu, human epidermal growth factor receptor 2; HR, hazard ratio; MET, metabolic equivalent; OC, oral contraceptive; PCs, principal components; SFA, saturated fatty acids; SNP, single-nucleotide polymorphism. WHR, waist-to-hip ratio.

***** Covariates adjusted in the analyses for the association between genome-wide SNPs and breast cancer risk include education; annual family income; family history of breast cancer; body mass index; waist-to-hip ratio; physical activity; depressive symptoms; number of cigarettes per day; dietary alcohol in g/day; % calories from SFA/day; age at menopause; duration of oral contraceptive use; and durations of exogenous estrogen [E]–only use and E plus progestin use; variables used to stratify were not included as covariates in the multivariate analysis.

¶ The Mendelian randomization estimate (except weighted/penalized weighted medians) was adjusted for a correlation between CRP phenotype and breast cancer risk within the same population.

† Heterogeneity in estimates among genome-wide SNPs was evaluated by Cochran’s Q test with fixed effects.

¥ Depression scales were estimated via a short form of the Center for Epidemiologic Studies Depression Scale.
