# Supplementary material for: Genetically Predicted C-Reactive Protein Associated With Postmenopausal Breast Cancer Risk: Interrelation With Estrogen and Cancer Molecular Subtypes Using Mendelian Randomization
Source: Front Oncol. 2021 Feb 3;10:630994. doi: 10.3389/fonc.2020.630994 (PMC7888276; doi:10.3389/fonc.2020.630994)
Supplement: Supplementary file 1 [file DataSheet_1.zip › TableS5_2020Nov18.docx]

Table S5. Mendelian randomization analysis: the effect of genetically predicted CRP phenotype on breast cancer risk by subtype

| **GWASs analyzing CRP as a continuous variable which was naturally log-transformed (mg/L)** | | | | | | | | | | | | | | | | | | | | | | | | | | | | |
| --- | --- | --- | --- | --- | --- | --- | --- | --- | --- | --- | --- | --- | --- | --- | --- | --- | --- | --- | --- | --- | --- | --- | --- | --- | --- | --- | --- | --- |
|  | **All SNPs** | | | | | | | | | | | | | |  | **After exclusion of pleiotropic SNPs** | | | | | | | | | | | | |
|  | **Stage 1**  **Adjustment for age and 10 PCs** | | | | | | |  | **Stage 2**  **Adjustment for covariates***  **in addition to age and 10PCs** | | | | | |  | **Stage 1**  **Adjustment for age and 10 PCs** | | | | | |  | **Stage 2**  **Adjustment for covariates***  **in addition to age and 10PCs** | | | | | |
| **Analysis method** | **HR** | **(95% CI)** | | | | **p** | **p-het†** |  | **HR** | **(95% CI)** | | | **p** | **p-het†** |  | **HR** | **(95% CI)** | | | **p** | **p-het†** |  | **HR** | **(95% CI)** | | | **p** | **p-het†** |
|  |  |  | | | |  |  |  |  |  | | |  |  |  |  |  | | |  |  |  |  |  | | |  |  |
| **Family history of breast cancer** | | | | | | | | | | | | | | | | | | | | | | | | | | | | |
| **No** |  |  |  |  |  | |  |  |  |  |  |  |  |  |  |  |  |  |  |  |  |  |  |  |  |  |  |  |
| Inverse-variance weighted | 0.90 | (0.63 | - | 1.30) | 0.581 | | 0.558 |  | 0.91 | (0.63 | - | 1.32) | 0.622 | 0.607 |  | 0.95 | (0.64 | - | 1.41) | 0.786 | 0.558 |  | 0.96 | (0.65 | - | 1.43) | 0.852 | 0.624 |
| Weighted median | 0.77 | (0.45 | - | 1.30) | 0.329 | |  |  | 0.71 | (0.41 | - | 1.21) | 0.205 |  |  | 0.97 | (0.54 | - | 1.74) | 0.911 |  |  | 0.95 | (0.54 | - | 1.66) | 0.853 |  |
| Penalized weighted median | 0.77 | (0.45 | - | 1.31) | 0.331 | |  |  | 0.71 | (0.40 | - | 1.26) | 0.239 |  |  | 0.97 | (0.54 | - | 1.73) | 0.914 |  |  | 0.95 | (0.53 | - | 1.72) | 0.871 |  |
| MR-Egger: slope | 0.91 | (0.52 | - | 1.57) | 0.723 | |  |  | 0.84 | (0.49 | - | 1.46) | 0.534 |  |  | 1.05 | (0.58 | - | 1.91) | 0.872 |  |  | 0.97 | (0.54 | - | 1.77) | 0.926 |  |
| intercept | 1.00 | (0.97 | - | 1.03) | 0.985 | |  |  | 1.01 | (0.97 | - | 1.04) | 0.693 |  |  | 0.99 | (0.96 | - | 1.03) | 0.647 |  |  | 1.00 | (0.97 | - | 1.03) | 0.967 |  |
| **Yes** |  |  |  |  |  | |  |  |  |  |  |  |  |  |  |  |  |  |  |  |  |  |  |  |  |  |  |  |
| Inverse-variance weighted | 1.26 | (0.65 | - | 2.41) | 0.488 | | 0.749 |  | 1.79 | (0.81 | - | 3.93) | 0.147 | 0.337 |  | 1.37 | (0.67 | - | 2.81) | 0.383 | 0.695 |  | 2.06 | (0.86 | - | 4.94) | 0.102 | 0.284 |
| Weighted median | 1.57 | (0.55 | - | 4.45) | 0.401 | |  |  | 2.84 | (0.90 | - | 8.95) | 0.074 |  |  | 1.83 | (0.63 | - | 5.33) | 0.269 |  |  | 2.98 | (0.89 | - | 10.01) | 0.077 |  |
| Penalized weighted median | 1.57 | (0.59 | - | 4.20) | 0.365 | |  |  | 2.85 | (0.92 | - | 8.83) | 0.069 |  |  | 1.84 | (0.62 | - | 5.42) | 0.269 |  |  | 2.99 | (0.86 | - | 10.34) | 0.084 |  |
| MR-Egger: slope | 2.86 | (1.14 | - | 7.16) | 0.026 | |  |  | 4.53 | (1.48 | - | 13.88) | 0.009 |  |  | 3.43 | (1.25 | - | 9.46) | 0.018 |  |  | 5.86 | (1.69 | - | 20.35) | 0.006 |  |
| intercept | 0.94 | (0.88 | - | 0.99) | 0.017 | |  |  | 0.93 | (0.87 | - | 0.99) | 0.027 |  |  | 0.93 | (0.88 | - | 0.99) | 0.017 |  |  | 0.92 | (0.86 | - | 0.99) | 0.027 |  |
|  | | | | | | | | | | | | | | | | | | | | | | | | | | | | |
| **Dietary alcohol** | | | | | | | | | | | | | | | | | | | | | | | | | | | | |
| **≤ 1 drink/d** |  |  |  |  |  | |  |  |  |  |  |  |  |  |  |  |  |  |  |  |  |  |  |  |  |  |  |  |
| Inverse-variance weighted | 0.84 | (0.60 | - | 1.18) | 0.314 | | 0.770 |  | 0.88 | (0.64 | - | 1.22) | 0.431 | 0.895 |  | 0.90 | (0.62 | - | 1.30) | 0.557 | 0.734 |  | 0.94 | (0.66 | - | 1.34) | 0.730 | 0.870 |
| Weighted median | 0.82 | (0.49 | - | 1.40) | 0.471 | |  |  | 0.88 | (0.52 | - | 1.47) | 0.623 |  |  | 0.88 | (0.51 | - | 1.51) | 0.640 |  |  | 0.90 | (0.52 | - | 1.58) | 0.719 |  |
| Penalized weighted median | 0.82 | (0.49 | - | 1.40) | 0.472 | |  |  | 0.88 | (0.52 | - | 1.48) | 0.628 |  |  | 0.89 | (0.50 | - | 1.56) | 0.675 |  |  | 0.90 | (0.52 | - | 1.57) | 0.714 |  |
| MR-Egger: slope | 1.10 | (0.67 | - | 1.80) | 0.713 | |  |  | 1.08 | (0.67 | - | 1.74) | 0.755 |  |  | 1.24 | (0.72 | - | 2.14) | 0.428 |  |  | 1.23 | (0.73 | - | 2.07) | 0.434 |  |
| intercept | 0.98 | (0.95 | - | 1.01) | 0.155 | |  |  | 0.98 | (0.96 | - | 1.01) | 0.254 |  |  | 0.98 | (0.95 | - | 1.01) | 0.111 |  |  | 0.98 | (0.95 | - | 1.01) | 0.173 |  |
| **> 1 drink/d** |  |  |  |  |  | |  |  |  |  |  |  |  |  |  |  |  |  |  |  |  |  |  |  |  |  |  |  |
| Inverse-variance weighted | 2.03 | (0.98 | - | 4.20) | 0.055 | | 0.757 |  | 2.15 | (0.92 | - | 5.03) | 0.075 | 0.513 |  | 1.83 | (0.87 | - | 3.84) | 0.110 | 0.873 |  | 2.01 | (0.82 | - | 4.94) | 0.123 | 0.615 |
| Weighted median | 1.56 | (0.54 | - | 4.51) | 0.412 | |  |  | 1.51 | (0.44 | - | 5.14) | 0.513 |  |  | 1.61 | (0.50 | - | 5.23) | 0.429 |  |  | 1.65 | (0.46 | - | 5.93) | 0.443 |  |
| Penalized weighted median | 1.57 | (0.51 | - | 4.79) | 0.430 | |  |  | 1.52 | (0.47 | - | 4.95) | 0.486 |  |  | 1.64 | (0.52 | - | 5.16) | 0.402 |  |  | 1.67 | (0.45 | - | 6.14) | 0.443 |  |
| MR-Egger: slope | 1.48 | (0.50 | - | 4.37) | 0.476 | |  |  | 1.80 | (0.51 | - | 6.40) | 0.356 |  |  | 1.57 | (0.51 | - | 4.82) | 0.426 |  |  | 2.07 | (0.53 | - | 8.04) | 0.289 |  |
| intercept | 1.03 | (0.96 | - | 1.09) | 0.427 | |  |  | 1.01 | (0.94 | - | 1.09) | 0.704 |  |  | 1.01 | (0.95 | - | 1.08) | 0.716 |  |  | 1.00 | (0.93 | - | 1.08) | 0.959 |  |

Table S5 (Continued)

| **GWASs analyzing CRP as a continuous variable which was naturally log-transformed (mg/L)** | | | | | | | | | | | | | | | | | | | | | | | | | | | | |
| --- | --- | --- | --- | --- | --- | --- | --- | --- | --- | --- | --- | --- | --- | --- | --- | --- | --- | --- | --- | --- | --- | --- | --- | --- | --- | --- | --- | --- |
|  | **All SNPs** | | | | | | | | | | | | | |  | **After exclusion of pleiotropic SNPs** | | | | | | | | | | | | |
|  | **Stage 1**  **Adjustment for age and 10 PCs** | | | | | | |  | **Stage 2**  **Adjustment for covariates***  **in addition to age and 10PCs** | | | | | |  | **Stage 1**  **Adjustment for age and 10 PCs** | | | | | |  | **Stage 2**  **Adjustment for covariates***  **in addition to age and 10PCs** | | | | | |
| **Analysis method** | **HR** | **(95% CI)** | | | | **p** | **p-het†** |  | **HR** | **(95% CI)** | | | **p** | **p-het†** |  | **HR** | **(95% CI)** | | | **p** | **p-het†** |  | **HR** | **(95% CI)** | | | **p** | **p-het†** |
|  |  |  |  |  |  | |  |  |  |  |  |  |  |  |  |  |  |  |  |  |  |  |  |  |  |  |  |  |
| **% calories from SFA** | | | | | | | | | | | | | | | | | | | | | | | | | | | | |
| **< 9.0%** |  |  |  |  |  | |  |  |  |  |  |  |  |  |  |  |  |  |  |  |  |  |  |  |  |  |  |  |
| Inverse-variance weighted | 1.67 | (0.79 | - | 3.52) | 0.176 | | 0.126 |  | 1.71 | (0.78 | - | 3.73) | 0.176 | 0.137 |  | 1.68 | (0.75 | - | 3.80) | 0.205 | 0.101 |  | 1.78 | (0.76 | - | 4.17) | 0.180 | 0.121 |
| Weighted median | 1.39 | (0.53 | - | 3.67) | 0.505 | |  |  | 1.29 | (0.47 | - | 3.55) | 0.623 |  |  | 1.41 | (0.49 | - | 4.05) | 0.526 |  |  | 1.29 | (0.43 | - | 3.87) | 0.651 |  |
| Penalized weighted median | 1.39 | (0.54 | - | 3.60) | 0.493 | |  |  | 1.29 | (0.46 | - | 3.61) | 0.629 |  |  | 1.41 | (0.51 | - | 3.89) | 0.506 |  |  | 1.29 | (0.44 | - | 3.78) | 0.643 |  |
| MR-Egger: slope | 1.58 | (0.52 | - | 4.81) | 0.412 | |  |  | 1.79 | (0.56 | - | 5.73) | 0.323 |  |  | 1.81 | (0.54 | - | 6.12) | 0.331 |  |  | 2.18 | (0.61 | - | 7.82) | 0.226 |  |
| intercept | 1.00 | (0.94 | - | 1.07) | 0.897 | |  |  | 1.00 | (0.93 | - | 1.07) | 0.917 |  |  | 0.99 | (0.93 | - | 1.07) | 0.870 |  |  | 0.99 | (0.92 | - | 1.06) | 0.669 |  |
| **≥ 9.0%** |  |  |  |  |  | |  |  |  |  |  |  |  |  |  |  |  |  |  |  |  |  |  |  |  |  |  |  |
| Inverse-variance weighted | 0.83 | (0.59 | - | 1.17) | 0.279 | | 0.797 |  | 0.86 | (0.61 | - | 1.21) | 0.373 | 0.843 |  | 0.88 | (0.61 | - | 1.28) | 0.509 | 0.810 |  | 0.93 | (0.64 | - | 1.34) | 0.681 | 0.881 |
| Weighted median | 0.83 | (0.46 | - | 1.48) | 0.517 | |  |  | 1.00 | (0.57 | - | 1.74) | 0.989 |  |  | 0.93 | (0.51 | - | 1.68) | 0.803 |  |  | 1.07 | (0.60 | - | 1.92) | 0.821 |  |
| Penalized weighted median | 0.83 | (0.47 | - | 1.46) | 0.508 | |  |  | 1.00 | (0.56 | - | 1.76) | 0.993 |  |  | 0.93 | (0.51 | - | 1.70) | 0.810 |  |  | 1.07 | (0.61 | - | 1.88) | 0.813 |  |
| MR-Egger: slope | 1.07 | (0.64 | - | 1.78) | 0.805 | |  |  | 1.08 | (0.65 | - | 1.79) | 0.771 |  |  | 1.26 | (0.73 | - | 2.17) | 0.410 |  |  | 1.28 | (0.75 | - | 2.20) | 0.356 |  |
| intercept | 0.98 | (0.95 | - | 1.01) | 0.186 | |  |  | 0.98 | (0.95 | - | 1.01) | 0.228 |  |  | 0.97 | (0.94 | - | 1.00) | 0.090 |  |  | 0.98 | (0.95 | - | 1.01) | 0.109 |  |
|  |  |  |  |  |  | |  |  |  |  |  |  |  |  |  |  |  |  |  |  |  |  |  |  |  |  |  |  |
| **MET** | | | | | | | | | | | | | | | | | | | | | | | | | | | | |
| **≥ 10** |  |  |  |  |  | |  |  |  |  |  |  |  |  |  |  |  |  |  |  |  |  |  |  |  |  |  |  |
| Inverse-variance weighted | 1.39 | (0.86 | - | 2.25) | 0.181 | | 0.725 |  | 1.39 | (0.86 | - | 2.27) | 0.179 | 0.763 |  | 1.39 | (0.84 | - | 2.30) | 0.198 | 0.817 |  | 1.42 | (0.85 | - | 2.39) | 0.177 | 0.809 |
| Weighted median | 1.26 | (0.62 | - | 2.58) | 0.527 | |  |  | 1.29 | (0.63 | - | 2.68) | 0.488 |  |  | 1.34 | (0.65 | - | 2.79) | 0.428 |  |  | 1.32 | (0.61 | - | 2.87) | 0.476 |  |
| Penalized weighted median | 1.26 | (0.65 | - | 2.46) | 0.497 | |  |  | 1.29 | (0.63 | - | 2.66) | 0.485 |  |  | 1.34 | (0.63 | - | 2.86) | 0.445 |  |  | 1.32 | (0.61 | - | 2.86) | 0.474 |  |
| MR-Egger: slope | 1.50 | (0.73 | - | 3.08) | 0.264 | |  |  | 1.46 | (0.70 | - | 3.03) | 0.303 |  |  | 1.69 | (0.79 | - | 3.59) | 0.170 |  |  | 1.64 | (0.76 | - | 3.58) | 0.206 |  |
| intercept | 0.99 | (0.95 | - | 1.04) | 0.766 | |  |  | 1.00 | (0.95 | - | 1.04) | 0.861 |  |  | 0.99 | (0.94 | - | 1.03) | 0.486 |  |  | 0.99 | (0.95 | - | 1.03) | 0.621 |  |
| **< 10** |  |  |  |  |  | |  |  |  |  |  |  |  |  |  |  |  |  |  |  |  |  |  |  |  |  |  |  |
| Inverse-variance weighted | 0.75 | (0.48 | - | 1.18) | 0.209 | | 0.315 |  | 0.83 | (0.53 | - | 1.30) | 0.414 | 0.390 |  | 0.81 | (0.49 | - | 1.33) | 0.392 | 0.213 |  | 0.93 | (0.56 | - | 1.53) | 0.760 | 0.298 |
| Weighted median | 0.63 | (0.32 | - | 1.21) | 0.162 | |  |  | 0.70 | (0.36 | - | 1.36) | 0.292 |  |  | 0.63 | (0.31 | - | 1.29) | 0.208 |  |  | 0.71 | (0.34 | - | 1.50) | 0.370 |  |
| Penalized weighted median | 0.63 | (0.33 | - | 1.19) | 0.151 | |  |  | 0.70 | (0.35 | - | 1.39) | 0.306 |  |  | 0.63 | (0.32 | - | 1.27) | 0.197 |  |  | 0.72 | (0.35 | - | 1.47) | 0.362 |  |
| MR-Egger: slope | 0.90 | (0.46 | - | 1.75) | 0.748 | |  |  | 0.93 | (0.48 | - | 1.81) | 0.824 |  |  | 1.02 | (0.48 | - | 2.17) | 0.954 |  |  | 1.11 | (0.53 | - | 2.36) | 0.773 |  |
| intercept | 0.99 | (0.95 | - | 1.03) | 0.477 | |  |  | 0.99 | (0.95 | - | 1.03) | 0.660 |  |  | 0.98 | (0.94 | - | 1.03) | 0.399 |  |  | 0.99 | (0.95 | - | 1.03) | 0.510 |  |

Table S5 (Continued)

| **GWASs analyzing CRP as a continuous variable which was naturally log-transformed (mg/L)** | | | | | | | | | | | | | | | | | | | | | | | | | | | | | |
| --- | --- | --- | --- | --- | --- | --- | --- | --- | --- | --- | --- | --- | --- | --- | --- | --- | --- | --- | --- | --- | --- | --- | --- | --- | --- | --- | --- | --- | --- |
|  | **All SNPs** | | | | | | | | | | | | | |  | **After exclusion of pleiotropic SNPs** | | | | | | | | | | | | | |
|  | **Stage 1**  **Adjustment for age and 10 PCs** | | | | | | |  | **Stage 2**  **Adjustment for covariates***  **in addition to age and 10PCs** | | | | | |  | **Stage 1**  **Adjustment for age and 10 PCs** | | | | | |  | **Stage 2**  **Adjustment for covariates***  **in addition to age and 10PCs** | | | | | | |
| **Analysis method** | **HR** | **(95% CI)** | | | | **p** | **p-het†** |  | **HR** | **(95% CI)** | | | **p** | **p-het†** |  | **HR** | **(95% CI)** | | | **p** | **p-het†** |  | **HR** | **(95% CI)** | | | **p** | **p-het†** |  |
|  |  |  |  |  |  | |  |  |  |  |  |  |  |  |  |  |  |  |  |  |  |  |  |  |  |  |  |  |  |
| **E-only use** | | | | | | | | | | | | | | | | | | | | | | | | | | | | | |
| **Nonusers** |  |  |  |  |  | |  |  |  |  |  |  |  |  |  |  |  |  |  |  |  |  |  |  |  |  |  |  |  |
| Inverse-variance weighted | 1.03 | (0.71 | - | 1.50) | 0.874 | | 0.480 |  | 1.00 | (0.69 | - | 1.45) | 0.990 | 0.612 |  | 1.06 | (0.70 | - | 1.61) | 0.774 | 0.415 |  | 1.06 | (0.71 | - | 1.60) | 0.764 | 0.524 |  |
| Weighted median | 1.03 | (0.60 | - | 1.77) | 0.923 | |  |  | 0.93 | (0.53 | - | 1.64) | 0.812 |  |  | 1.10 | (0.63 | - | 1.94) | 0.731 |  |  | 1.01 | (0.56 | - | 1.80) | 0.982 |  |  |
| Penalized weighted median | 1.03 | (0.61 | - | 1.73) | 0.919 | |  |  | 0.94 | (0.53 | - | 1.66) | 0.819 |  |  | 1.11 | (0.62 | - | 1.97) | 0.736 |  |  | 1.01 | (0.55 | - | 1.83) | 0.981 |  |  |
| MR-Egger: slope | 1.08 | (0.62 | - | 1.90) | 0.777 | |  |  | 1.06 | (0.61 | - | 1.83) | 0.845 |  |  | 1.21 | (0.65 | - | 2.26) | 0.540 |  |  | 1.22 | (0.66 | - | 2.25) | 0.523 |  |  |
| intercept | 1.00 | (0.96 | - | 1.03) | 0.811 | |  |  | 1.00 | (0.96 | - | 1.03) | 0.799 |  |  | 0.99 | (0.96 | - | 1.03) | 0.570 |  |  | 0.99 | (0.96 | - | 1.03) | 0.555 |  |  |
| **<5 Years** |  |  |  |  |  | |  |  |  |  |  |  |  |  |  |  |  |  |  |  |  |  |  |  |  |  |  |  |  |
| Inverse-variance weighted | 1.16 | (0.31 | - | 4.33) | 0.825 | | 0.023 |  | 0.00 | (0.00 | - | 0.59) | 0.047 | 0.000 |  | 1.42 | (0.36 | - | 5.68) | 0.614 | 0.023 |  | 0.00 | (0.00 | - | 1.39) | 0.052 | 0.000 |  |
| Weighted median | 2.89 | (0.45 | - | 18.56) | 0.264 | |  |  | 0.39 | (0.03 | - | 4.76) | 0.457 |  |  | 2.98 | (0.41 | - | 21.49) | 0.278 |  |  | 0.91 | (0.06 | - | 13.40) | 0.945 |  |  |
| Penalized weighted median | 0.33 | (0.05 | - | 2.36) | 0.271 | |  |  | NA | NA | - | NA | NA |  |  | 0.46 | (0.07 | - | 3.18) | 0.429 |  |  | NA | NA | - | NA | NA |  |  |
| MR-Egger: slope | 5.91 | (0.93 | - | 37.48) | 0.059 | |  |  | NA | NA | - | NA | NA |  |  | 7.18 | (1.05 | - | 49.13) | 0.045 |  |  | NA | NA | - | NA | NA |  |  |
| intercept | 0.87 | (0.78 | - | 0.98) | 0.019 | |  |  | 0.01 | (0.00 | - | 0.18) | 0.002 |  |  | 0.87 | (0.78 | - | 0.98) | 0.023 |  |  | 0.01 | (0.00 | - | 0.22) | 0.003 |  |  |
| **≥ 5 years** |  |  |  |  |  | |  |  |  |  |  |  |  |  |  |  |  |  |  |  |  |  |  |  |  |  |  |  |  |
| Inverse-variance weighted | 1.29 | (0.48 | - | 3.48) | 0.603 | | 0.305 |  | 1.33 | (0.39 | - | 4.60) | 0.645 | 0.069 |  | 1.06 | (0.37 | - | 3.05) | 0.906 | 0.433 |  | 0.92 | (0.25 | - | 3.35) | 0.894 | 0.188 |  |
| Weighted median | 1.10 | (0.28 | - | 4.30) | 0.893 | |  |  | 1.74 | (0.37 | - | 8.25) | 0.488 |  |  | 0.97 | (0.22 | - | 4.30) | 0.967 |  |  | 1.49 | (0.26 | - | 8.41) | 0.654 |  |  |
| Penalized weighted median | 1.09 | (0.25 | - | 4.70) | 0.913 | |  |  | 1.73 | (0.38 | - | 7.80) | 0.475 |  |  | 0.97 | (0.20 | - | 4.56) | 0.964 |  |  | 1.50 | (0.26 | - | 8.64) | 0.653 |  |  |
| MR-Egger: slope | 2.32 | (0.53 | - | 10.13) | 0.258 | |  |  | 4.46 | (0.73 | - | 27.30) | 0.104 |  |  | 1.88 | (0.38 | - | 9.25) | 0.433 |  |  | 3.19 | (0.46 | - | 21.88) | 0.233 |  |  |
| intercept | 0.96 | (0.88 | - | 1.04) | 0.291 | |  |  | 0.91 | (0.82 | - | 1.01) | 0.078 |  |  | 0.96 | (0.88 | - | 1.05) | 0.347 |  |  | 0.91 | (0.82 | - | 1.02) | 0.091 |  |  |
| **10+ Years** |  |  |  |  |  | |  |  |  |  |  |  |  |  |  |  |  |  |  |  |  |  |  |  |  |  |  |  |  |
| Inverse-variance weighted | 1.72 | (0.57 | - | 5.16) | 0.329 | | 0.445 |  | NA | NA | - | NA | NA | 0.000 |  | 1.28 | (0.40 | - | 4.10) | 0.668 | 0.591 |  | NA | NA | - | NA | NA | 0.000 |  |
| Weighted median | 1.29 | (0.24 | - | 7.10) | 0.767 | |  |  | NA | NA | - | NA | NA |  |  | 1.26 | (0.20 | - | 7.95) | 0.804 |  |  | NA | NA | - | NA | NA |  |  |
| Penalized weighted median | 1.29 | (0.25 | - | 6.67) | 0.758 | |  |  | NA | NA | - | NA | NA |  |  | 1.26 | (0.21 | - | 7.54) | 0.799 |  |  | NA | NA | - | NA | NA |  |  |
| MR-Egger: slope | 5.05 | (1.02 | - | 25.02) | 0.047 | |  |  | NA | NA | - | NA | NA |  |  | 3.68 | (0.66 | - | 20.47) | 0.133 |  |  | NA | NA | - | NA | NA |  |  |
| intercept | 0.92 | (0.84 | - | 1.01) | 0.073 | |  |  | 0.13 | (0.01 | - | 3.24) | 0.209 |  |  | 0.93 | (0.84 | - | 1.02) | 0.105 |  |  | 0.16 | (0.01 | - | 4.87) | 0.285 |  |  |

Table S5 (Continued)

| **GWASs analyzing CRP as a continuous variable which was naturally log-transformed (mg/L)** | | | | | | | | | | | | | | | | | | | | | | | | | | | | | | | | | | | |  |
| --- | --- | --- | --- | --- | --- | --- | --- | --- | --- | --- | --- | --- | --- | --- | --- | --- | --- | --- | --- | --- | --- | --- | --- | --- | --- | --- | --- | --- | --- | --- | --- | --- | --- | --- | --- | --- |
|  | **All SNPs** | | | | | | | | | | | | | | | |  | | **After exclusion of pleiotropic SNPs** | | | | | | | | | | | | | | | | |  |
|  | **Stage 1**  **Adjustment for age and 10 PCs** | | | | | | |  | | **Stage 2**  **Adjustment for covariates***  **in addition to age and 10PCs** | | | | | | |  | | **Stage 1**  **Adjustment for age and 10 PCs** | | | | | | | |  | | **Stage 2**  **Adjustment for covariates***  **in addition to age and 10PCs** | | | | | | |  |
| **Analysis method** | **HR** | **(95% CI)** | | | **p** | **p-het†** | |  | | **HR** | | **(95% CI)** | | | **p** | **p-het†** | |  | | **HR** | | **(95% CI)** | | | **p** | **p-het†** | |  | | **HR** | **(95% CI)** | | | **p** | **p-het†** | |
| **E+P use** | | | | | | | | | | | | | | | | | | | | | | | | | | | | | | | | | | | |  |
| **Nonusers** |  |  |  |  |  | |  | |  | |  |  |  |  |  |  | |  |  | |  | |  |  |  |  | |  | |  |  |  |  |  |  | |
| Inverse-variance weighted | 0.90 | (0.62 | - | 1.31) | 0.577 | | 0.461 | |  | | 0.95 | (0.66 | - | 1.37) | 0.787 | 0.636 | |  | 0.93 | | (0.61 | | - | 1.42) | 0.744 | 0.344 | |  | | 1.00 | (0.67 | - | 1.51) | 0.989 | 0.518 | |
| Weighted median | 1.00 | (0.59 | - | 1.69) | 0.991 | |  | |  | | 1.05 | (0.61 | - | 1.81) | 0.865 |  | |  | 1.03 | | (0.58 | | - | 1.82) | 0.918 |  | |  | | 1.06 | (0.58 | - | 1.93) | 0.846 |  | |
| Penalized weighted median | 1.00 | (0.60 | - | 1.66) | 0.994 | |  | |  | | 1.05 | (0.61 | - | 1.80) | 0.864 |  | |  | 1.03 | | (0.59 | | - | 1.82) | 0.914 |  | |  | | 1.06 | (0.61 | - | 1.85) | 0.827 |  | |
| MR-Egger: slope | 1.02 | (0.58 | - | 1.78 | 0.951 | |  | |  | | 1.08 | (0.63 | - | 1.85) | 0.786 |  | |  | 1.13 | | (0.61 | | - | 2.13) | 0.689 |  | |  | | 1.22 | (0.66 | - | 2.24) | 0.518 |  | |
| intercept | 0.99 | (0.96 | - | 1.02) | 0.555 | |  | |  | | 0.99 | (0.96 | - | 1.02) | 0.540 |  | |  | 0.99 | | (0.95 | | - | 1.02) | 0.407 |  | |  | | 0.99 | (0.95 | - | 1.02) | 0.392 |  | |
| **<5 Years** |  |  |  |  |  | |  | |  | |  |  |  |  |  |  | |  |  | |  | |  |  |  |  | |  | |  |  |  |  |  |  | |
| Inverse-variance weighted | 1.45 | (0.41 | - | 5.10 | 0.561 | | 0.020 | |  | | 0.00 | (0.00 | - | 2.3E+06) | 0.406 | 0.000 | |  | 1.06 | | (0.26 | | - | 4.40) | 0.936 | 0.014 | |  | | 0.00 | (0.00 | - | 8.3E+06) | 0.377 | 0.000 | |
| Weighted median | 2.86 | (0.59 | - | 13.79) | 0.191 | |  | |  | | 0.70 | (0.08 | - | 5.74) | 0.735 |  | |  | 1.92 | | (0.31 | | - | 11.77) | 0.481 |  | |  | | 0.53 | (0.04 | - | 7.13) | 0.633 |  | |
| Penalized weighted median | 3.06 | (0.65 | - | 14.34) | 0.157 | |  | |  | | 0.01 | (0.00 | - | 1.03) | 0.051 |  | |  | 1.99 | | (0.33 | | - | 12.14) | 0.457 |  | |  | | 0.00 | (0.00 | - | 0.42) | 0.022 |  | |
| MR-Egger: slope | 3.04 | (0.47 | - | 19.54) | 0.236 | |  | |  | | NA | NA | - | NA | NA |  | |  | 2.20 | | (0.26 | | - | 18.67) | 0.463 |  | |  | | NA | NA | - | NA | NA |  | |
| intercept | 0.94 | (0.84 | - | 1.05) | 0.281 | |  | |  | | 0.17 | (0.02 | - | 1.42) | 0.100 |  | |  | 0.95 | | (0.84 | | - | 1.07) | 0.362 |  | |  | | 0.16 | (0.02 | - | 1.61) | 0.117 |  | |
| **≥ 5 years** |  |  |  |  |  | |  | |  | |  |  |  |  |  |  | |  |  | |  | |  |  |  |  | |  | |  |  |  |  |  |  | |
| Inverse-variance weighted | 1.28 | (0.47 | - | 3.44) | 0.623 | | 0.763 | |  | | NA | NA | - | NA | NA | 0.000 | |  | 1.96 | | (0.71 | | - | 5.36) | 0.188 | 0.872 | |  | | NA | NA | - | NA | NA | 0.000 | |
| Weighted median | 1.79 | (0.37 | - | 8.75) | 0.470 | |  | |  | | NA | NA | - | NA | NA |  | |  | 2.36 | | (0.46 | | - | 12.05) | 0.300 |  | |  | | NA | NA | - | NA | NA |  | |
| Penalized weighted median | 1.77 | (0.36 | - | 8.82) | 0.485 | |  | |  | | NA | NA | - | NA | NA |  | |  | 2.36 | | (0.44 | | - | 12.67) | 0.318 |  | |  | | NA | NA | - | NA | NA |  | |
| MR-Egger: slope | 3.64 | (0.88 | - | 15.13) | 0.075 | |  | |  | | 0.19 | (0.00 | - | 53.32) | 0.556 |  | |  | 6.90 | | (1.67 | | - | 28.50) | 0.009 |  | |  | | 0.74 | (0.01 | - | 90.84) | 0.901 |  | |
| intercept | 0.92 | (0.84 | - | 1.00) | 0.050 | |  | |  | | 0.57 | (0.40 | - | 0.80) | 0.002 |  | |  | 0.90 | | (0.83 | | - | 0.98) | 0.018 |  | |  | | 0.58 | (0.43 | - | 0.77) | 0.000 |  | |
| **10+ Years** |  |  |  |  |  | |  | |  | |  |  |  |  |  |  | |  |  | |  | |  |  |  |  | |  | |  |  |  |  |  |  | |
| Inverse-variance weighted | 0.76 | (0.14 | - | 4.12) | 0.742 | | 0.775 | |  | | NA | NA | - | NA | NA | NA | |  | 0.85 | | (0.14 | | - | 5.08) | 0.852 | 0.817 | |  | | 0.00 | (0.00 | - | 5.5E+43) | 0.593 | 0.000 | |
| Weighted median | 1.32 | (0.07 | - | 25.51) | 0.852 | |  | |  | | 0.07 | (0.00 | - | 6.46) | 0.245 |  | |  | 1.87 | | (0.09 | | - | 37.74) | 0.685 |  | |  | | 4.80 | (0.02 | - | 1.1E+03) | 0.568 |  | |
| Penalized weighted median | 1.33 | (0.07 | - | 25.63) | 0.848 | |  | |  | | 0.00 | (0.00 | - | 6.05) | 0.120 |  | |  | 1.90 | | (0.08 | | - | 44.04) | 0.689 |  | |  | | NA | NA | - | NA | NA |  | |
| MR-Egger: slope | 4.95 | (0.42 | - | 58.43) | 0.200 | |  | |  | | NA | NA | - | NA | NA |  | |  | 8.63 | | (0.67 | | - | 111.26) | 0.097 |  | |  | | NA | NA | - | NA | NA |  | |
| intercept | 0.86 | (0.74 | - | 1.00) | 0.045 | |  | |  | | 0.00 | (0.00 | - | 149.13) | 0.290 |  | |  | 0.84 | | (0.73 | | - | 0.97) | 0.018 |  | |  | | 0.00 | (0.00 | - | 274.62) | 0.305 |  | |

Table S5 (Continued)

| **GWASs analyzing CRP as a continuous variable which was naturally log-transformed (mg/L)** | | | | | | | | | | | | | | | | | | | | | | | | | | | | | | | | | |  |
| --- | --- | --- | --- | --- | --- | --- | --- | --- | --- | --- | --- | --- | --- | --- | --- | --- | --- | --- | --- | --- | --- | --- | --- | --- | --- | --- | --- | --- | --- | --- | --- | --- | --- | --- |
|  | **All SNPs** | | | | | | | | | | | | | | | | |  | | **After exclusion of pleiotropic SNPs** | | | | | | | | | | | | | |  |
|  | **Stage 1**  **Adjustment for age and 10 PCs** | | | | | | |  | | **Stage 2**  **Adjustment for covariates***  **in addition to age and 10PCs** | | | | | | | |  | | **Stage 1**  **Adjustment for age and 10 PCs** | | | | | | |  | **Stage 2**  **Adjustment for covariates***  **in addition to age and 10PCs** | | | | | | |
| **Analysis method** | **HR** | **(95% CI)** | | | **p** | **p-het†** | |  | | **HR** | | **(95% CI)** | | | | **p** | **p-het†** | |  | | **HR** | **(95% CI)** | | | **p** | **p-het†** |  | **HR** | **(95% CI)** | | | **p** | **p-het†** | |
| **WHR** | | | | | | | | | | | | | | | | | | | | | | | | | | | | | | | | | |  |
| **≤ 0.85** |  |  |  |  |  | |  | |  | |  | |  |  |  |  |  | |  | |  |  |  |  |  |  |  |  |  |  |  |  |  | |
| Inverse-variance weighted | 0.87 | (0.60 | - | 1.26) | 0.450 | | 0.825 | |  | | 0.86 | | (0.60 | - | 1.24) | 0.418 | 0.905 | |  | | 0.93 | (0.63 | - | 1.38) | 0.715 | 0.824 |  | 0.96 | (0.66 | - | 1.40) | 0.828 | 0.928 | |
| Weighted median | 0.84 | (0.47 | - | 1.49) | 0.545 | |  | |  | | 0.99 | | (0.55 | - | 1.76) | 0.960 |  | |  | | 0.88 | (0.48 | - | 1.61) | 0.681 |  |  | 0.99 | (0.53 | - | 1.85) | 0.970 |  | |
| Penalized weighted median | 0.84 | (0.47 | - | 1.48) | 0.544 | |  | |  | | 0.99 | | (0.55 | - | 1.78) | 0.961 |  | |  | | 0.88 | (0.48 | - | 1.62) | 0.687 |  |  | 0.99 | (0.54 | - | 1.82) | 0.969 |  | |
| MR-Egger: slope | 0.88 | (0.51 | - | 1.53) | 0.655 | |  | |  | | 0.90 | | (0.52 | - | 1.53) | 0.683 |  | |  | | 0.99 | (0.54 | - | 1.81) | 0.981 |  |  | 1.06 | (0.60 | - | 1.88) | 0.844 |  | |
| intercept | 1.00 | (0.97 | - | 1.03) | 0.937 | |  | |  | | 1.00 | | (0.97 | - | 1.03) | 0.856 |  | |  | | 1.00 | (0.96 | - | 1.03) | 0.770 |  |  | 0.99 | (0.96 | - | 1.03) | 0.647 |  | |
| **> 0.85** |  |  |  |  |  | |  | |  | |  | |  |  |  |  |  | |  | |  |  |  |  |  |  |  |  |  |  |  |  |  | |
| Inverse-variance weighted | 1.32 | (0.73 | - | 2.41) | 0.351 | | 0.279 | |  | | 1.42 | | (0.77 | - | 2.62) | 0.253 | 0.311 | |  | | 1.32 | (0.69 | - | 2.52) | 0.390 | 0.292 |  | 1.46 | (0.76 | - | 2.83) | 0.251 | 0.338 | |
| Weighted median | 1.34 | (0.57 | - | 3.16) | 0.499 | |  | |  | | 1.80 | | (0.72 | - | 4.53) | 0.211 |  | |  | | 1.43 | (0.63 | - | 3.27) | 0.394 |  |  | 1.86 | (0.73 | - | 4.73) | 0.194 |  | |
| Penalized weighted median | 1.33 | (0.59 | - | 3.01) | 0.489 | |  | |  | | 1.80 | | (0.73 | - | 4.45) | 0.205 |  | |  | | 1.42 | (0.61 | - | 3.34) | 0.419 |  |  | 1.85 | (0.77 | - | 4.48) | 0.170 |  | |
| MR-Egger: slope | 2.00 | (0.83 | - | 4.80) | 0.119 | |  | |  | | 2.06 | | (0.84 | - | 5.06) | 0.113 |  | |  | | 2.36 | (0.92 | - | 6.03) | 0.072 |  |  | 2.54 | (0.97 | - | 6.64) | 0.058 |  | |
| intercept | 0.97 | (0.92 | - | 1.02) | 0.204 | |  | |  | | 0.97 | | (0.92 | - | 1.02) | 0.266 |  | |  | | 0.96 | (0.91 | - | 1.01) | 0.099 |  |  | 0.96 | (0.91 | - | 1.01) | 0.127 |  | |
|  | | | | | | | | | | | | | | | | | | | | | | | | | | | | | | | | | |  |
| **BMI** | | | | | | | | | | | | | | | | | | | | | | | | | | | | | | | | | |  |
| **< 30** |  |  |  |  |  | |  | |  | |  | |  |  |  |  |  | |  | |  |  |  |  |  |  |  |  |  |  |  |  |  | |
| Inverse-variance weighted | 0.96 | (0.63 | - | 1.44) | 0.823 | | 0.610 | |  | | 0.98 | | (0.67 | - | 1.43) | 0.925 | 0.901 | |  | | 1.05 | (0.68 | - | 1.60) | 0.835 | 0.740 |  | 1.11 | (0.76 | - | 1.63) | 0.578 | 0.959 | |
| Weighted median | 0.94 | (0.52 | - | 1.70) | 0.846 | |  | |  | | 1.12 | | (0.61 | - | 2.05) | 0.720 |  | |  | | 0.97 | (0.54 | - | 1.75) | 0.917 |  |  | 1.15 | (0.63 | - | 2.08) | 0.652 |  | |
| Penalized weighted median | 0.95 | (0.52 | - | 1.73) | 0.853 | |  | |  | | 1.15 | | (0.63 | - | 2.10) | 0.658 |  | |  | | 0.97 | (0.52 | - | 1.80) | 0.925 |  |  | 1.15 | (0.63 | - | 2.10) | 0.658 |  | |
| MR-Egger: slope | 0.98 | (0.53 | - | 1.80) | 0.933 | |  | |  | | 1.00 | | (0.57 | - | 1.75) | 0.995 |  | |  | | 1.26 | (0.66 | - | 2.37) | 0.477 |  |  | 1.34 | (0.75 | - | 2.37) | 0.316 |  | |
| intercept | 1.00 | (0.96 | - | 1.04) | 0.929 | |  | |  | | 1.00 | | (0.97 | - | 1.03) | 0.939 |  | |  | | 0.99 | (0.95 | - | 1.02) | 0.439 |  |  | 0.99 | (0.96 | - | 1.02) | 0.394 |  | |
| **≥ 30** |  |  |  |  |  | |  | |  | |  | |  |  |  |  |  | |  | |  |  |  |  |  |  |  |  |  |  |  |  |  | |
| Inverse-variance weighted | 1.09 | (0.62 | - | 1.93) | 0.753 | | 0.194 | |  | | 1.12 | | (0.63 | - | 2.00) | 0.689 | 0.218 | |  | | 1.05 | (0.57 | - | 1.97) | 0.866 | 0.180 |  | 1.10 | (0.58 | - | 2.10) | 0.760 | 0.172 | |
| Weighted median | 1.01 | (0.46 | - | 2.22) | 0.971 | |  | |  | | 0.92 | | (0.42 | - | 2.01) | 0.831 |  | |  | | 0.97 | (0.39 | - | 2.38) | 0.943 |  |  | 0.91 | (0.37 | - | 2.21) | 0.832 |  | |
| Penalized weighted median | 0.99 | (0.46 | - | 2.10) | 0.975 | |  | |  | | 0.90 | | (0.39 | - | 2.10) | 0.812 |  | |  | | 0.93 | (0.40 | - | 2.20) | 0.873 |  |  | 0.88 | (0.35 | - | 2.19) | 0.778 |  | |
| MR-Egger: slope | 1.68 | (0.73 | - | 3.87) | 0.215 | |  | |  | | 1.63 | | (0.69 | - | 3.82) | 0.256 |  | |  | | 1.60 | (0.63 | - | 4.05) | 0.318 |  |  | 1.57 | (0.60 | - | 4.11) | 0.354 |  | |
| intercept | 0.97 | (0.92 | - | 1.02) | 0.164 | |  | |  | | 0.97 | | (0.92 | - | 1.02) | 0.241 |  | |  | | 0.97 | (0.92 | - | 1.02) | 0.235 |  |  | 0.97 | (0.92 | - | 1.03) | 0.331 |  | |

Table S5 (Continued)

| **GWASs analyzing CRP as a continuous variable which was naturally log-transformed (mg/L)** | | | | | | | | | | | | | | | | | | | | | | | | | | | | | |
| --- | --- | --- | --- | --- | --- | --- | --- | --- | --- | --- | --- | --- | --- | --- | --- | --- | --- | --- | --- | --- | --- | --- | --- | --- | --- | --- | --- | --- | --- |
|  | **All SNPs** | | | | | | | | | | | | | |  | **After exclusion of pleiotropic SNPs** | | | | | | | | | | | | | |
|  | **Stage 1**  **Adjustment for age and 10 PCs** | | | | | | |  | **Stage 2**  **Adjustment for covariates***  **in addition to age and 10PCs** | | | | | |  | **Stage 1**  **Adjustment for age and 10 PCs** | | | | | |  | **Stage 2**  **Adjustment for covariates***  **in addition to age and 10PCs** | | | | | | |
| **Analysis method** | **HR** | **(95% CI)** | | | | **p** | **p-het†** |  | **HR** | **(95% CI)** | | | **p** | **p-het†** |  | **HR** | **(95% CI)** | | | **p** | **p-het†** |  | **HR** | **(95% CI)** | | | **p** | **p-het†** |  |
|  | | | | | | | | | | | | | | | | | | | | | | | | | | | | | |
| **Cigarettes/d** | | | | | | | | | | | | | | | | | | | | | | | | | | | | | |
| **< 15** |  |  |  |  |  | |  |  |  |  |  |  |  |  |  |  |  |  |  |  |  |  |  |  |  |  |  |  |  |
| Inverse-variance weighted | 1.24 | (0.83 | - | 1.85) | 0.297 | | 0.942 |  | 1.40 | (0.93 | - | 2.10) | 0.103 | 0.959 |  | 1.20 | (0.76 | - | 1.87) | 0.429 | 0.907 |  | 1.39 | (0.88 | - | 2.18) | 0.150 | 0.934 |  |
| Weighted median | 1.25 | (0.67 | - | 2.33) | 0.483 | |  |  | 1.61 | (0.82 | - | 3.15) | 0.163 |  |  | 1.26 | (0.62 | - | 2.57) | 0.523 |  |  | 1.64 | (0.83 | - | 3.24) | 0.158 |  |  |
| Penalized weighted median | 1.25 | (0.66 | - | 2.36) | 0.489 | |  |  | 1.61 | (0.84 | - | 3.09) | 0.150 |  |  | 1.26 | (0.65 | - | 2.46) | 0.494 |  |  | 1.64 | (0.82 | - | 3.26) | 0.160 |  |  |
| MR-Egger: slope | 1.79 | (0.99 | - | 3.22) | 0.052 | |  |  | 2.03 | (1.12 | - | 3.65) | 0.020 |  |  | 1.90 | (0.99 | - | 3.63) | 0.053 |  |  | 2.25 | (1.17 | - | 4.30) | 0.016 |  |  |
| intercept | 0.97 | (0.94 | - | 1.01) | 0.092 | |  |  | 0.97 | (0.94 | - | 1.01) | 0.093 |  |  | 0.97 | (0.93 | - | 1.00) | 0.059 |  |  | 0.96 | (0.93 | - | 1.00) | 0.050 |  |  |
| **≥ 15** |  |  |  |  |  | |  |  |  |  |  |  |  |  |  |  |  |  |  |  |  |  |  |  |  |  |  |  |  |
| Inverse-variance weighted | 0.77 | (0.45 | - | 1.30) | 0.324 | | 0.049 |  | 0.79 | (0.47 | - | 1.30) | 0.341 | 0.177 |  | 0.87 | (0.49 | - | 1.53) | 0.614 | 0.065 |  | 0.90 | (0.53 | - | 1.53) | 0.700 | 0.290 |  |
| Weighted median | 0.64 | (0.32 | - | 1.27) | 0.199 | |  |  | 0.47 | (0.23 | - | 0.95) | 0.036 |  |  | 0.74 | (0.33 | - | 1.66) | 0.468 |  |  | 0.66 | (0.29 | - | 1.50) | 0.322 |  |  |
| Penalized weighted median | 0.53 | (0.26 | - | 1.07) | 0.078 | |  |  | 0.46 | (0.23 | - | 0.94) | 0.032 |  |  | 0.72 | (0.33 | - | 1.55) | 0.400 |  |  | 0.64 | (0.30 | - | 1.37) | 0.250 |  |  |
| MR-Egger: slope | 0.86 | (0.39 | - | 1.90) | 0.706 | |  |  | 0.82 | (0.39 | - | 1.75) | 0.603 |  |  | 1.07 | (0.46 | - | 2.53) | 0.868 |  |  | 1.05 | (0.47 | - | 2.33) | 0.909 |  |  |
| intercept | 0.99 | (0.95 | - | 1.04) | 0.703 | |  |  | 1.00 | (0.95 | - | 1.04) | 0.875 |  |  | 0.98 | (0.94 | - | 1.03) | 0.502 |  |  | 0.99 | (0.95 | - | 1.03) | 0.620 |  |  |
|  | | | | | | | | | | | | | | | | | | | | | | | | | | | | | |
| **Depressive symptoms¥** | | | | | | | | | | | | | | | | | | | | | | | | | | | | | |
| **< 0.06** |  |  |  |  |  | |  |  |  |  |  |  |  |  |  |  |  |  |  |  |  |  |  |  |  |  |  |  |  |
| Inverse-variance weighted | 0.98 | (0.70 | - | 1.38) | 0.925 | | 0.488 |  | 1.00 | (0.72 | - | 1.39) | 0.987 | 0.616 |  | 1.03 | (0.71 | - | 1.50) | 0.874 | 0.380 |  | 1.07 | (0.74 | - | 1.54) | 0.732 | 0.532 |  |
| Weighted median | 0.97 | (0.60 | - | 1.58) | 0.901 | |  |  | 1.00 | (0.60 | - | 1.68) | 0.999 |  |  | 1.00 | (0.60 | - | 1.68) | 0.998 |  |  | 1.01 | (0.61 | - | 1.68) | 0.975 |  |  |
| Penalized weighted median | 0.97 | (0.60 | - | 1.57) | 0.902 | |  |  | 1.00 | (0.60 | - | 1.66) | 1.000 |  |  | 1.00 | (0.60 | - | 1.68) | 0.991 |  |  | 1.01 | (0.59 | - | 1.74) | 0.975 |  |  |
| MR-Egger: slope | 1.16 | (0.70 | - | 1.91) | 0.568 | |  |  | 1.14 | (0.69 | - | 1.86) | 0.607 |  |  | 1.30 | (0.74 | - | 2.28) | 0.352 |  |  | 1.30 | (0.76 | - | 2.25) | 0.334 |  |  |
| intercept | 0.99 | (0.96 | - | 1.02) | 0.391 | |  |  | 0.99 | (0.96 | - | 1.02) | 0.477 |  |  | 0.98 | (0.95 | - | 1.01) | 0.267 |  |  | 0.99 | (0.96 | - | 1.02) | 0.321 |  |  |
| **≥ 0.06** |  |  |  |  |  | |  |  |  |  |  |  |  |  |  |  |  |  |  |  |  |  |  |  |  |  |  |  |  |
| Inverse-variance weighted | 0.99 | (0.20 | - | 4.82) | 0.987 | | 0.339 |  | 0.00 | (0.00 | - | 1.8E+05) | 0.334 | 0.000 |  | 1.28 | (0.22 | - | 7.36) | 0.780 | 0.278 |  | 0.00 | (0.00 | - | 8.3E+05) | 0.326 | 0.000 |  |
| Weighted median | 0.69 | (0.07 | - | 7.26) | 0.759 | |  |  | NA | NA | - | NA | NA |  |  | 0.73 | (0.06 | - | 8.58) | 0.803 |  |  | NA | NA | - | NA | NA |  |  |
| Penalized weighted median | 0.69 | (0.07 | - | 7.11) | 0.756 | |  |  | NA | NA | - | NA | NA |  |  | 0.73 | (0.06 | - | 8.68) | 0.803 |  |  | NA | NA | - | NA | NA |  |  |
| MR-Egger: slope | 4.67 | (0.47 | - | 46.48) | 0.184 | |  |  | NA | NA | - | NA | NA |  |  | 8.42 | (0.69 | - | 103.42) | 0.094 |  |  | NA | NA | - | NA | NA |  |  |
| intercept | 0.88 | (0.77 | - | 1.01) | 0.071 | |  |  | 0.09 | (0.01 | - | 0.73) | 0.025 |  |  | 0.86 | (0.75 | - | 1.00) | 0.045 |  |  | 0.08 | (0.01 | - | 0.76) | 0.029 |  |  |

BMI, body mass index; CI, confidence interval; CRP, C-reactive protein; E, exogenous estrogen; E+P, E plus progestin; GWAS, genome-wide association study; HR, hazard ratio; MET, metabolic equivalent; MR, Mendelian randomization; PCs, principal components; SFA, saturated fatty acids; SNP, single–nucleotide polymorphism. WHR, waist-to-hip ratio.

***** Covariates adjusted in the analyses for the association between genome-wide SNPs and breast cancer risk include education; annual family income; family history of breast cancer; BMI; waist-to-hip ratio; physical activity; depressive symptoms; number of cigarettes per day; dietary alcohol in g/day; % calories from SFA/day; age at menopause; duration of oral contraceptive use; and durations of exogenous estrogen [E]–only use and E plus progestin use; variables used to stratify were not included as covariates in the multivariate analysis.

† Heterogeneity in estimates among genome-wide SNPs was evaluated by using Cochran’s Q test with fixed effects.

¥ Depressive symptoms were estimated via a short form of the Center for Epidemiologic Studies Depression Scale.
