# Supplementary material for: Genetically Predicted C-Reactive Protein Associated With Postmenopausal Breast Cancer Risk: Interrelation With Estrogen and Cancer Molecular Subtypes Using Mendelian Randomization
Source: Front Oncol. 2021 Feb 3;10:630994. doi: 10.3389/fonc.2020.630994 (PMC7888276; doi:10.3389/fonc.2020.630994)
Supplement: Supplementary file 1 [file DataSheet_1.zip › TableS6_2020Nov18.docx]

Table S6. Mendelian randomization gene–environment (G×E) interaction using the interaction-variable subgroups with the scaled CRP-GS§

|  | **MR G×E slope** | | | | |  | **MR G×E intercept** | | | | | |
| --- | --- | --- | --- | --- | --- | --- | --- | --- | --- | --- | --- | --- |
| **G × E variable*** | **OR†** | **(95% CI)** | | | **p** |  | **OR†** | **95% CI** | | | **p** |  |
| **G × BMI** | 0.73 | (0.0002 | - | 3.30E+03) | 0.913 |  | 0.94 | (0.41 | - | 2.16) | 0.814 |  |
| **G × WHR** | 0.07 | (7.47E-07 | - | 6.83E+03) | 0.517 |  | 0.72 | (0.22 | - | 2.39) | 0.452 |  |
| **G × MET** | 0.73 | (1.08E-09 | - | 4.95E+08) | 0.954 |  | 0.96 | (0.10 | - | 9.27) | 0.946 |  |
| **G × SFA** | 0.0003 | (3.67E-10 | - | 3.22E+02) | 0.162 |  | 0.41 | (0.09 | - | 1.75) | 0.144 |  |
| **G × Depression score** | 8.23E+04 | (0.003 | - | 2.67E+12) | 0.106 |  | 3.38 | (0.63 | - | 18.08) | 0.089 |  |
| **G × Alcohol/day** | 720.44 | (1.81E-12 | - | 2.87E+17) | 0.577 |  | 1.89 | (0.05 | - | 65.44) | 0.609 |  |
| **G × Estrogen-only** | 9.55 | (1.01E-09 | - | 9.00E+10) | 0.775 |  | 1.13 | (0.14 | - | 8.85) | 0.863 |  |
| **G × Estrogen + progestin** | 0.95 | (1.89E-04 | - | 4.76E+03) | 0.986 |  | 0.95 | (0.56 | - | 1.62) | 0.789 |  |
| **G × OCuse** | 0.10 | (3.61E-10 | - | 2.72E+07) | 0.730 |  | 0.80 | (0.11 | - | 5.75) | 0.744 |  |

BMI, body mass index; CI, confidence interval; GS, weighted genetic score; MET, metabolic equivalent; OC, oral contraceptive; OR, odds ratio; SFA, saturated fatty acid; WHR, waist-to-hip ratio. Note: The MRG×E slope reflects an MR estimate that was corrected for G×E interaction with a modeled variable; the intercept reflects an estimate of pleiotropic effect.

§ The scaled CRP-GS was estimated on the basis of 56 CRP-SNPs whose effect size reflects the mean change in log-transformed CRP per allele.

* Analyzed interaction variables were continuous or ordinal variables with 5 or more categories.

† MR G×E results are from univariate analyses.
